# Correction: *Development and validation of the Family Resilience (FaRE) Questionnaire: an observational study in Italy*

**DOI:** 10.1136/bmjopen-2018-024670corr1

**Published:** 2019-09-04

Faccio F, Gandini S, Renzi C, *et al*. Development and validation of the Family Resilience (FaRE) Questionnaire: an observational study in Italy. *BMJ Open* 2019;9:e024670. doi: 10.1136/bmjopen-2018-024670

This article has been corrected since it was published.

Details were missing in affiliations 2 and 3. The correct affiliations are:

^2^Applied Division for Cognitive and Psychological Science, European Institute of Oncology IRCCS, Milan, Italy

^3^Experimental Oncology, European Institute of Oncology IRCCS, Milan, Italy

