# Supplementary material for: Development and validation of the Family Resilience (FaRE) Questionnaire: an observational study in Italy
Source: BMJ Open. 2019 Jun 5;9(6):e024670. doi: 10.1136/bmjopen-2018-024670 (PMC6561460; doi:10.1136/bmjopen-2018-024670)
Supplement: Supplementary file 1 [file bmjopen-2018-024670supp001.pdf]

Supplementary Material

Appendix A. *Tables of biological descriptive statistics of Study 1 and differential scores in patient-caregiver dyads*

Table 1. Tumor characteristics of the first sample

		Total (n=104)	Breast (n=53)	prostate (n=51)
Lymph nodes	No	80 (76.9)	40 (75.5)	40 (78.4)
	Yes	22 (21.2)	13 (24.5)	9 (17.6)
	Missing	2 (1.9)	0 (0)	2 (3.9)
Metastasis	No	101 (97.1)	53 (100)	48 (94.1)
	Yes	1 (1)	0 (0)	1 (2)
	Missing	2 (1.9)	0 (0)	2 (3.9)
Stage	I	15 (14.4)	11 (20.8)	4 (7.8)
	II	52 (50)	33 (62.3)	19 (37.3)
	III-IV	35 (33.7)	9 (17)	26 (51)
	Missing	2 (1.9)	0 (0)	2 (3.9)
Grading	1-2	39 (37.5)	26 (49.1)	13 (25.5)
	3-4	64 (61.5)	27 (50.9)	37 (72.5)
	Missing	1 (1)	0 (0)	1 (2)
Previous therapy	No	28 (26.9)	12 (22.6)	16 (31.4)
	Yes	75 (72.1)	41 (77.4)	34 (66.7)
	Missing	1 (1)	0 (0)	1 (2)

Table 2. Differences in family resilience scores in patient-caregiver dyads in the first study

**Differences pt-CG**	**Median**	**Low**	**Up**
factor1_com	0	-0.38	0.5
factor2_supp	0	-0.5	0.75
factor3_coping	0.13	-0.38	1
factor4_spirit	-0.25	-1	1

Low: lower quartile; Up: upper quartile

Factor1_com = Communication and cohesion; Factor2_supp= Perceived social support; Factor3_coping = Perceived family coping; Factor4_spirit = Religiousness and Spirituality

Table 3. Mean differential scores and paired-t test between patient-caregiver dyads in the second study

	Mean	SD	p-value

**diff_factor1**	**-0.27**	0.93	**0.003**
diff_factor2	0.21	1.17	0.074
diff_factor3	-0.14	1.05	0.179
diff_factor4	0.27	1.72	0.111

Factor1 = Communication and cohesion; Factor2= Perceived social support; Factor3= Perceived family coping; Factor4 = Religiousness and Spirituality

Table 4. Eingenvalues and proportion of variance of the factors

	Eigenvalue	Difference	Proportion	Cumulative
**1**	**13.72**	**9.92**	**0.39**	**0.39**
**2**	**3.81**	**0.77**	**0.11**	**0.50**
**3**	**3.04**	**0.35**	**0.09**	**0.58**
**4**	**2.69**	**0.66**	**0.08**	**0.66**
5	2.02	0.52	0.06	0.72
6	1.51	0.42	0.04	0.76
7	1.08	0.03	0.03	0.79
8	1.06	0.09	0.03	0.82
9	0.96	0.15	0.03	0.85
