# Supplementary material for: Development and validation of the Family Resilience (FaRE) Questionnaire: an observational study in Italy
Source: BMJ Open. 2019 Jun 5;9(6):e024670. doi: 10.1136/bmjopen-2018-024670 (PMC6561460; doi:10.1136/bmjopen-2018-024670)
Supplement: Author's manuscript [file bmjopen-2018-024670.draft_revisions.pdf]

BMJ Open is committed to open peer review. As part of this commitment we make the peer review history of every article we publish publicly available.

When an article is published we post the peer reviewers' comments and the authors' responses online. We also post the versions of the paper that were used during peer review. These are the versions that the peer review comments apply to.

The versions of the paper that follow are the versions that were submitted during the peer review process. They are not the versions of record or the final published versions. They should not be cited or distributed as the published version of this manuscript.

BMJ Open is an open access journal and the full, final, typeset and author-corrected version of record of the manuscript is available on our site with no access controls, subscription charges or pay-per-view fees (http://bmjopen.bmj.com).

If you have any questions on BMJ Open's open peer review process please email info.bmjopen@bmj.com

BMJ Open

# Development and validation of a Family Resilience questionnaire (FaRE)

| | |
|---|---|
| Journal: | *BMJ Open* |
| Manuscript ID | bmjopen-2018-024670 |
| Article Type: | Research |
| Date Submitted by the Author: | 07-Jun-2018 |
| Complete List of Authors: | Renzi, Chiara; European Institute of Oncology, European Institute of Oncology<br>Faccio, Flavia; Università degli Studi di Milano, Department of Oncology and Hemato-Oncology; Istituto Europeo di Oncologia, Applied Division for Cognitive and Psychological Science<br>Fioretti, Chiara; Università degli Studi di Firenze, Department of Educational Sciences and Psychology<br>Crico, Chiara; Università degli Studi di Milano, Department of Oncology and Hemato-Oncology; Istituto Europeo di Oncologia, Applied Division for Cognitive and Psychological Science<br>Gandini, Sara; European Institute of Oncology, Division of Epidemiology and Biostatistics<br>Pravettoni, Gabriella; Istituto Europeo di Oncologia, Applied Research Division for Cognitive and Psychological Science; Universita degli Studi di Milano, Department of Oncology and Hemato-oncology |
| Keywords: | Family Resilience, Stress and coping, Surveys and Questionnaires, Systems theory, ONCOLOGY, Family Adaptation |
| | |

SCHOLARONE™
Manuscripts

# Development and validation of a Family Resilience questionnaire (FaRE)

Chiara Renzi[1], Flavia Faccio[1,2], Chiara Fioretti[3], Chiara Crico[1,2], Sara Gandini[4], Gabriella Pravettoni[1,2]

[1] Applied Research Division for Cognitive and Psychological Science, European Institute of Oncology, Milan, Italy

[2] Department of Oncology and Hemato-Oncology, University of Milan, Milan, Italy

[3] Department of Educational Sciences and Psychology, Università degli Studi di Firenze, Florence, Italy

[4] Division of Epidemiology and Biostatistics, European Institute of Oncology, Milan, Italy

Email addresses: chiara.renzi@ieo.it; flavia.faccio@ieo.it; chiara.fioretti@unifi.it; chiara.crico@ieo.it; sara.gandini@ieo.it; gabriella.pravettoni@ieo.it

Word count: 3588

‡Corresponding author:

Chiara Renzi, chiara.renzi@ieo.it, Telephone: +39 029437099, Applied Research Unit for Cognitive and Psychological Science, European Institute of Oncology, Milan, Via Ripamonti 435.



For peer review only - http://bmjopen.bmj.com/site/about/guidelines.xhtml

**Abstract**

*Objective:* Develop and validate an instrument to assess family resilience and, more specifically, the family dynamics and resources, estimating the adaptation flexibility to cancer disease. Cohesion, communication, coping style, and relational style were considered as critical functional areas in the construction of the instrument.

*Design*: Two cross-sectional studies. Study 1: Identification of factorial structure of the questionnaire in two samples with different cancer sites. Study 2: Validation of the questionnaire in cancer patients in two different phases of their therapeutic pathway.

*Participants and setting:* A total of 213 patients with an histologically confirmed non-metastatic breast or prostate cancer and 209 caregivers were recruited for the two studies from an oncological hospital in Italy.

*Outcome measures*: The Resilience Scale for Adults and the Family Resilience questionnaire (FaRE) developed by the researchers were administered to all patients and caregivers that gave consent.

*Results:* In Study 1, the 60-item version of the FaRE underwent discriminant and construct validity, internal consistency, and factorial analysis. Comparisons between patient and caregiver populations showed that patients perceived higher levels of family resources ($p$=0.048) and that prostate cancer patients perceive less social support compared to breast cancer ($p$=0.002). Factor analysis demonstrated four domains: Communication and cohesion, Perceived social support, Perceived family coping, and Religiousness and spirituality. In Study 2 the validity and factorial structure of the final scale, composed of 24 items, were confirmed. The Cronbach's alpha of all subscales was above .82. Normative values for breast cancer patients can provide indications of family resilience levels.



For peer review only - http://bmjopen.bmj.com/site/about/guidelines.xhtml

*Conclusions:* The FaRE is a valid and reliable scale to measure family resilience in oncological patients and their caregivers. It can be used as a psycho-emotional monitoring tool and can be implemented in a web platform. Further research should test its usability in other medical contexts.

**Keywords**

Family Resilience, Family Adaptation, Stress and coping, Surveys and Questionnaires, Systems Theory, Oncology

**Article summary**

***Strengths and limitations of this study***

- Questionnaire development and testing was conducted in two phases in order to assess validity and reliability of the final version of the measure.
- This is the first measure of family resilience that adopts a systemic approach, considering both the patient and caregiver, in light of increasing evidence on the vital role the caregiver.
- Results were controlled for the possible confounding sociodemographic variables.
- Only two oncological populations were recruited, making generalisability to all types of tumour patients limited.
- Longitudinal measurements are advised to investigate test-retest reliability of the measure.



For peer review only - http://bmjopen.bmj.com/site/about/guidelines.xhtml

For peer review only

## Introduction

Resilience is a multifaceted concept that can be described as the ability to mobilize resources and adapt to challenging or adverse situations [1,2]. More and more often, resilience is studied as a relevant aspect when facing a disease such as cancer [3–5]. However, the impact of disrupting events such as a cancer diagnosis, is not limited only to the individual, rather it influences also their family and social network, which can initiate or support positive adaptation as well as be overwhelmed by the demands of the disease and its treatments. According to the family systems theory [6], a change or a perturbation occurring to one member of the system will affect also the other members. Life events may result in a smooth adaptation to a new homeostasis or may result in difficulties and hindrances that prevent the readjustment process [6]. This translates into clinically significant levels of distress, higher risk of developing psychosocial problems, high levels of conflict and low family cohesion [7]. Going beyond the idea that families which encounter difficulties are "damaged" or "pathological" [8], resilience may represent a pivotal concept to analyse and to support the family in moving towards a new balance and construct meaning of the cancer event [9].

A substantial contribution to research in family resilience comes from Froma Walsh's works. Walsh [8,10] defines it as the family's ability to "withstand and rebound from adversity, strengthened and more resourceful", including the capacity to cope and adapt to the stressor and the concept of post-traumatic growth. She considers three major dimensions as contributing to the system's resilience, each composed of three subprocesses: i) belief systems, ii) organizational patterns and iii) communication processes. These are considered mutually interactive and synergistic as they facilitate and sustain each other across systems and over time [10].

Although family resilience is a vital construct both in clinical and research settings, only three questionnaires have attempted to capture the multiple dimensions of Walsh's framework. Sixbey [11] developed a 66-item measure, the Family Resilience Assessment Scale (FRAS), on a 4-point Likert scale divided into 6 subscales: i) family communication and problem-solving, ii) social and



For peer review only - http://bmjopen.bmj.com/site/about/guidelines.xhtml

economic resources, iii) maintaining a positive outlook, iv) family connectedness, v) family spirituality and vi) making meaning of adversity. The questionnaire was administered to the general American population without considering an essential aspect of resilience, namely whether an adversity had occurred. The development study argued that a nine construct model of family resilience was not supported and therefore decreased the number of factors to six [11]. Within its study the FRAS showed good reliability and validity and has been adapted in different languages [12]. It has been applied recently to families with child diagnosed with autism spectrum disorder [13] and its psychometric properties have been evaluated in a sample of epileptic young adults [14], confirming good reliability and validity in assessing family resilience.

The other two measures developed according to Walsh's framework are the Family Resilience Assessment (FRA; Lane & Meszaros, 2016) and the Walsh Family Resilience Questionnaire (WFRQ; Walsh, 2013). The former was validated in a sample of women with a history of breast cancer and the items were adapted to this population. Lane and colleagues [15] warned that the items did not always reflect Walsh's indicators and that socio-economic resources had little value in breast cancer survivors. To date the FRA has not been used in other oncological or more general health contexts, suggesting the need for further studies in order to consider the instrument a valid measure of Walsh's model. Finally, Walsh [16] developed her own self-report questionnaire, composed of 33 items that operationalise her nine key processes and provide a map of each family. While she states that her questionnaire is being applied in several international projects in diverse contexts, such as chronic illness, disaster recovery and immigration, data from these studies has not been published yet.

As there is only one measure of family resilience in cancer patients [11] which has an individualistic view of resilience and does not capture the systemic processes involved, a new measure was developed. The aim of this measure is to investigate family resources and strengths during cancer management, taking into account both the patient's and the caregiver's perspective. Driven by



For peer review only - http://bmjopen.bmj.com/site/about/guidelines.xhtml

Walsh's [8] theoretical framework and Sixbey's [11] work on a family resilience measure, the questionnaire considered six subprocesses as key in fostering family resilience.

## Material and methods

Two studies were carried out. The first to identify the structure of the Family Resilience Evaluation (FaRE) questionnaire in the field of resilience and cancer, assessing two different cancer sites. The second study was designed to validate the questionnaire in cancer patients in two different phases of their therapeutic pathway.

### 1. First study: Item generation phase and development of the questionnaire

A first version of the Family Resilience Evaluation (FaRE) questionnaire was constructed by the authors after a thorough revision of scientific literature in the field of resilience and cancer. The questionnaire was composed of 60 items referring to the six aspects: 1) family communication and problem solving, 2) social and economic resources, 3) maintaining a positive outlook, 4) family connectedness, 5) family spirituality, and 6) ability to make meaning from adversity. Questions were on a seven-point Likert scale (from Strongly Agree to Strongly Disagree).

The questionnaire starts with a brief sentence that explains to patients and caregivers how to complete it: "*We kindly ask you to read carefully each of the following statements. For each, please define how well it describes your family. When referring to your family, you can include all those people that you consider significant and that you feel are part of your family*".

<u>1.1 Face validity</u>

A pilot study with 10 citizens (healthy individuals between 25 and 80 years of age, with no diagnosis of cognitive or psychological disorders) and with 20 breast and prostate cancer patients was performed to assess the first version of the FaRE questionnaire, and to evaluate the degree of clarity and comprehension of the questionnaire's items. Once they had read the first version of the FaRE, the participants indicated those items which resulted unclear, providing suggestions or



For peer review only - http://bmjopen.bmj.com/site/about/guidelines.xhtml

comments to the researchers. This lead to rewording some items and clarifying the meaning of some statements.

## 1.2 Item selection phase

### *1.2.1 Participants*

The study was proposed to couples of patients and caregivers that fitted the inclusion criteria. Participation was proposed to a total of 146 couples, 42 of which refused due to little interest in the study or lack of time. 105 patients (53 breast, 52 prostate cancer patients) and 105 caregivers (i.e., son/daughter, wife/husband/partner, father/mother, sister/brother) were recruited for the study at a comprehensive cancer centre in northern Italy. Once the study's aims and procedures were illustrated, all participants gave written informed consent.

Inclusion criteria for patients were the following: 1) between 25 and 80 years of age; 2) non-metastatic cancer diagnosis of breast or prostate cancer; 4) no psychiatric, addictive, or cognitive disorders that would prevent compliance with protocol requirements.

The inclusion criteria for caregivers were: 1) over 18 years old; 2) had a relative with a diagnosis of breast or prostate cancer; 3) no psychiatric, addictive, or cognitive disorders that would prevent compliance with protocol requirements.

Due to incomplete answers of one patient during the consultation one patient was drop-out and the final sample was composed by 104 patients and 105 caregivers..

In order to carry out a validation study for this questionnaire, a proposal to the local Ethical Committee was submitted. Ethical approval was obtained on April 11th, 2016.

### *1.2.2 Materials*

The revised version of the FaRE, obtained at the end of the pilot study, was composed of 60 items and it was administered to patients and caregivers participating to the study.



For peer review only - http://bmjopen.bmj.com/site/about/guidelines.xhtml

The Italian version of the Resilience Scale for Adults (RSA) [17] was chosen to assess the psychometric characteristics of the FaRE. The RSA is a 33 item self-report scale for adults that measures six resilience factors: Perception of Self, Planned Future, Social Competence, Structured Style, Family Cohesion, and Social Resources. It provides a total mean score, with higher scores indicating higher resilience. Four factors measure the individual's characteristics, one the family environment and the last factor social networks. Cronbach's alphas for the Italian version ranged from 0.75 to 0.90 for all subscales and total score with the exception of Structured Style (0.34), which showed poor reliability.

*1.2.3 Patient and public involvement*

The patients in this study were participants. They were not involved in the development of the research question or in the recruitment phase. However, patients were asked for feedback on the readability of the questionnaire in the pilot phase and their suggestions were incorporated in the adopted version of the FaRE.

*1.2.4 Statistical analyses*

The first step was an exploratory factor analysis (EFA) to reduce the item pool [18]. The EFA was conducted using principal axis factoring with varimax rotation. Several criteria provided guidelines in selecting the number of factors to extract [19]. Factors with eigenvalues greater than 1.0 were retained and an examination of scree plots of eigenvalues and total variance explained by retained factors helped to determine the total number of factors to retain. More generally, in the early stages and throughout the entire process, the items extracted were the ones that provided the "cleanest" and most "interpretable" factor loadings.

A Cronbach coefficient alpha was calculated for the entire scale and for each individual subscale to assure internal consistency and reliability.



For peer review only - http://bmjopen.bmj.com/site/about/guidelines.xhtml

To evaluate the goodness of fit between the models and data we used fit indices, including the comparative fit index (CFI) and the RMSEA that indicates the amount of unexplained variance.

Discriminative and divergent validity were made comparing factors between patients and caregivers, age groups, levels of education, marital status, and number of children by Wilcoxon rank tests.

## *1.2.5 Results*

### *1.2.5.1 Descriptive statistics*

The median age of the population was 60 years old, with an interquartile range (IQR) from 51 to 66. Sixty percent of caregivers were female. The majority of the participants was married or in a relationship (83.7% and 81% for patients and caregivers respectively) and had children (84.6% and 76.2% for patients and caregivers respectively). The majority of participants had a high school degree (41.3% for patients, 44% for caregivers) or higher (33.7% for participants, 29% for caregivers). No sociodemographic variables were significantly different between groups. Further descriptive data can be found in Table 1.

Table 1. Sociodemographic characteristics of the sample

| | | Total (N=209) | Patients (N=104) | CG (N=105) | P-values |
|---|---|---|---|---|---|
| Age | <50, n (%) | 43 (20.6) | 22 (21.2) | 21 (20) | 0.84 |
| | ≥50, n (%) | 162 (77.5) | 80 (76.9) | 82 (78.1) | |
| | Missing, n (%) | 4 (1.9) | 2 (1.9) | 2 (1.9) | |
| Gender | Male, n (%) | 93 (44.5) | 51 (49) | 42 (40) | 0.19 |
| | Female, n (%) | 116 (55.5) | 53 (51) | 63 (60) | |
| Education | Elementary-middle, n (%) | 49 (23.4) | 23 (22.1) | 26 (24.8) | 0.76 |
| | High school, n (%) | 90 (43.1) | 43 (41.3) | 47 (44.8) | |
| | University, n (%) | 66 (31.6) | 35 (33.7) | 31 (29.5) | |
| | Missing, n (%) | 4 (1.9) | 3 (2.9) | 1 (1) | |
| Marital status | Single*, n (%) | 35 (16.7) | 17 (16.3) | 18 (17.1) | 0.83 |
| | Married, n (%) | 172 (82.3) | 87 (83.7) | 85 (81) | |
| | Missing, n (%) | 2 (1) | 0 (0) | 2 (1.9) | |
| Children | No, n (%) | 39 (18.7) | 16 (15.4) | 23 (21.9) | 0.36 |
| | 1-2, n (%) | 133 (63.6) | 68 (65.4) | 65 (61.9) | |
| | >2, n (%) | 35 (16.7) | 20 (19.2) | 15 (14.3) | |



For peer review only - http://bmjopen.bmj.com/site/about/guidelines.xhtml

For peer review only

| | | | |
|---|---|---|---|
| Missing, n (%) | 2 (1) | 0 (0) | 2 (1.9) |

CG: Caregiver; *Single: never married or widow or separated

Around 83% of breast and 45% of prostate cancer patients were diagnosed with Stage I-II, the remaining patients had stage III-IV tumours. All breast and most prostate patients had undergone at least one type of oncological therapy before (radiotherapy, chemotherapy and/or surgery). One prostate patient underwent PET scan for tumour stadiation several weeks after completing the questionnaires and the test indicated the presence of metastases. Further clinical data can be found in Supplementary Material.

*1.2.5.2 Factorial analysis*

Factorial analysis indicates 4 factors: one factor of the initial version of the questionnaire was removed ("Ability to make meaning from adversity") and we collapsed two factors in which items were overlapping, namely "Family communication and problem solving" and "Family connectedness" (see Supplementary material).

A sufficient model fit (Comparative Fit Index- CFI) was defined as of 0.90 or greater, the RMSEA of 0.06 or less. The CFI was 0.94, the RMSEA Estimate 0.05, indicating a good model of fit between the model and the data.

The final four factors were named: "Communication and cohesion", "Perceived social support", "Perceived family coping", "Religiousness and spirituality". For each factor, the items with the highest weights were selected. The number of items for factors Communication and cohesion and Perceived social support is eight, four items for the other two factors (Perceived family coping, Religiousness and spirituality).

*1.2.5.3 Internal consistency*



For peer review only - http://bmjopen.bmj.com/site/about/guidelines.xhtml

An alpha of 0.70 is nowadays considered as acceptable for a new instrument [20]. The alpha computed for each of the four subscales exceeded the minimum value for a new tool: Cronbach's alphas for each factor were 0.88, 0.88, 0.82, and 0.86, respectively.

*1.2.5.4 Construct validity*

In order to assess construct validity of FaRE, we analyzed whether the subscales of the questionnaire correlated with the RSA using Spearman correlations. Total scores of FaRE and RSA were positively correlated (ρs= 0.41, $p<0.0001$). Factor Communication and cohesion correlated positively with factor Family Cohesion in RSA (ρs= 0.48, $p<0.0001$). Factor Perceived social support correlated positively with factor Social Resources (ρs= 0.46, $p<0.0001$). Factor Perceived family coping correlated positively with factor Structured Style (ρs= 0.20, $p=0.005$) and with factor Perception of Self of the RSA (ρs= 0.38, $p<0.0001$).

*1.2.5.5 Discriminant validity*

*1.2.5.5.1 Comparison between patients and caregivers*

Perceived family coping was significantly different between patients and caregivers, the former perceiving higher levels of family resources (median score patients=6.3; median score caregivers=6.0; $p=0.048$). A comparison was carried out also within patient-caregiver dyads and the difference was not significant for any factor (see Supplementary Material).

*1.2.5.5.2 Comparison between cancer patients' populations*

Perceived social support was significantly different between breast and prostate cancer patients. The former reported receiving greater support from family and friends for their disease compared to the latter (median score breast cancer patients=6.3; median score prostate cancer patients=5.5, $p=0.002$). Table 2 details the results of the non-parametric tests.

Table 2. Non-parametric tests on FaRe factors

| | Patients (n=104) | | | | CG (n=105) | | | | |
|---|---|---|---|---|---|---|---|---|---|
| **Factors** | **Median** | **Stdev** | **Q1** | **Q3** | **Median** | **Stdev** | **Q1** | **Q3** | **P-values** |



For peer review only - http://bmjopen.bmj.com/site/about/guidelines.xhtml

| | | | | | | | | | |
|---|---|---|---|---|---|---|---|---|---|
| Communication and cohesion | 6.6 | 0.9 | 5.7 | 7.5 | 6.5 | 0.8 | 5.7 | 7.3 | 0.238 |
| Perceived social support | 5.9 | 1.2 | 4.7 | 7.0 | 5.8 | 1.1 | 4.6 | 6.9 | 0.542 |
| Perceived family coping | 6.3 | 0.8 | 5.4 | 7.1 | 6.0 | 1.0 | 5.0 | 7.0 | **0.048** |
| Religiousness and Spirituality | 4.5 | 1.8 | 2.7 | 6.3 | 4.8 | 1.9 | 2.9 | 6.6 | 0.341 |

| | **Breast (n=53)** | | | | **Prostate (n=51)** | | | | |
|---|---|---|---|---|---|---|---|---|---|
| **Factors** | **Median** | **Stdev** | **Q1** | **Q3** | **Median** | **Stdev** | **Q1** | **Q3** | **P-values** |
| Communication and cohesion | 6.6 | 0.7 | 5.9 | 7.4 | 6.8 | 1.1 | 5.7 | 7.8 | 0.180 |
| Perceived social support | **6.3** | **0.8** | **5.4** | **7.1** | **5.5** | **1.3** | **4.2** | **6.8** | **0.002** |
| Perceived family coping | 6.0 | 0.9 | 5.1 | 6.9 | 6.5 | 0.7 | 5.8 | 7.2 | 0.171 |
| Religiousness and Spirituality | 4.8 | 1.8 | 3.0 | 6.5 | 4.3 | 1.9 | 2.3 | 6.2 | 0.285 |

P-values from Wilcoxon rank tests. Stdev: Standard deviation. Q1 and Q3 are the first and third quartiles.

### *1.2.6 Adopted version of questionnaire*

The final factors of the family resilience questionnaire are four. Communication and cohesion refers to the family's openness in communicating about the illness and its impact on their daily life. It also involves shared problem-solving and decision-making, resolution of conflict, openness regarding the range of feelings they are experiencing. The second factor, Perceived social support, refers to mutual support, reaching out to extended kin and community for help with practical and emotional tasks. The third factor, Perceived family coping, includes the ability to activate coping strategies and develop inner strength to deal with illness management. The concept of bouncing back and the ability to rebound from this stressful life event is present in this subscale. The last factor, Religiousness and spirituality, refers to spiritual values, transcendent beliefs and congregational support, which helps some families in finding meaning and purpose to the cancer diagnosis.

This questionnaire was back-translated for use in an English hospital, therefore both Italian and English versions are available.

## 2. Second study: Validation of the questionnaire

In order to validate the factorial structure of the questionnaire, the previously adopted version of the questionnaire was administered to a new sample of breast cancer patients in two different phases of


For peer review only
For peer review only - http://bmjopen.bmj.com/site/about/guidelines.xhtml

their therapeutic pathway: pre-admission phase (either for day-surgery admission or for ordinary surgery admission) and treatment phase (surgery, radiation therapy, or chemotherapy).

*2.1 Participants*

A total of 143 couples was approached to explain the study, 34 couples refused due to time constraints between appointments or due to little interest in participating. Only breast cancer patients were recruited at this time due to easier access to patient waiting rooms, higher number of accesses in the hospital and higher compliance.

109 breast patients from a comprehensive cancer center in northern Italy and 104 family caregivers (i.e., son/daughter, husband/partner, father/mother, sister/brother) were recruited for the study. Four caregivers were not able to come to the consultation on the scheduled day and thus could not provide informed consent. Therefore, they were considered as drop-outs.

*2.2 Materials*

The adopted version of the FaRE obtained at the end of Study 1, which consisted of 24 items, was administered to patients and caregivers (see Supplementary Material). As in Study 1, the RSA scale [17] was chosen to assess the psychometric characteristics of the FaRE.

*2.3 Results*

*2.3.1 Descriptive statistics*

The median age of the population was 49 and IQR from 43 to 56. Thirty-three percent of the caregivers were female. Most participants were married or in a relationship (80% and 74% for patients and caregivers respectively) and had children (95% and 92% for patients and caregivers respectively). The majority of the participants had a high school degree or higher (82% for patients, 82% for caregivers). More than half of the patients were not undergoing oncological therapy at time of recruitment and were diagnosed with Stage I-II breast cancer. Further descriptive information can be found in Table 3.



For peer review only - http://bmjopen.bmj.com/site/about/guidelines.xhtml

Table 3. Descriptive statistics of the second sample

| | | Overall (N=213) | Patients (n=109) | Caregivers (n=104) |
|---|---|---|---|---|
| Age | <50, n (%) | 116 (54) | 109 (51) | 104 (49) |
| | ≥50, n (%) | 97 (46) | 60 (55) | 56 (54) |
| Children | 0, n (%) | 47 (22) | 49 (45) | 48 (46) |
| | 1-2, n (%) | 153 (72) | 22 (20) | 25 (24) |
| | >3, n (%) | 13 (6) | 82 (75) | 71 (68) |
| Marital status | Single*, n (%) | 44 (21) | 20 (18) | 24 (23) |
| | Married, n (%) | 164 (77) | 87 (80) | 77 (74) |
| | Missing, n (%) | 5 (2) | 2 (2) | 3 (3) |
| Educational level | Elementary-middle school, n (%) | 38 (18) | 20 (18) | 18 (17) |
| | High school, n (%) | 92 (43) | 41 (38) | 51 (49) |
| | University, n (%) | 82 (38) | 48 (44) | 34 (33) |
| | Missing, n (%) | 1 (0) | 0 (0) | 1 (1) |
| Stage | 0, n (%) | | 11 (10.1) | |
| | I, n (%) | | 52 (47.7) | |
| | II, n (%) | | 25 (22.9) | |
| | III, n (%) | | 20 (18.8) | |
| Previous therapy | Surgery, n (%) | | 42 (38.5) | |
| | Chemotherapy, n (%) | | 19 (17.4) | |
| | Hormone therapy, n (%) | | 2 (1.8) | |
| | No therapy, n (%) | | 46 (42.2) | |
| Ongoing therapy | Chemotherapy, n (%) | | 16 (14.7) | |
| | Hormone therapy, n (%) | | 13 (11.9) | |
| | Radiotherapy, n (%) | | 10 (9.2) | |
| | No therapy, n (%) | | 70 (64.2) | |

*Single: never married or widow or separated

### *2.3.2 Factorial analysis*

The Bentler Comparative Fit Index was 0.88, the RMR Estimate 0.07, indicating a good model of fit between the model and the data.

### *2.3.3 Construct validity*

In order to confirm the construct validity of the final version of FaRE, Spearman correlations of the factors of the FaRE questionnaire with the RSA subscales were conducted. Total scores of FaRE and RSA were positively correlated (ρs= 0.43, $p$<0.0001). Factor Communication and cohesion correlated positively with factor Family Cohesion in RSA (ρs= 0.56, $p$<0.0001). Factor Perceived social support correlated positively with factor Social Resources (ρs= 0.54, $p$<0.0001). Factor

For peer review only

Perceived family coping correlated positively with factor Structured Style (ρs= 0.30, *p*<0.0001) and with factor Perception of Self in RSA (ρs= 0.42, *p*<0.0001).

*2.3.4 Comparison between patients and caregivers*

Perceived Social Support was significantly different between patients and caregivers, the former perceiving greater support than the latter (median score patients=6.4; median score caregivers=6.0; *p*=0.03).

*2.3.5 Concordance between patients' and caregivers' scores*

In this analysis, only scores of patients for which a corresponding caregiver's score was available were included. Differential scores were computed for each factor by subtracting the score of the patient from that of the corresponding caregiver. Paired t-tests indicated that the difference for Factor Communication and Cohesion was significantly different from zero (mean difference= -0.27, standard deviation= 0.93, *p*=0.003). No other significant differences were detected (see Supplementary Material).

*2.3.6 Differences between Study 1 and Study 2 samples*

Wilcoxon signed rank tests indicated no differences between the median values of factors in sample in Study 1 and the sample in Study 2, both when considering together the patients and the caregivers, as well as when the patients and the caregivers were compared separately.

*2.3.7 Normative data*

Due to disparity in number between prostate and breast cancer patients, normative data was calculated on the total breast cancer patients recruited for the two studies. Table 4 illustrates mean and standard deviation for each subscale and for the total FaRE score. As with other clinical measures, scores between -/+ 1 SD are considered in the normal range, scores between -1 and -2 SD indicate lower levels of family resilience and scores under 2 SD are considered clinically relevant. For the last two ranges of scores referral to support services is recommended.



For peer review only - http://bmjopen.bmj.com/site/about/guidelines.xhtml

Table 4. Normative family resilience scores for breast cancer patients

| | N | Mean | Standard deviation |
|---|---|---|---|
| **Communication and cohesion** | 317 | 6.30 | 0.85 |
| **Perceived social support** | 317 | 5.86 | 1.04 |
| **Perceived family coping** | 317 | 6.14 | 0.89 |
| **Religiousness and Spirituality** | 317 | 4.52 | 1.93 |
| **Mean total score** | 317 | 5.70 | 0.78 |

## 3. Discussion

The FaRE questionnaire is designed to measure family resilience in medical settings. Adopting a systemic approach, it considers both the patient and the caregiver's strengths and resources in managing the disease. This tool is useful both in clinical and research context as increasing attention is being paid to resilience and its protective role in illness trajectories. The questionnaire confirms Sixbey's [11] assumption that the nine-factor family resilience model developed by Walsh [8] can be reduced; in our sample four factors best captured resilience processes, in Sixbey's [11] normal population six factors were considered essential. Lane's [21] family resilience measure had highlighted that social and economic resources had little value to breast cancer survivors and our measure confirms this, even though the two oncological populations are slightly different, Lane's [21] sample has already overcome the stressful event while here patients are at the beginning or in the middle of their therapeutic pathway. The sample size, the use of a standardised measure to compare factors, the goodness of fit between model and data, and the high internal consistency are some of the strengths of the study. Another strength of the measure is that it can be administered to caregivers, for whom there are only a few questionnaires available; attention towards their challenges and needs has increased only in the last few years [22–24]. In addition to this, the tool was also designed to be implemented in a web platform as a part of the works of an European project, which aimed at promoting cancer patient's empowerment and self-management [25–27].

The only statistical difference between breast and prostate cancer patients scores was in the Perceived social support subscale. It is difficult to interpret this finding, as it might be gender-



For peer review only - http://bmjopen.bmj.com/site/about/guidelines.xhtml

related, tumour-specific or due to older age of prostate patients. Further analyses should investigate whether perception of social support varies in different oncological patients. Patients perceived significantly higher levels of family coping compared to their caregivers; although the difference in scores was small, it could be explained by the re-direction and allocation of shared coping strategies towards the patient rather than towards the caregiver. The same could be occurring in the second study, where patients perceived higher levels of social support compared to caregivers. Further research should try and disentangle whether lower support and family coping scores of caregivers are of clinical significance, by assessing the association with mood profiles for instance. The analysis on patient-caregiver dyad of the second study, showed that caregivers reported significantly lower levels of open communication and emotional sharing compared to the corresponding patient. This a preliminary result specific to breast cancer patients, however it might suggest that caregivers supress their emotions and find it harder to communicate about the illness, which in turn can lead to psychological distress [28].

To strengthen the rigor of the questionnaire for further research, the researchers recommend undertaking convergent and discriminant validity to examine further similarities and differences between the FaRE and other family resilience tools. It is also recommended that confirmatory factor analysis be undertaken in a larger sample with diverse tumour diagnosis, applying it also to non-gender related tumours, as well as people with other illnesses to support the generalisability of the questionnaire. As this instrument is a useful tool to assess patients at baseline and for continued follow-up, test-retest reliability would be advised. As resilience is a process rather than a dispositional trait [1,29], comparisons between different phases of the illness (e.g. diagnosis, treatment, and follow-up) can provide insight into which resources are activated and whether “natural” low or high points of resilience occur.



For peer review only - http://bmjopen.bmj.com/site/about/guidelines.xhtml

Considering that both breast and prostate cancer patients have relatively good prognosis and high rates of 5-year survival, it would be interesting to investigate whether family resilience levels in other big killers and in metastatic diseases are equivalent or differ substantially.

While the FaRE does not substitute psychological or other clinical appointments, this measure can serve as a useful screening tool and identify individuals with little resources to deal with cancer related issues already in an early phase of the therapeutic pathway and plan interventions accordingly.

For peer review only

**Funding**

This project has received funding from the European Union's Horizon 2020 research and innovation programme under grant agreement No 643529.

**Disclaimer**

This presentation reflects the authors' view. The Commission is not responsible for any use that may be made of the information it contains.

**Declaration of interest**

We have read and understood the BMJ Group policy on declaration of interests and we declare no conflict of interest.

**Author Contributions**

All authors have made substantial contributions to the conception or design of the work, SG in particular to the analysis or interpretation of data for the work; All authors contributed to drafting the work or revising it critically for important intellectual content; All authors approved the final version to be published and agree to be accountable for all aspects of the work in ensuring that questions related to the accuracy or integrity of any part of the work are appropriately investigated and resolved.

**Data sharing statement**

The datasets generated and analysed during the current study are available from the corresponding author on reasonable request.

**Acknowledgements**

We thank patients for providing feedback and suggestions for improving the family resilience tool.



For peer review only - http://bmjopen.bmj.com/site/about/guidelines.xhtml

**References**

1. Rutter M. Implications of resilience concepts for scientific understanding. *Ann N Y Acad Sci*. 2006;1094:1-12. doi:10.1196/annals.1376.002

2. Masten AS. Ordinary magic: Resilience processes in development. *Am Psychol*. 2001;56(3):227-238. doi:10.1037//0003-066X.56.3.227

3. Strauss B, Brix C, Fischer S, et al. The influence of resilience on fatigue in cancer patients undergoing radiation therapy (RT). *J Cancer Res Clin Oncol*. 2007;133(8):511-518. doi:10.1007/s00432-007-0195-z

4. Wenzel LB, Donnelly JP, Fowler JM, et al. Resilience, reflection, and residual stress in ovarian cancer survivorship: A gynecologic oncology group study. *Psychooncology*. 2002;11(2):142-153. doi:10.1002/pon.567

5. Min JA, Yoon S, Lee CU, et al. Psychological resilience contributes to low emotional distress in cancer patients. *Support Care Cancer*. 2013;21(9):2469-2476. doi:10.1007/s00520-013-1807-6

6. Von Bertalanffy L. General system theory. In: *The Science of Synthesis: Exploring the Social Implications of General Systems Theory*. Boulder, Colorado: University Press of Colorado; 2003:304.

7. Van Schoors M, Caes L, Verhofstadt LL, Goubert L, Alderfer MA. Systematic review: Family resilience after pediatric cancer diagnosis. *J Pediatr Psychol*. 2015;40(9):856-868. doi:10.1093/jpepsy/jsv055

8. Walsh F. Family Resilience: A Framework for Clinical Practice. *Fam Process*. 2003;42(I):1-18. doi:10.1111/famp.2003.42.issue-1



For peer review only - http://bmjopen.bmj.com/site/about/guidelines.xhtml

9. Faccio F, Renzi C, Giudice A V., Pravettoni G. Family Resilience in the Oncology Setting: Development of an Integrative Framework. *Front Psychol*. 2018;9(666). doi:10.3389/fpsyg.2018.00666

10. Walsh F. Applying a Family Resilience Framework in Training, Practice, and Research: Mastering the Art of the Possible. *Fam Process*. 2016;55(4):616-632. doi:10.1111/famp.12260

11. Sixbey MT. Development of the family resilience assessment scale to identify family resilience constructs. *Dr Diss Univ Florida*. 2005.

12. Isaacs SA, Roman NV, Savahl S, Sui X-C. Adapting and Validating the Family Resilience Assessment Scale in an Afrikaans Rural Community in South Africa. *Community Ment Health J*. 2017;0(0):0. doi:10.1007/s10597-017-0091-1

13. Duca DS. Family resilience and parental stress: the effects on marital relationship in the context of a child diagnosed with an autism spectrum disorder. *Ann AII Cuza Univ Psychol Ser*. 2015;24(1):71-91. http://repository.upenn.edu/edissertations_sp2%0Ahttp://repository.upenn.edu/edissertations_sp2/14.

14. Chew J, Haase AM. Psychometric properties of the Family Resilience Assessment Scale: A Singaporean perspective. *Epilepsy Behav*. 2016;61:112-119. doi:10.1016/j.yebeh.2016.05.015

15. Lane CD, Meszaros PS. Measuring Walsh ' s Family Resilience Framework : Reliability and Validity of the Family Resilience Assessment ( FRA ) Among Women With a History of Breast Cancer. *Marriage Fam Rev*. 2016:1-16.

16. Walsh F. *Strengthening Family Resilience*.; 2013. doi:10.1086/514469

For peer review only

17. Capanna C, Stratta P, Hjemdal O, Collazzoni A, Rossi A. The Italian validation study of the Resilience Scale for Adults (RSA). *Appl Psychol Bull*. 2015;272(63):16-24. http://search.ebscohost.com/login.aspx?direct=true&db=psyh&AN=2015-19177-002&site=ehost-live&scope=site.

18. Fabrigar LR, Wegener DT, Maccallum RC, Strahan EJ. Evaluating the Use of Exploratory Factor Analysis in Psychological Research. *Psychol Methods*. 1999;4(3):272-299.

19. Hatcher RL, Barends A, Hansell J, Gutfreund MJ. Patients' and Therapists' Shared and Unique Views of the Therapeutic Alliance: An Investigation Using Confirmatory Factor Analysis in a Nested Design. *J Consult Clin Psychol*. 1995;63(4):636-643.

20. DeVon HA, Block ME, Moyle-Wright P, et al. A Psychometric Toolbox for Testing Validity and Reliability. *J Nurs Scholarsh*. 2007;39(2):155-164. doi:10.1111/j.1547-5069.2007.00161.x

21. Meszaros PS, Allen KR, Few-demo AL, Piercy FP, Savla JS. Tracing the Pink Ribbon : Development of a Family Resilience Measure Crystal Lynn Duncan Lane Dissertation submitted to the faculty of the Virginia Polytechnic Institute and State University in partial fulfillment of the requirements of the degree of Doct. 2011.

22. Kim Y, Baker F, Spillers RL, Wellisch DK. Psychological adjustment of cancer caregivers with multiple roles. *Psychooncology*. 2006;15(9):795-804. doi:10.1002/pon.1013

23. Sklenarova H, Krümpelmann A, Haun MW, et al. When do we need to care about the caregiver? Supportive care needs, anxiety, and depression among informal caregivers of patients with cancer and cancer survivors. *Cancer*. 2015;121(9):1513-1519. doi:10.1002/cncr.29223

24. Marzorati C, Renzi C, Russell-Edu W, Pravettoni G. A systematic review of telemedicine utilization in caregivers of cancer patients (Preprint). *J Med Internet Res*. January 2018.



For peer review only - http://bmjopen.bmj.com/site/about/guidelines.xhtml

doi:10.2196/jmir.9812

25. Kazantzaki E, Koumakis L, Kondylakis H, et al. Current trends in Electronic Family Resilience Tools: Implementing a tool for the cancer domain. In: ; 2018:29-32. doi:10.1007/978-981-10-5122-7_8

26. Renzi C, Fioretti C, Mazzocco K, et al. Development of psycho-emotional monitoring tools within an eHealth platform to improve patient empowerment and self-management abilities. *Psychooncology*. 2016;25(3):195.

27. Kazantzaki E, Kondylakis H, Koumakis L, et al. Psycho-emotional tools for better treatment adherence and therapeutic outcomes for cancer patients. *Stud Health Technol Inform*. 2016;224:129-134. https://air.unimi.it/retrieve/handle/2434/395837/640655/2016_Kazantzaki et al.%2C Conference paper.pdf. Accessed August 3, 2017.

28. Haun MW, Sklenarova H, Brechtel A, Herzog W, Hartmann M. Distress in Cancer Patients and Their Caregivers and Association with the Caregivers' Perception of Dyadic Communication. *Oncol Res Treat*. 2014;37(7-8):5-5. doi:10.1159/000364885

29. American Psychological Association. The road to resilience. American Psychological Association. http://www.apa.org/helpcenter/road-resilience.aspx. Published 2014.



For peer review only - http://bmjopen.bmj.com/site/about/guidelines.xhtml

Figure 1. Eingenvalues and proportion of variance of the factors

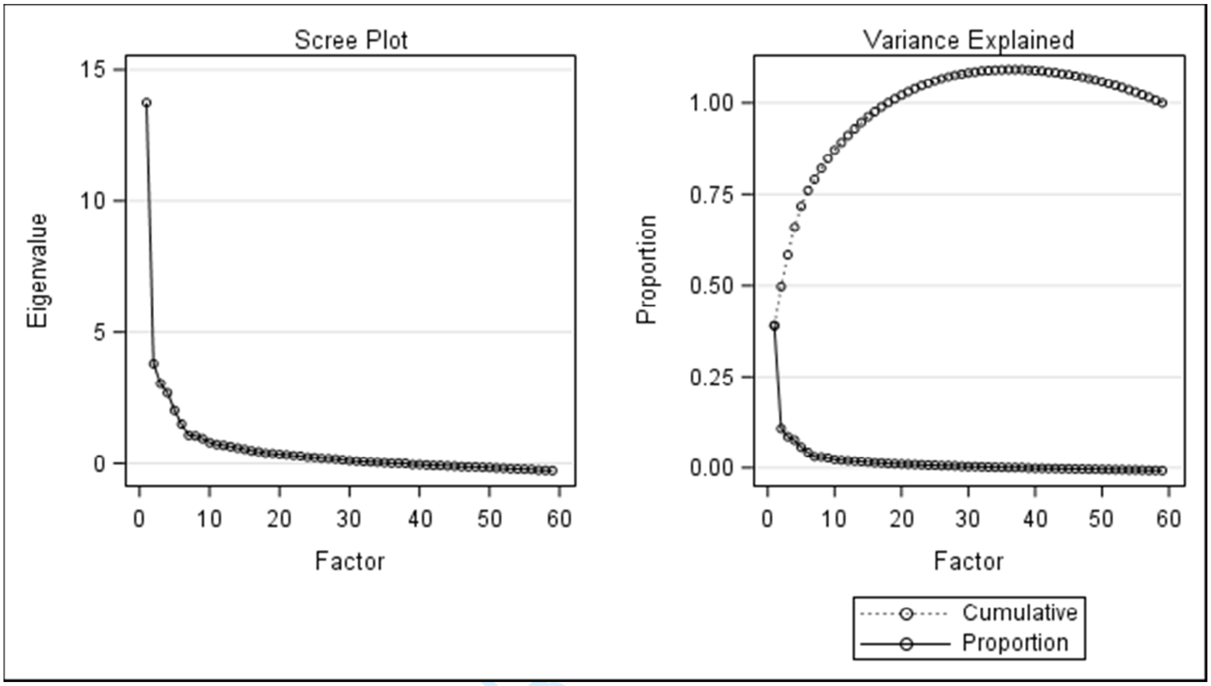


For peer review only

**Supplementary Material**

Appendix A. *Family Resilience Questionnaire*

Dear Sir/Madam,

We kindly ask you to read carefully each of the following statements. For each of these, please indicate how well these describe your family. When thinking about your family, you can include all the significant people that according to you are part of the family.

1. We understand each other about the illness experience that we are living

Strongly disagree Moderately disagree Slightly disagree Nor Disagree nor agree Slightly Agree Moderately Agree Strongly agree

2. We ask our closest friends to help and assist us in this battle against the illness

Strongly disagree Moderately disagree Slightly disagree Nor Disagree nor agree Slightly Agree Moderately Agree Strongly agree

3. We believe that we can manage the illness

Strongly disagree Moderately disagree Slightly disagree Nor Disagree nor agree Slightly Agree Moderately Agree Strongly agree

4. In our family we feel that we can talk about how to communicate between us

Strongly disagree Moderately disagree Slightly disagree Nor Disagree nor agree Slightly Agree Moderately Agree Strongly agree

5. We can count on close friends to help us face this illness

Strongly disagree Moderately disagree Slightly disagree Nor Disagree nor agree Slightly Agree Moderately Agree Strongly agree



For peer review only - http://bmjopen.bmj.com/site/about/guidelines.xhtml

6. We go to church/synagogue/mosque/other religious places

Strongly disagree Moderately disagree Slightly disagree Nor Disagree nor agree Slightly Agree Moderately Agree Strongly agree

7. We can face this illness as a family

Strongly disagree Moderately disagree Slightly disagree Nor Disagree nor agree Slightly Agree Moderately Agree Strongly agree

8.We feel that the people in our social network would be happy to support us emotionally in facing the illness

Strongly disagree Moderately disagree Slightly disagree Nor Disagree nor agree Slightly Agree Moderately Agree Strongly agree

9. We can work out the significant difficulties in our life such as this illness

Strongly disagree Moderately disagree Slightly disagree Nor Disagree nor agree Slightly Agree Moderately Agree Strongly agree

10. We think about the illness-related problems until we find a shared solution

Strongly disagree Moderately disagree Slightly disagree Nor Disagree nor agree Slightly Agree Moderately Agree Strongly agree

11. We feels that our closest friends would be happy to support us emotionally in managing the illness

Strongly disagree Moderately disagree Slightly disagree Nor Disagree nor agree Slightly Agree Moderately Agree Strongly agree

12. We believe that a spiritual being will help us face this illness



For peer review only

Strongly disagree Moderately disagree Slightly disagree Nor Disagree nor agree Slightly Agree Moderately Agree Strongly agree

13. Everyone in the family feels free to express their own opinion regarding the illness

Strongly disagree Moderately disagree Slightly disagree Nor Disagree nor agree Slightly Agree Moderately Agree Strongly agree

14. We know that if we need comfort from our closest friends they will be there for us

Strongly disagree Moderately disagree Slightly disagree Nor Disagree nor agree Slightly Agree Moderately Agree Strongly agree

15. We feel we are strong enough to face this illness

Strongly disagree Moderately disagree Slightly disagree Nor Disagree nor agree Slightly Agree Moderately Agree Strongly agree

16. We are honest when talking about the illness between ourselves

Strongly disagree Moderately disagree Slightly disagree Nor Disagree nor agree Slightly Agree Moderately Agree Strongly agree

17. We know that we are important for our friends

Strongly disagree Moderately disagree Slightly disagree Nor Disagree nor agree Slightly Agree Moderately Agree Strongly agree

18. We participate in the activities of the religious community

Strongly disagree Moderately disagree Slightly disagree Nor Disagree nor agree Slightly Agree Moderately Agree Strongly agree



For peer review only

19. Everyone in the family is open to listening other's opinions regarding the illness

Strongly disagree Moderately disagree Slightly disagree Nor Disagree nor agree Slightly Agree Moderately Agree Strongly agree

20. We receive presents and favours from our closest friends

Strongly disagree Moderately disagree Slightly disagree Nor Disagree nor agree Slightly Agree Moderately Agree Strongly agree

21. We have the strength to solve our problems

Strongly disagree Moderately disagree Slightly disagree Nor Disagree nor agree Slightly Agree Moderately Agree Strongly agree

22. The things we do for each other to face this illness make us feel part of the family

Strongly disagree Moderately disagree Slightly disagree Nor Disagree nor agree Slightly Agree Moderately Agree Strongly agree

23. Our friends respect our family for how we reacted to the illness

Strongly disagree Moderately disagree Slightly disagree Nor Disagree nor agree Slightly Agree Moderately Agree Strongly agree

24. We ask for advice or words of comfort for the illness to our religious/spiritual reference figure

Strongly disagree Moderately disagree Slightly disagree Nor Disagree nor agree Slightly Agree Moderately Agree Strongly agree



For peer review only - http://bmjopen.bmj.com/site/about/guidelines.xhtml

Appendix B. *Tables of biological descriptive statistics of Study 1 and differential scores in patient-caregiver dyads*

Table 1. Tumor characteristics of the first sample

| | | Total (n=104) | Breast (n=53) | prostate (n=51) |
|---|---|---|---|---|
| Lymph nodes | No | 80 (76.9) | 40 (75.5) | 40 (78.4) |
| | Yes | 22 (21.2) | 13 (24.5) | 9 (17.6) |
| | Missing | 2 (1.9) | 0 (0) | 2 (3.9) |
| Metastasis | No | 101 (97.1) | 53 (100) | 48 (94.1) |
| | Yes | 1 (1) | 0 (0) | 1 (2) |
| | Missing | 2 (1.9) | 0 (0) | 2 (3.9) |
| Stage | I | 15 (14.4) | 11 (20.8) | 4 (7.8) |
| | II | 52 (50) | 33 (62.3) | 19 (37.3) |
| | III-IV | 35 (33.7) | 9 (17) | 26 (51) |
| | Missing | 2 (1.9) | 0 (0) | 2 (3.9) |
| Grading | 1-2 | 39 (37.5) | 26 (49.1) | 13 (25.5) |
| | 3-4 | 64 (61.5) | 27 (50.9) | 37 (72.5) |
| | Missing | 1 (1) | 0 (0) | 1 (2) |
| Previous therapy | No | 28 (26.9) | 12 (22.6) | 16 (31.4) |
| | Yes | 75 (72.1) | 41 (77.4) | 34 (66.7) |
| | Missing | 1 (1) | 0 (0) | 1 (2) |

Table 2. Differences in family resilience scores in patient-caregiver dyads in the first study

| **Differences pt-CG** | **Median** | **Low** | **Up** |
|---|---|---|---|
| factor1_com | 0 | -0.38 | 0.5 |
| factor2_supp | 0 | -0.5 | 0.75 |
| factor3_coping | 0.13 | -0.38 | 1 |
| factor4_spirit | -0.25 | -1 | 1 |

Low: lower quartile; Up: upper quartile

Factor1_com = Communication and cohesion; Factor2_supp= Perceived social support; Factor3_coping = Perceived family coping; Factor4_spirit = Religiousness and Spirituality

Table 3. Mean differential scores and paired-t test between patient-caregiver dyads in the second study

| | Mean | SD | p-value |
|---|---|---|---|
| **diff_factor1** | **-0.27** | 0.93 | **0.003** |



For peer review only - http://bmjopen.bmj.com/site/about/guidelines.xhtml

| | | | |
|---|---|---|---|
| diff_factor2 | 0.21 | 1.17 | 0.074 |
| diff_factor3 | -0.14 | 1.05 | 0.179 |
| diff_factor4 | 0.27 | 1.72 | 0.111 |

Factor1 = Communication and cohesion; Factor2= Perceived social support; Factor3= Perceived family coping; Factor4 = Religiousness and Spirituality

Table 4. Eingenvalues and proportion of variance of the factors

| | Eigenvalue | Difference | Proportion | Cumulative |
|---|---|---|---|---|
| **1** | **13.72** | **9.92** | **0.39** | **0.39** |
| **2** | **3.81** | **0.77** | **0.11** | **0.50** |
| **3** | **3.04** | **0.35** | **0.09** | **0.58** |
| **4** | **2.69** | **0.66** | **0.08** | **0.66** |
| 5 | 2.02 | 0.52 | 0.06 | 0.72 |
| 6 | 1.51 | 0.42 | 0.04 | 0.76 |
| 7 | 1.08 | 0.03 | 0.03 | 0.79 |
| 8 | 1.06 | 0.09 | 0.03 | 0.82 |
| 9 | 0.96 | 0.15 | 0.03 | 0.85 |

For peer review only

BMJ Open

# Development and validation of a Family Resilience questionnaire (FaRE): an observational study in Italy

| | |
|---|---|
| Journal: | *BMJ Open* |
| Manuscript ID | bmjopen-2018-024670.R1 |
| Article Type: | Research |
| Date Submitted by the Author: | 17-Oct-2018 |
| Complete List of Authors: | Faccio, Flavia; Università degli Studi di Milano, Department of Oncology and Hemato-Oncology; Istituto Europeo di Oncologia, Applied Division for Cognitive and Psychological Science<br>Renzi, Chiara; European Institute of Oncology, European Institute of Oncology<br>Fioretti, Chiara; Università degli Studi di Firenze, Department of Educational Sciences and Psychology<br>Crico, Chiara; Università degli Studi di Milano, Department of Oncology and Hemato-Oncology; Istituto Europeo di Oncologia, Applied Division for Cognitive and Psychological Science<br>Gandini, Sara; European Institute of Oncology, Division of Epidemiology and Biostatistics<br>Pravettoni, Gabriella; Istituto Europeo di Oncologia, Applied Research Division for Cognitive and Psychological Science; Universita degli Studi di Milano, Department of Oncology and Hemato-oncology |
| <b>Primary Subject Heading</b>: | Patient-centred medicine |
| Secondary Subject Heading: | General practice / Family practice, Oncology, Research methods, Patient-centred medicine |
| Keywords: | Family Resilience, Stress and coping, Surveys and Questionnaires, Systems theory, ONCOLOGY, Family Adaptation |
| | |

SCHOLARONE™
Manuscripts

# Development and validation of a Family Resilience questionnaire (FaRE): an observational study in Italy

*Flavia Faccio[1,2]‡, *Chiara Renzi[1], Chiara Fioretti[3], Chiara Crico[1,2], Sara Gandini[4], Gabriella Pravettoni[1,2]

[1] Applied Research Division for Cognitive and Psychological Science, IEO, European Institute of Oncology IRCCS, Milan, Italy

[2] Department of Oncology and Hemato-Oncology, University of Milan, Milan, Italy

[3]Department of Educational Sciences and Psychology, Università degli Studi di Firenze, Florence, Italy

[4]Division of Epidemiology and Biostatistics, IEO, European Institute of Oncology IRCCS, Milan, Italy

* These authors equally contributed to the manuscript

Email addresses: flavia.faccio@ieo.it; chiara.renzi@ieo.it; chiara.fioretti@unifi.it; chiara.crico@ieo.it; sara.gandini@ieo.it; gabriella.pravettoni@ieo.it

Word count: 4616

‡Corresponding author:



For peer review only

For peer review only - http://bmjopen.bmj.com/site/about/guidelines.xhtml

Flavia Faccio, flavia.faccio@ieo.it, Telephone: +39 029437099, Applied Research Unit for Cognitive and Psychological Science, European Institute of Oncology, Milan, Via Ripamonti 435.

**Abstract**

*Objective:* Develop and validate an instrument to assess family resilience and, more specifically, the family dynamics and resources, estimating the adaptation flexibility to cancer disease. Cohesion, communication, coping style, and relational style were considered as critical functional areas in the construction of the instrument.

*Design*: Two cross-sectional studies. Study 1: Identification of factorial structure of the questionnaire in two samples with different cancer sites. Study 2: Validation of the questionnaire in cancer patients in two different phases of their therapeutic pathway.

*Participants and setting:* A total of 213 patients with a histologically confirmed non-metastatic breast or prostate cancer and 209 caregivers were recruited for the two studies from an oncological hospital in Italy.

*Outcome measures*: The Resilience Scale for Adults and the Family Resilience questionnaire (FaRE) developed by the researchers were administered to all patients and caregivers that gave consent.

*Results:* In Study 1, the 60-item version of the FaRE underwent discriminant and construct validity, internal consistency, and factorial analysis. Comparisons between patient and caregiver populations showed that patients perceived higher levels of family resources ($p$=0.048) and that prostate cancer patients perceive less social support compared to breast cancer ($p$=0.002). Factor analysis demonstrated four domains: Communication and cohesion, Perceived social support, Perceived family coping, and Religiousness and spirituality. In Study 2 the validity and factorial structure of the final scale, composed



For peer review only - http://bmjopen.bmj.com/site/about/guidelines.xhtml

of 24 items, were confirmed. The Cronbach's alpha of all subscales was above .82. Normative values for breast cancer patients can provide indications of family resilience levels.

*Conclusions:* Preliminary findings showed acceptable psychometric properties for the FaRE to evaluate family resilience in oncological patients and their caregivers. Further research should test its sensibility to change to assess its use as a psycho-emotional monitoring too and its validity in other medical contexts.

**Keywords**

Family Resilience, Family Adaptation, Stress and coping, Surveys and Questionnaires, Systems Theory, Oncology

**Article summary**

***Strengths and limitations of this study***

- Questionnaire development and testing was conducted in two phases in order to assess validity and reliability of the final version of the measure.
- This is the first measure of family resilience that adopts a systemic approach, considering both the patient and caregiver, in light of increasing evidence on the vital role the caregiver.
- Results were controlled for the possible confounding sociodemographic variables.
- Only two oncological populations were recruited, making generalisability to all types of tumour patients limited.
- Longitudinal measurements are advised to investigate test-retest reliability and responsiveness to change of the measure.



For peer review only - http://bmjopen.bmj.com/site/about/guidelines.xhtml

For peer review only

**Introduction**

Resilience is a multifaceted concept that can be described as the ability to mobilize resources and adapt to challenging or adverse situations [1,2]. One of the internal resources of resilience is coping, which consists of a set of modifiable skills that can be learnt to deal with a stressful life event[3]. More and more often, resilience is studied as a relevant aspect when facing a disease such as cancer [4–6]. However, the impact of disrupting events such as a cancer diagnosis, is not limited only to the individual, rather it influences also their family and social network, which can initiate or support positive adaptation as well as be overwhelmed by the demands of the disease and its treatments. According to the family systems theory [7], a change or a perturbation occurring to one member of the system will affect also the other members, which may result in a smooth adaptation to a new homeostasis or in difficulties that prevent the readjustment process [7]. This translates into clinically significant levels of distress, higher risk of developing psychosocial problems, high levels of conflict and low family cohesion[8]. Going beyond the idea that families which encounter difficulties are "damaged" or "pathological" [9], resilience may represent a pivotal concept to analyse and support the family in moving towards a new balance and in constructing meaning of the cancer event [10].

A substantial contribution to research in family resilience comes from two main models: the Resiliency Model of Family Adjustment and Adaptation[11] and the Family Resilience Framework[9], both of which



For peer review only - http://bmjopen.bmj.com/site/about/guidelines.xhtml

have been applied to oncological settings. Walsh's framework was chosen for various reasons: first, conceptualisation of family resilience in McCubbin's model is quite rigid, it belongs to a first wave of resilience research that believed one has to progress through specific steps in order to achieve resilience. In addition to this, due to the large number of variables involved in the model, it was possible for previous studies to validate only parts of it, never in its entirety[12]. Differently, Walsh [9,13] defines resilience as the family's ability to "withstand and rebound from adversity, strengthened and more resourceful", including the capacity to cope and adapt to the stressor and the concept of post-traumatic growth. She considers three major dimensions as contributing to the system's resilience: i) belief systems, ii) organizational patterns and iii) communication processes. Each of these overarching constructs is composed of three subprocesses: Make meaning of adversity, Positive outlook, Transcendence and spirituality, Flexibility, Connectedness, Social and economic resources, Communication/problem-solving, Clarity, Open emotional expression, Collaborative problem-solving. These are considered mutually interactive and synergistic as they facilitate and sustain each other across systems and over time [13]. Walsh also proposed an integrative family resilience model for illness related challenges, including cancer[14]; currently, there is no quantitative evidence regarding the framework's efficacy.

Only three questionnaires have attempted to capture the multiple dimensions of Walsh's[9] framework. Sixbey [15] developed the Family Resilience Assessment Scale (FRAS), a 66-item measure with responses given on a 4-point Likert scale. The Statistical considerations and factor loadings showed that a nine construct model of family resilience was not supported and therefore the number of factors was reduced to six [15]: i) family communication and problem-solving, ii) social and economic resources, iii) maintaining a positive outlook, iv) family connectedness, v) family spirituality and vi) making meaning of adversity. One of the subscales, Communication and Problem-solving, is an overarching construct of Walsh's model, which originally contained three sub-processes The questionnaire was administered to



For peer review only - http://bmjopen.bmj.com/site/about/guidelines.xhtml

the general American population without considering an essential aspect of resilience, namely whether an adversity had occurred. A second validation study suggested that the FRAS could be a reliable and valid measure in a population which was not experiencing an adversity [16]. This is the major weakness of the measure, which has then been administered to families that have experienced difficult diagnoses, such as autism spectrum disorder [17] and epilepsy [18], where disagreement regarding the number of factors of the scale is highlighted[18]. In addition to this, the measure was never administered to multiple members of a family, therefore the perspective of the questionnaire is individualistic and is not representative of the family unit as a whole. As individual perceptions of which resilience resources are activated may vary, it is essential to compare the views of different family members[19].

The other two measures developed according to Walsh's framework are the Family Resilience Assessment (FRA; Lane & Meszaros, 2016) and the Walsh Family Resilience Questionnaire (WFRQ; Walsh, 2013). The former is composed of 29 items on a 5-point scale response and was validated in a sample of women with a history of breast cancer. The developers [20] warned that the items did not always reflect Walsh's indicators, decided to group the items in the three overarching dimensions and suggested that socio-economic resources have little value in breast cancer survivors. Lane implies that further studies should capture the pluralistic view of the family members[20]. To date the FRA has not been used in other oncological or more general health contexts, suggesting the need for further studies in order to consider the instrument a valid measure. In addition to this, the recruited sample was small and different subgroups were present: survivors who had received diagnosis in the past 15 years, others in the past 5 years, another group of women undergoing treatment, of which half has a stage IV breast cancer diagnosis. Therefore, both current and retrospective accounts of resilience were collected. Finally, Walsh [19] developed her own self-report questionnaire, composed of 33 items that operationalise her nine key processes and provide a map of each family. While she states that her questionnaire is being applied in several international projects, data from these studies has not been published yet.



For peer review only - http://bmjopen.bmj.com/site/about/guidelines.xhtml

As there is only one measure of family resilience in cancer patients [15] which has an individualistic view of resilience and does not capture the systemic processes involved, a new measure including Sixbey's six factors was developed. These factors were chosen compared to Lane's three factors as, in our opinion, not all salient aspects of family resilience in cancer had been considered and to avoid construct underrepresentation, one of the major threats to construct validity[21]. The aim of this measure is to investigate family resources and strengths during cancer management, taking into account both the patient's and the caregiver's perspective.

## Material and methods

All study phases were presented in a single protocol to the local Ethics Committee, which was approved in all its parts on the 11th of April, 2016. The aim of the first study was to develop a questionnaire, the Family Resilience Evaluation Questionnaire (FaRE) to assess resilience in families affected by cancer. Following Messick's[21] guidelines for validating a measure, the following stages were conducted: substantive, structural, and external. While substantive and structural steps were undertaken in Study 1, the external stage is present in Study 2.

### 1. Study 1: Item generation phase and development of the questionnaire

For the substantive stage, a first version of the FaRE was constructed by the authors after a thorough revision of scientific literature in the field of resilience and cancer and after conducting informal, clinical appointments with 20 cancer patients about strategies that helped them adjust and cope with the illness. The questionnaire was then developed and assessed by an expert panel composed of psychologists that conduct research in the field of resilience. Some minor adjustments to the questions was made and five items were dropped as they were considered too similar to other statements. Finally, this preliminary version of the FaRE was administered to a pilot group of 10 citizens, 10 cancer patients and 10 caregivers



For peer review only - http://bmjopen.bmj.com/site/about/guidelines.xhtml

comparable to the target population in order to evaluate item comprehensibility, provide suggestions and indicate which items seemed unclear. Six items were re-worded to improve understanding. The second version of the questionnaire was composed of 60 items referring to six aspects: 1) family communication and problem solving, 2) social and economic resources, 3) maintaining a positive outlook, 4) family connectedness, 5) family spirituality, and 6) ability to make meaning from adversity. Questions were on a seven-point Likert scale (from Strongly Agree to Strongly Disagree).

## 1.2 Item selection phase

*1.2.1 Participants*

Couples of patients and caregivers that fitted the inclusion criteria were asked to participate in the current study. Participation was proposed to a total of 146 couples, 42 of which refused due to little interest in the study or lack of time. 105 patients (53 breast, 52 prostate cancer patients) and 105 caregivers (i.e., son/daughter, wife/husband/partner, father/mother, sister/brother) were recruited for the study at a comprehensive cancer centre in northern Italy. Patients were recruited from two outpatient clinics, one of the Breast Cancer Division, the second of the Urology Division. In addition to this, patients receiving oncological treatment were recruited from the Radiotherapy Division and Day Hospital. If compatible with the researcher's availability, consecutive patients who were waiting for their appointment were enrolled in the study. Once the study's aims and procedures were illustrated, all participants gave written informed consent.



For peer review only - http://bmjopen.bmj.com/site/about/guidelines.xhtml

Inclusion criteria for patients were the following: 1) between 25 and 80 years of age; 2) non-metastatic cancer diagnosis of breast or prostate cancer; 4) no psychiatric, addictive, or cognitive disorders that would prevent compliance with protocol requirements.

The inclusion criteria for caregivers were: 1) over 18 years old; 2) had a relative with a diagnosis of breast or prostate cancer; 3) no psychiatric, addictive, or cognitive disorders that would prevent compliance with protocol requirements.

Due to incomplete answers of one patient his data was not included in the database, therefore the final sample was composed by 104 patients and 105 caregivers.

### *1.2.2 Materials*

The revised version of the FaRE, obtained at the end of the pilot study, was composed of 60 items and it was administered to patients and caregivers participating to the study.

The Italian version of the Resilience Scale for Adults (RSA) [22] was chosen to assess the convergent validity of the FaRE. The RSA is a 33 item self-report scale for adults that measures six resilience factors: Perception of Self, Planned Future, Social Competence, Structured Style, Family Cohesion, and Social Resources. It provides a total mean score, with higher scores indicating higher resilience. Four factors measure the individual's characteristics, one the family environment and the last factor social networks. Cronbach's alphas for the Italian version ranged from 0.75 to 0.90 for all subscales and total score with the exception of Structured Style (0.34), which showed poor reliability. *1.2.3 Patient involvement and procedure*

The patients in this study were participants. They were not involved in the development of the research question or in the recruitment phase. However, patients were asked for feedback on the readability of the questionnaire in the pilot phase and their suggestions were incorporated in the final version of the FaRE.



For peer review only - http://bmjopen.bmj.com/site/about/guidelines.xhtml

Participants were approached by the researcher while they were in the waiting hall for the appointment with a healthcare professional (oncologist, surgeon, or nurse). The researcher explained the purposes of the study and asked both the patient and the caregiver if they were interested in participating. If they answered affirmatively, an appointment was made after their medical consultation, during which further details were given, together with an informed consent that was signed by both the researcher and the participants. The questionnaires were administered on an iPad; for those who did not use electronic devices paper-and-pencil versions of the questionnaires were given.

*1.2.4 Statistical analyses*

All statistical analyses were conducted using SAS (Statistical Analysis System) version 9.2. The first step was an exploratory factor analysis (EFA) to reduce the item pool [23]. The EFA was conducted using maximum likelihood factoring with orthomax rotation. Several criteria provided guidelines in selecting the number of factors to extract [24]. Factors with eigenvalues greater than 1.0 were retained and an examination of scree plots of eigenvalues and total variance explained by retained factors helped to determine the total number of factors to retain (see Figure 1).More generally, in the early stages and throughout the entire process, the items retained were those with the highest explained variance for each factor and those that belonged together.

A Cronbach coefficient alpha was calculated for the entire scale and for each individual subscale to assure internal consistency and reliability.

To evaluate the goodness of fit between the models and data we used fit indices, including the comparative fit index (CFI) and the RMSEA that indicates the amount of unexplained variance.

Total and subscale FaRE scores were compared between patients and caregivers, age groups, levels of education, marital status, and number of children by Wilcoxon rank tests. We did not expect differences between sociodemographic categories while we expected that differences could arise between patients



For peer review only - http://bmjopen.bmj.com/site/about/guidelines.xhtml

and caregivers, as activation of resources can differ depending on the role of the family member in adjusting to the illness. *1.2.5 Results*

*1.2.5.1 Descriptive statistics*

The median age of the population was 60 years old, with an interquartile range (IQR) from 51 to 66.

Sixty percent of caregivers were female. 71% of caregivers were wife/husband, 14% were son/daughter, 11% were sister/brother, and the remaining 4% were mother/father to the patient. The majority of the participants was married or in a relationship (83.7% and 81% for patients and caregivers respectively) and had children (84.6% and 76.2% for patients and caregivers respectively). Most participants had a high school degree (41.3% for patients, 44% for caregivers) or higher (33.7% for participants, 29% for caregivers). No sociodemographic variables were significantly different between groups. Further descriptive data can be found in Table 1.

Table 1. Sociodemographic characteristics of the sample

| | | Total (N=209) | Patients (N=104) | CG (N=105) | P-values |
|---|---|---|---|---|---|
| Age | <50, n (%) | 43 (20.6) | 22 (21.2) | 21 (20) | 0.84 |
| | ≥50, n (%) | 162 (77.5) | 80 (76.9) | 82 (78.1) | |
| | Missing, n (%) | 4 (1.9) | 2 (1.9) | 2 (1.9) | |
| Gender | Male, n (%) | 93 (44.5) | 51 (49) | 42 (40) | 0.19 |
| | Female, n (%) | 116 (55.5) | 53 (51) | 63 (60) | |
| Education | Elementary-middle, n (%) | 49 (23.4) | 23 (22.1) | 26 (24.8) | 0.76 |
| | High school, n (%) | 90 (43.1) | 43 (41.3) | 47 (44.8) | |
| | University, n (%) | 66 (31.6) | 35 (33.7) | 31 (29.5) | |
| | Missing, n (%) | 4 (1.9) | 3 (2.9) | 1 (1) | |
| Marital status | Single*, n (%) | 35 (16.7) | 17 (16.3) | 18 (17.1) | 0.83 |
| | Married, n (%) | 172 (82.3) | 87 (83.7) | 85 (81) | |
| | Missing, n (%) | 2 (1) | 0 (0) | 2 (1.9) | |
| Children | No, n (%) | 39 (18.7) | 16 (15.4) | 23 (21.9) | 0.36 |
| | 1-2, n (%) | 133 (63.6) | 68 (65.4) | 65 (61.9) | |
| | >2, n (%) | 35 (16.7) | 20 (19.2) | 15 (14.3) | |



For peer review only - http://bmjopen.bmj.com/site/about/guidelines.xhtml

| | | | |
|---|---|---|---|
| Missing, n (%) | 2 (1) | 0 (0) | 2 (1.9) |

CG: Caregiver; *Single: never married or widow or separated

Around 83% of breast and 45% of prostate cancer patients were diagnosed with Stage I-II, the remaining patients had stage III-IV tumours. All breast and most prostate patients had undergone at least one type of oncological therapy before (radiotherapy, chemotherapy and/or surgery). One prostate patient underwent PET scan for tumour stadiation several weeks after completing the questionnaires and the test indicated the presence of metastases. Further clinical data can be found in the Supplementary Material.

*1.2.5.2 Factorial analysis*

Kaiser Meyer Olkin's test for sampling adequacy was conducted on the sample to evaluate the adequacy of data. All values were above 0.8 (overall KMO= 0.92, patient sample KMO= 0.9, caregiver sample KMO=0.84), indicating that the data was suitable for factorial analysis[25]. Multigroup factor analysis was conducted to test for weak and strong factorial invariance across patients and caregivers. Strong factorial invariance was not supported, while weak factorial invariance suggested acceptable measurement invariance (patients SRMR =0.079, caregivers SRMR= 0.080). According to Kang and colleagues[26], weak factorial invariance is enough to proceed with group comparisons.

Explained variance was over 10% for 4 factors, for the remaining two factors the explained variance was under 5%, which is considered too low to be retained and were therefore excluded. Factor 5 had only two items, Factor 6 three and in both factors the items were unrelated to each other. EFA indicated 4 factors: one factor (Factor 5) of the initial version of the questionnaire was removed ("Ability to make meaning from adversity") and some items of two factors, namely "Family communication and problem solving" and "Family connectedness" were collapsed into a new subscale, Communication and Cohesion (see Supplementary material).



For peer review only - http://bmjopen.bmj.com/site/about/guidelines.xhtml

A sufficient model fit (Comparative Fit Index- CFI) was defined as of 0.90 or greater, the RMSEA of 0.06 or less. The CFI was 0.94, the RMSEA Estimate 0.05, indicating an adequate fit between the model and the data.

The final four factors were named: "Communication and cohesion", "Perceived social support", "Perceived family coping", "Religiousness and spirituality". For each factor, the items with the highest weights were selected. The number of items for factors Communication and cohesion and Perceived social support is eight, four items for the other two factors.

*1.2.5.3 Internal consistency*

An alpha of 0.70 is nowadays considered as acceptable for a new instrument [27]. The alpha computed for each of the four subscales exceeded the minimum value for a new tool. Cronbach's alphas for each factor were 0.88, 0.88, 0.82, and 0.86, respectively.

*1.2.5.4 Comparison between patients and caregivers*

Intraclass correlations (ICC) were computed to measure interrater reliability. All FaRE factor correlations were between 0.44 and 0.53, indicating an overall weak to moderate interrater reliability[28], suggesting that there is variation between patients and caregivers in rating the family resilience constructs. Perceived family coping was significantly different between patients and caregivers, the former perceiving higher levels of family resources (median score patients=6.3; median score caregivers=6.0; $p$=0.048). A comparison was carried out also within patient-caregiver dyads and the difference was not significant for any factor (see Supplementary Material).

*1.2.5.5 Comparison between cancer patients' populations*



For peer review only - http://bmjopen.bmj.com/site/about/guidelines.xhtml

Perceived social support was significantly different between breast and prostate cancer patients. The former reported receiving greater support from family and friends for their disease compared to the latter (*p*=0.002). Table 2 details the results of the non-parametric tests.

Table 2. Non-parametric tests on FaRe factors

| | **Patients (n=104)** | | | | **CG (n=105)** | | | | |
|---|---|---|---|---|---|---|---|---|---|
| **Factors** | **Median** | **Stdev** | **Q1** | **Q3** | **Median** | **Stdev** | **Q1** | **Q3** | **P-values** |
| Communication and cohesion | 6.6 | 0.9 | 5.7 | 7.5 | 6.5 | 0.8 | 5.7 | 7.3 | 0.238 |
| Perceived social support | 5.9 | 1.2 | 4.7 | 7.0 | 5.8 | 1.1 | 4.6 | 6.9 | 0.542 |
| Perceived family coping | 6.3 | 0.8 | 5.4 | 7.1 | 6.0 | 1.0 | 5.0 | 7.0 | **0.048** |
| Religiousness and Spirituality | 4.5 | 1.8 | 2.7 | 6.3 | 4.8 | 1.9 | 2.9 | 6.6 | 0.341 |

| | **Breast (n=53)** | | | | **Prostate (n=51)** | | | | |
|---|---|---|---|---|---|---|---|---|---|
| **Factors** | **Median** | **Stdev** | **Q1** | **Q3** | **Median** | **Stdev** | **Q1** | **Q3** | **P-values** |
| Communication and cohesion | 6.6 | 0.7 | 5.9 | 7.4 | 6.8 | 1.1 | 5.7 | 7.8 | 0.180 |
| Perceived social support | **6.3** | **0.8** | **5.4** | **7.1** | **5.5** | **1.3** | **4.2** | **6.8** | **0.002** |
| Perceived family coping | 6.0 | 0.9 | 5.1 | 6.9 | 6.5 | 0.7 | 5.8 | 7.2 | 0.171 |
| Religiousness and Spirituality | 4.8 | 1.8 | 3.0 | 6.5 | 4.3 | 1.9 | 2.3 | 6.2 | 0.285 |

P-values from Wilcoxon rank tests. Stdev: Standard deviation. Q1 and Q3 are the first and third quartiles.

### *1.2.6 Final version of questionnaire*

The final factors of the family resilience questionnaire are four. Communication and cohesion (items 1, 4, 7, 10, 13, 16, 19, 22) refers to the family's openness in communicating about the illness and its impact on their daily life. It also involves shared problem-solving and decision-making, resolution of conflict, openness regarding the range of feelings they are experiencing. The second factor, Perceived social support (items 2, 5, 8, 11, 14, 17, 20, 23), refers to mutual support, reaching out to extended kin and community for help with practical and emotional tasks. The third factor, Perceived family coping (items 3, 9, 15, 21), includes the ability to activate coping strategies and develop inner strength to deal with illness management. The concept of bouncing back and the ability to rebound from this stressful life event is present in this subscale. The last factor, Religiousness and spirituality (items 6, 12, 18, 24), refers



For peer review only - http://bmjopen.bmj.com/site/about/guidelines.xhtml

to spiritual values, transcendent beliefs and congregational support, which helps some families in finding meaning and purpose to the cancer diagnosis.

The final questionnaire was translated from Italian to English from a bilingual mother tongue and then back-translated by a professional translation agency for use in an English hospital. The questionnaire was evaluated by 10 healthy volunteers (friends and family members) to improve understanding of the English version. Four items were reworded accordingly.

## Material and Methods

### 2. Study 2: Validation of the FaRE questionnaire

The second study was designed to validate the factorial structure of the questionnaire in a new sample of breast cancer patients in two different phases of their therapeutic pathway: pre-admission phase (either for day-surgery admission or for ordinary surgery admission) and treatment phase (surgery, radiation therapy, or chemotherapy). In addition to this, the external stage of validation was conducted by assessing construct validity with some of the RSA and FaRE subscales. We expected to see positive correlations between: i) RSA and FaRE total scores; ii) RSA Family Cohesion subscale and FaRE Communication and Cohesion subscale; iii) RSA Social Resources and FaRE Perceived social support. While total scores should correlate as some individual resources could reflect in family resilience, the other two correlations were expected as they had common underpinning psychological constructs. Finally, significant correlations were hypothesized between RSA Perception of Self and FaRE Family coping, as the RSA factor incorporates positive outlook, confidence and self-efficacy, aspects which are present in the systemic view of the Family coping subscale.

*2.1 Participants*



For peer review only - http://bmjopen.bmj.com/site/about/guidelines.xhtml

A total of 143 couples was approached to explain the study, 34 couples refused due to time constraints between appointments or due to little interest in participating. Only breast cancer patients were recruited at this time due to easier access to patient waiting rooms, higher number of accesses in the hospital and higher compliance.

109 breast patients from a comprehensive cancer center in northern Italy and 104 family caregivers were recruited for the study. Four caregivers were not able to come to the consultation on the scheduled day and thus could not provide informed consent; their responses therefore were not considered for the data analysis. The procedure of recruitment was the same as for the first study.

*2.2 Materials*

The final version of the FaRE obtained at the end of Study 1, which consisted of 24 items, was administered to patients and caregivers (see Supplementary Material). As in Study 1, the RSA scale [22] was chosen to assess the convergent validity of the FaRE.

*2.3 Statistical analyses*

SAS software version 9.2 was used to conduct all statistical analyses. Confirmative Factorial Analysis (CFA) was conducted on the final version of the questionnaire. Wilcoxon signed rank tests were conducted to evaluate possible differences in clinical and sociodemographic characteristics between the two samples.

In order to assess construct validity of FaRE, we analyzed whether the subscales of the questionnaire correlated with the RSA using Spearman correlations. We chose a non-parametric test as our data was not normally distributed.

*2.4 Results*

*2.4.1 Descriptive statistics*



For peer review only - http://bmjopen.bmj.com/site/about/guidelines.xhtml

The median age of the population was 49 and IQR from 43 to 56. Thirty-three percent of the caregivers were female. Most participants were married or in a relationship (80% and 74% for patients and caregivers respectively) and had children (95% and 92% for patients and caregivers respectively). The majority of the participants had a high school degree or higher (82% for patients, 82% for caregivers). More than half of the patients were not receiving oncological therapy at time of recruitment and were diagnosed with Stage I-II breast cancer. Further descriptive information can be found in Table 3.

Table 3. Descriptive statistics of the second sample

| | | Overall (N=213) | Patients (n=109) | Caregivers (n=104) |
|---|---|---|---|---|
| Age | <50, n (%) | 116 (54) | 109 (51) | 104 (49) |
| | ≥50, n (%) | 97 (46) | 60 (55) | 56 (54) |
| Children | 0, n (%) | 47 (22) | 49 (45) | 48 (46) |
| | 1-2, n (%) | 153 (72) | 22 (20) | 25 (24) |
| | >3, n (%) | 13 (6) | 82 (75) | 71 (68) |
| Marital status | Single*, n (%) | 44 (21) | 20 (18) | 24 (23) |
| | Married, n (%) | 164 (77) | 87 (80) | 77 (74) |
| | Missing, n (%) | 5 (2) | 2 (2) | 3 (3) |
| Educational level | Elementary-middle school, n (%) | 38 (18) | 20 (18) | 18 (17) |
| | High school, n (%) | 92 (43) | 41 (38) | 51 (49) |
| | University, n (%) | 82 (38) | 48 (44) | 34 (33) |
| | Missing, n (%) | 1 (0) | 0 (0) | 1 (1) |
| Stage | 0, n (%) | | 11 (10.1) | |
| | I, n (%) | | 52 (47.7) | |
| | II, n (%) | | 25 (22.9) | |
| | III, n (%) | | 20 (18.8) | |
| Previous therapy | Surgery, n (%) | | 42 (38.5) | |
| | Chemotherapy, n (%) | | 19 (17.4) | |
| | Hormone therapy, n (%) | | 2 (1.8) | |
| | No therapy, n (%) | | 46 (42.2) | |
| Ongoing therapy | Chemotherapy, n (%) | | 16 (14.7) | |
| | Hormone therapy, n (%) | | 13 (11.9) | |
| | Radiotherapy, n (%) | | 10 (9.2) | |
| | No therapy, n (%) | | 70 (64.2) | |

*Single: never married or widow or separated

*2.4.2 Confirmatory factorial analysis*



For peer review only - http://bmjopen.bmj.com/site/about/guidelines.xhtml

The Bentler Comparative Fit Index was 0.90, the RMR Estimate 0.07, indicating an adequate model of fit between the model and the data.

*2.4.3 Construct validity*

Total scores of FaRE and RSA were positively correlated (ρs= 0.43, *p*<0.0001). Factor Communication and cohesion correlated positively with factor Family Cohesion in RSA (ρs= 0.56, *p*<0.0001). Factor Perceived social support correlated positively with factor Social Resources (ρs= 0.54, *p*<0.0001). Factor Perceived family coping correlated positively with factor Structured Style (ρs= 0.30, *p*<0.0001) and with factor Perception of Self in RSA (ρs= 0.42, *p*<0.0001).

*2.4.5 Concordance between patients' and caregivers' scores*

In this paired analysis, only scores of patients for which a corresponding caregiver's score was available were included. Differential scores were computed for each factor by subtracting the score of the patient from that of the corresponding caregiver. Paired t-tests indicated that the difference for Factor Communication and Cohesion was significantly different from zero (mean difference= -0.27, standard deviation= 0.93, *p*=0.003). No other significant differences were detected (see Supplementary Material).

*2.4.6 Differences between Study 1 and Study 2 samples*

Wilcoxon signed rank tests indicated no differences between the median values of factors in sample in Study 1 and the sample in Study 2, both when considering together the patients and the caregivers, as well as when the patients and the caregivers were compared separately.

*2.4.7 Normative family resilience score*

Due to disparity in number between prostate and breast cancer patients, normative data was calculated on the total breast cancer patients recruited for the two studies (N=317). Mean (5.7) and standard deviation (0.78) for for the total FaRE score was calculated. As with other clinical measures, scores



For peer review only - http://bmjopen.bmj.com/site/about/guidelines.xhtml

between -/+ 1 SD can be considered in the normal range, scores between -1 and -2 SD could indicate lower levels of family resilience and scores under 2 SD could be considered clinically relevant. For the last two ranges of scores referral to support services would be recommended.

## 3. Discussion

The FaRE questionnaire is designed to measure family resilience in oncological settings. Adopting a systemic approach, it considers both the patient and the caregiver's strengths and resources in managing the disease. Our initial version of the questionnaire had the same six family resilience constructs addressed by Sixbey[15]; however, a six-factor solution was not supported and it was reduced to four factors. Compared to Sixbey's[15] questionnaire, the FaRE does not include "Making meaning of adversity" and "Positive outlook"; however, the latter is addressed in the Perceived family coping subscale of the FaRE. Sixbey's Connectedness construct is incorporated in Family Communication and Cohesion. While Lane's [29] family resilience measure had highlighted that socio-economic resources had little value to breast cancer survivors; our measure suggests that social resources provide an invaluable support to the family coping with cancer and that economic resources were unrelated to the social ones. The development of the current questionnaire confirms that different resources are activated when families face cancer compared to other adversities and/or the general population. .

An important aspect to keep in mind is the low inter-rater reliability between patients and caregivers and this is partly explained by the significant differences found between some FaRE factors. Patients perceived significantly higher levels of family coping and higher levels of social support compared to their caregivers; although the difference in scores was small, it could be explained by the re-direction and allocation of shared coping strategies towards the patient rather than towards the caregiver. The



For peer review only - http://bmjopen.bmj.com/site/about/guidelines.xhtml

analysis on the patient-caregiver dyads of the second study also showed that caregivers reported significantly lower levels of open communication and emotional sharing compared to the corresponding patient. These results suggest that it is vital to consider the pluralistic view of family members as they are actively involved in cancer management. Further research should try and disentangle whether lower support and family coping scores of caregivers are of clinical significance, by assessing the association with mood profiles for instance..

Recruitment of participants from eight different regions of Italy and the combination of cognitive interviews and expert review, which allowed us to determine the relevance of the items, are some of the strengths of the study. Another strength of the developed measure is its systemic view, which allows use in caregiver populations, for whom there are only a few questionnaires available, possibly because attention towards their challenges and needs has increased only in the last few years [30,31].

This said there were some limitations in the development of the current questionnaire. The sample size of the first study is relatively small considering the number of items of the first version of the FaRE; a larger sample could have highlighted missing aspects. Convergent and discriminant validity examining similarities and differences between the FaRE and other family resilience tools would strengthen the rigor of the questionnaire. In addition to this, the cut-off points of the total FaRE scores need validating in future studies in order to use them as screening for referral. To ensure generalisability of the results, a larger sample with diverse tumour diagnosis should be recruited, as our sample was composed of only gender-related tumours with a relatively high life expectancy; in addition to this patients with other chronic and acute illnesses should be sampled to evaluate whether important family resilience aspects in disease management have been dropped. This instrument can be a useful tool to assess patients at baseline and for continued follow-up, for this reason test-retest reliability and responsiveness to change should be conducted



For peer review only - http://bmjopen.bmj.com/site/about/guidelines.xhtml

Preliminary evidence shows that the FaRE is a reliable and valid systemic measure of family resilience in oncological settings. The tool was designed to be implemented in a web platform as a part of the works of an European project, which aimed at promoting cancer patient's empowerment and self-management [32–34]. As increasing attention is being paid to family resilience and its protective role in illness trajectories, Once further validations are conducted, it can serve as a useful screening tool to identify patients and carers with few resources to deal with cancer related issues. Family resilience screening would allow targeted interventions on development and promotion of specific strategies and resources that can help the family deal with the illness. .

For peer review

**Funding**

This project has received funding from the European Union's Horizon 2020 research and innovation programme under grant agreement No 643529.

**Disclaimer**

This presentation reflects the authors' view. The Commission is not responsible for any use that may be made of the information it contains.

**Declaration of interest**

We have read and understood the BMJ Group policy on declaration of interests and we declare no conflict of interest.

**Author Contributions**

FF has made substantial contributions to article conception and design, was involved in recruitment of patients and in data entry, has given final approval of the version to be published. CR has undertaken the literature review to construct the measure, has developed the items of the questionnaire, has made



For peer review only - http://bmjopen.bmj.com/site/about/guidelines.xhtml

substantial contributions to article design, has been involved in drafting the manuscript conception the article, has given final approval of the version to be published. CF has undertaken the literature review to construct the measure, has developed the items of the questionnaire, has given final approval of the version to be published. CC was involved in recruitment of patients, data entry, has given final approval of the version to be published. SG has contributed to the analysis and interpretation of data for the work, to drafting the work and provided a critical revision for important intellectual content. GP has revised the article critically for important intellectual content, has given final approval of the version to be published.

**Data sharing statement**

Data leading to the results presented in this manuscript are available upon reasonable request from Sara Gandini: sara.gandini@ieo.it

**Acknowledgements**

We thank patients for providing feedback and suggestions for improving the family resilience tool.

**Figure legends**

Figure 1. Eingenvalues and proportion of variance of the factors

For peer review only

## References


1. Rutter M. Implications of resilience concepts for scientific understanding. *Ann N Y Acad Sci*. 2006;1094:1-12. doi:10.1196/annals.1376.002

2. Masten AS. Ordinary magic: Resilience processes in development. *Am Psychol*. 2001;56(3):227-238. doi:10.1037//0003-066X.56.3.227

3. Windle G. What is resilience? A review and concept analysis. *Rev Clin Gerontol*. 2011;21(2):152-169. doi:10.1017/S0959259810000420

4. Strauss B, Brix C, Fischer S, et al. The influence of resilience on fatigue in cancer patients undergoing radiation therapy (RT). *J Cancer Res Clin Oncol*. 2007;133(8):511-518. doi:10.1007/s00432-007-0195-z

5. Wenzel LB, Donnelly JP, Fowler JM, et al. Resilience, reflection, and residual stress in ovarian cancer survivorship: A gynecologic oncology group study. *Psychooncology*. 2002;11(2):142-153. doi:10.1002/pon.567

6. Min JA, Yoon S, Lee CU, et al. Psychological resilience contributes to low emotional distress in




For peer review only - http://bmjopen.bmj.com/site/about/guidelines.xhtml

cancer patients. *Support Care Cancer*. 2013;21(9):2469-2476. doi:10.1007/s00520-013-1807-6

7. Von Bertalanffy L. General system theory. In: *The Science of Synthesis: Exploring the Social Implications of General Systems Theory*. Boulder, Colorado: University Press of Colorado; 2003:304.

8. Van Schoors M, Caes L, Verhofstadt LL, Goubert L, Alderfer MA. Systematic review: Family resilience after pediatric cancer diagnosis. *J Pediatr Psychol*. 2015;40(9):856-868. doi:10.1093/jpepsy/jsv055

9. Walsh F. Family Resilience: A Framework for Clinical Practice. *Fam Process*. 2003;42(I):1-18. doi:10.1111/famp.2003.42.issue-1

10. Faccio F, Renzi C, Giudice A V., Pravettoni G. Family Resilience in the Oncology Setting: Development of an Integrative Framework. *Front Psychol*. 2018;9(666). doi:10.3389/fpsyg.2018.00666

11. McCubbin M, McCubbin HI. Families coping with illness: The resiliency model of family stress, adjustment, and adaptation. *Fam Heal Illn Perspect coping Interv*. 1993:21-63.

12. McCubbin MA, McCubbin HI, Thompson AI. Family hardiness index (FHI). In: *Family Assessment: Resiliency, Coping and adaptation–Inventories for Research and Practice*. ; 1996:239-305.

13. Walsh F. Applying a Family Resilience Framework in Training, Practice, and Research: Mastering the Art of the Possible. *Fam Process*. 2016;55(4):616-632. doi:10.1111/famp.12260

14. Rolland JS, Walsh F. Facilitating family resilience with childhood illness and disability. *Curr Opin Pediatr*. 2006;18(5):527-538. doi:10.1097/01.mop.0000245354.83454.68



For peer review only

For peer review only - http://bmjopen.bmj.com/site/about/guidelines.xhtml

15. Sixbey MT. Development of the family resilience assessment scale to identify family resilience constructs. *Dr Diss Univ Florida*. 2005.

16. Isaacs SA, Roman NV, Savahl S, Sui X-C. Adapting and Validating the Family Resilience Assessment Scale in an Afrikaans Rural Community in South Africa. *Community Ment Health J*. 2017;0(0):0. doi:10.1007/s10597-017-0091-1

17. Duca DS. Family resilience and parental stress: the effects on marital relationship in the context of a child diagnosed with an autism spectrum disorder. *Ann AII Cuza Univ Psychol Ser*. 2015;24(1):71-91. http://repository.upenn.edu/edissertations_sp2%0Ahttp://repository.upenn.edu/edissertations_sp2/14.

18. Chew J, Haase AM. Psychometric properties of the Family Resilience Assessment Scale: A Singaporean perspective. *Epilepsy Behav*. 2016;61:112-119. doi:10.1016/j.yebeh.2016.05.015

19. Walsh F. *Strengthening Family Resilience*.; 2013. doi:10.1086/514469

20. Lane CD, Meszaros PS. Measuring Walsh ' s Family Resilience Framework : Reliability and Validity of the Family Resilience Assessment ( FRA ) Among Women With a History of Breast Cancer. *Marriage Fam Rev*. 2016:1-16.

21. Messick S. Validity of psychological assessment: validation of inferences from persons' responses and performances as scientific inquiry into score meaning. *ETS Res Rep Ser*. 1994;1994(2):i-28. doi:10.1002/j.2333-8504.1994.tb01618.x

22. Capanna C, Stratta P, Hjemdal O, Collazzoni A, Rossi A. The Italian validation study of the Resilience Scale for Adults (RSA). *Appl Psychol Bull*. 2015;272(63):16-24. http://search.ebscohost.com/login.aspx?direct=true&db=psyh&AN=2015-19177-



For peer review only - http://bmjopen.bmj.com/site/about/guidelines.xhtml

002&site=ehost-live&scope=site.

23. Fabrigar LR, Wegener DT, Maccallum RC, Strahan EJ. Evaluating the Use of Exploratory Factor Analysis in Psychological Research. *Psychol Methods*. 1999;4(3):272-299.

24. Hatcher RL, Barends A, Hansell J, Gutfreund MJ. Patients' and Therapists' Shared and Unique Views of the Therapeutic Alliance: An Investigation Using Confirmatory Factor Analysis in a Nested Design. *J Consult Clin Psychol*. 1995;63(4):636-643.

25. Cerny BA, Kaiser HF. A Study Of A Measure Of Sampling Adequacy For Factor-Analytic Correlation Matrices. *Multivariate Behav Res*. 1977;12(1):43-47. doi:10.1207/s15327906mbr1201_3

26. Kang Y, McNeish DM, Hancock GR. The Role of Measurement Quality on Practical Guidelines for Assessing Measurement and Structural Invariance. *Educ Psychol Meas*. 2016;76(4):533-561. doi:10.1177/0013164415603764

27. DeVon HA, Block ME, Moyle-Wright P, et al. A Psychometric Toolbox for Testing Validity and Reliability. *J Nurs Scholarsh*. 2007;39(2):155-164. doi:10.1111/j.1547-5069.2007.00161.x

28. Koo TK, Li MY. A Guideline of Selecting and Reporting Intraclass Correlation Coefficients for Reliability Research. *J Chiropr Med*. 2016;15(2):155-163. doi:10.1016/j.jcm.2016.02.012

29. Meszaros PS, Allen KR, Few-demo AL, Piercy FP, Savla JS. Tracing the Pink Ribbon : Development of a Family Resilience Measure Crystal Lynn Duncan Lane Dissertation submitted to the faculty of the Virginia Polytechnic Institute and State University in partial fulfillment of the requirements of the degree of Doct. 2011.

30. Kim Y, Baker F, Spillers RL, Wellisch DK. Psychological adjustment of cancer caregivers with multiple roles. *Psychooncology*. 2006;15(9):795-804. doi:10.1002/pon.1013



For peer review only - http://bmjopen.bmj.com/site/about/guidelines.xhtml

31. Sklenarova H, Krümpelmann A, Haun MW, et al. When do we need to care about the caregiver? Supportive care needs, anxiety, and depression among informal caregivers of patients with cancer and cancer survivors. *Cancer*. 2015;121(9):1513-1519. doi:10.1002/cncr.29223

32. Kazantzaki E, Koumakis L, Kondylakis H, et al. Current trends in Electronic Family Resilience Tools: Implementing a tool for the cancer domain. In: ; 2018:29-32. doi:10.1007/978-981-10-5122-7_8

33. Renzi C, Fioretti C, Mazzocco K, et al. Development of psycho-emotional monitoring tools within an eHealth platform to improve patient empowerment and self-management abilities. *Psychooncology*. 2016;25(3):195.

34. Faccio F, Renzi C, Crico C, et al. Development of an eHealth tool for cancer patients: monitoring psychoemotional aspects with the family resilience (FaRe) questionnaire. *Ecancermedicalscience*. 2018;12. doi:10.3332/ecancer.2018.852

For peer review only



For peer review only - http://bmjopen.bmj.com/site/about/guidelines.xhtml

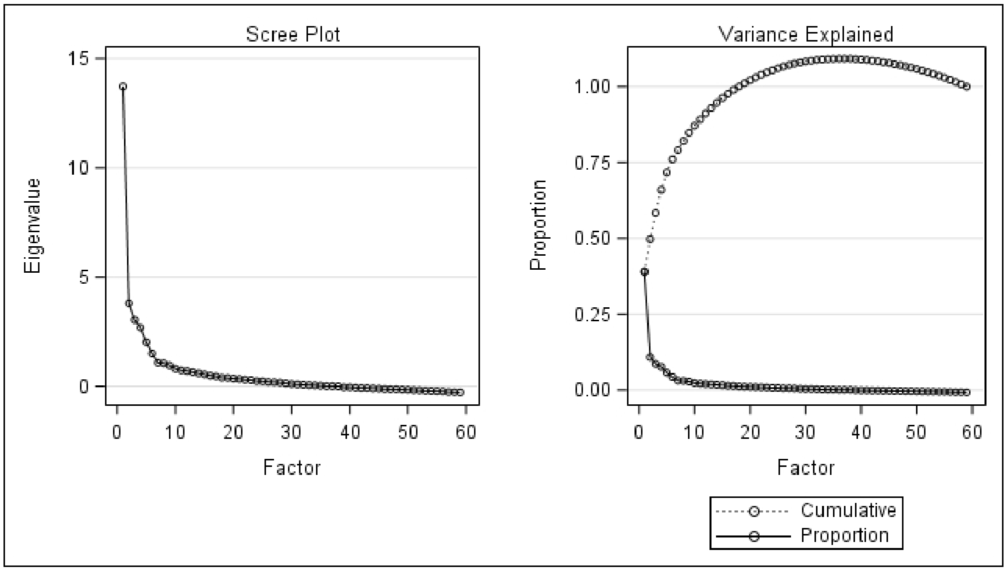


Figure 1. Eigenvalues and proportion of variance of the factors

**Supplementary Material**

Appendix A. *Family Resilience Questionnaire*

Dear Sir/Madam,

Please read each of the following statements carefully. For each one, state how well it describes your family. In thinking about your family, you can include all the significant people you think are part of it.

1. We understand each other with regard to the experience of illness we are living

Strongly disagree Moderately disagree Slightly disagree Nor Disagree nor agree Slightly Agree Moderately Agree Strongly agree

2. We ask our closest friends to help and assist us in this battle against the illness

Strongly disagree Moderately disagree Slightly disagree Nor Disagree nor agree Slightly Agree Moderately Agree Strongly agree

3. We believe that we can manage the illness

Strongly disagree Moderately disagree Slightly disagree Nor Disagree nor agree Slightly Agree Moderately Agree Strongly agree

4. In our family we feel that we can talk about how to communicate between us

Strongly disagree Moderately disagree Slightly disagree Nor Disagree nor agree Slightly Agree Moderately Agree Strongly agree

5. We can rely on our close friends to help us deal this illness

Strongly disagree Moderately disagree Slightly disagree Nor Disagree nor agree Slightly Agree Moderately Agree Strongly agree

For peer review only

6. We attend the church/synagogue/mosque/other places of worship

Strongly disagree Moderately disagree Slightly disagree Nor Disagree nor agree Slightly Agree Moderately Agree Strongly agree

7. We can deal this illness as a family

Strongly disagree Moderately disagree Slightly disagree Nor Disagree nor agree Slightly Agree Moderately Agree Strongly agree

8.We feel that the people in our social network would be happy to support us emotionally in dealing the illness

Strongly disagree Moderately disagree Slightly disagree Nor Disagree nor agree Slightly Agree Moderately Agree Strongly agree

9. We can solve important problems in our life such as this illness

Strongly disagree Moderately disagree Slightly disagree Nor Disagree nor agree Slightly Agree Moderately Agree Strongly agree

10. We discuss the illness-related problems until we find a shared solution

Strongly disagree Moderately disagree Slightly disagree Nor Disagree nor agree Slightly Agree Moderately Agree Strongly agree

11. We feels that our closest friends would be happy to support us emotionally in managing the illness

Strongly disagree Moderately disagree Slightly disagree Nor Disagree nor agree Slightly Agree Moderately Agree Strongly agree

12. We believe there is a supreme spiritual being that will help us deal this illness



For peer review only

Strongly disagree Moderately disagree Slightly disagree Nor Disagree nor agree Slightly Agree Moderately Agree Strongly agree

13. Everyone in the family feels free to express their own opinion regarding the illness

Strongly disagree Moderately disagree Slightly disagree Nor Disagree nor agree Slightly Agree Moderately Agree Strongly agree

14. We know that if we need comfort, our closest friends will be there for us

Strongly disagree Moderately disagree Slightly disagree Nor Disagree nor agree Slightly Agree Moderately Agree Strongly agree

15. We feel we are strong enough to cope with this illness

Strongly disagree Moderately disagree Slightly disagree Nor Disagree nor agree Slightly Agree Moderately Agree Strongly agree

16. We are honest when talking about the illness amongst ourselves

Strongly disagree Moderately disagree Slightly disagree Nor Disagree nor agree Slightly Agree Moderately Agree Strongly agree

17. We know we are important for our friends

Strongly disagree Moderately disagree Slightly disagree Nor Disagree nor agree Slightly Agree Moderately Agree Strongly agree

18. We participate in the activities of our religious community

Strongly disagree Moderately disagree Slightly disagree Nor Disagree nor agree Slightly Agree Moderately Agree Strongly agree



For peer review only

For peer review only - http://bmjopen.bmj.com/site/about/guidelines.xhtml

19. Everyone in the family is open to listening other's opinions regarding the illness

Strongly disagree Moderately disagree Slightly disagree Nor Disagree nor agree Slightly Agree Moderately Agree Strongly agree

20. We receive gifts and favours from our closest friends

Strongly disagree Moderately disagree Slightly disagree Nor Disagree nor agree Slightly Agree Moderately Agree Strongly agree

21. We have the strength to solve our problems

Strongly disagree Moderately disagree Slightly disagree Nor Disagree nor agree Slightly Agree Moderately Agree Strongly agree

22. The things we do for each other in dealing with this illness make us feel part of the family

Strongly disagree Moderately disagree Slightly disagree Nor Disagree nor agree Slightly Agree Moderately Agree Strongly agree

23. Our friends respect our family for how we reacted to the illness

Strongly disagree Moderately disagree Slightly disagree Nor Disagree nor agree Slightly Agree Moderately Agree Strongly agree

24. We ask our religious/spiritual reference figure for advice or words of comfort about the illness

Strongly disagree Moderately disagree Slightly disagree Nor Disagree nor agree Slightly Agree Moderately Agree Strongly agree



For peer review only - http://bmjopen.bmj.com/site/about/guidelines.xhtml

Appendix B. *Tables of biological descriptive statistics of Study 1 and differential scores in patient-caregiver dyads*

Table 1. Tumor characteristics of the first sample

| | | Total (n=104) | Breast (n=53) | prostate (n=51) |
|---|---|---|---|---|
| Lymph nodes | No | 80 (76.9) | 40 (75.5) | 40 (78.4) |
| | Yes | 22 (21.2) | 13 (24.5) | 9 (17.6) |
| | Missing | 2 (1.9) | 0 (0) | 2 (3.9) |
| Metastasis | No | 101 (97.1) | 53 (100) | 48 (94.1) |
| | Yes | 1 (1) | 0 (0) | 1 (2) |
| | Missing | 2 (1.9) | 0 (0) | 2 (3.9) |
| Stage | I | 15 (14.4) | 11 (20.8) | 4 (7.8) |
| | II | 52 (50) | 33 (62.3) | 19 (37.3) |
| | III-IV | 35 (33.7) | 9 (17) | 26 (51) |
| | Missing | 2 (1.9) | 0 (0) | 2 (3.9) |
| Grading | 1-2 | 39 (37.5) | 26 (49.1) | 13 (25.5) |
| | 3-4 | 64 (61.5) | 27 (50.9) | 37 (72.5) |
| | Missing | 1 (1) | 0 (0) | 1 (2) |
| Previous therapy | No | 28 (26.9) | 12 (22.6) | 16 (31.4) |
| | Yes | 75 (72.1) | 41 (77.4) | 34 (66.7) |
| | Missing | 1 (1) | 0 (0) | 1 (2) |

Table 2. Differences in family resilience scores in patient-caregiver dyads in the first study

| **Differences pt-CG** | **Median** | **Low** | **Up** |
|---|---|---|---|
| factor1_com | 0 | -0.38 | 0.5 |
| factor2_supp | 0 | -0.5 | 0.75 |
| factor3_coping | 0.13 | -0.38 | 1 |
| factor4_spirit | -0.25 | -1 | 1 |

Low: lower quartile; Up: upper quartile

Factor1_com = Communication and cohesion; Factor2_supp= Perceived social support; Factor3_coping = Perceived family coping; Factor4_spirit = Religiousness and Spirituality

Table 3. Mean differential scores and paired-t test between patient-caregiver dyads in the second study

| | Mean | SD | p-value |
|---|---|---|---|
| **diff_factor1** | **-0.27** | 0.93 | **0.003** |



For peer review only - http://bmjopen.bmj.com/site/about/guidelines.xhtml

| | | | |
|---|---|---|---|
| diff_factor2 | 0.21 | 1.17 | 0.074 |
| diff_factor3 | -0.14 | 1.05 | 0.179 |
| diff_factor4 | 0.27 | 1.72 | 0.111 |

Factor1 = Communication and cohesion; Factor2= Perceived social support; Factor3= Perceived family coping; Factor4 = Religiousness and Spirituality

Table 4. Eingenvalues and proportion of variance of the factors

| | Eigenvalue | Difference | Proportion | Cumulative |
|---|---|---|---|---|
| **1** | **13.72** | **9.92** | **0.39** | **0.39** |
| **2** | **3.81** | **0.77** | **0.11** | **0.50** |
| **3** | **3.04** | **0.35** | **0.09** | **0.58** |
| **4** | **2.69** | **0.66** | **0.08** | **0.66** |
| 5 | 2.02 | 0.52 | 0.06 | 0.72 |
| 6 | 1.51 | 0.42 | 0.04 | 0.76 |
| 7 | 1.08 | 0.03 | 0.03 | 0.79 |
| 8 | 1.06 | 0.09 | 0.03 | 0.82 |
| 9 | 0.96 | 0.15 | 0.03 | 0.85 |



For peer review only - http://bmjopen.bmj.com/site/about/guidelines.xhtml

BMJ Open

# Development and validation of a Family Resilience questionnaire (FaRE): an observational study in Italy


| | |
|---|---|
| Journal: | *BMJ Open* |
| Manuscript ID | bmjopen-2018-024670.R2 |
| Article Type: | Research |
| Date Submitted by the Author: | 14-Jan-2019 |


| | |
|---|---|
| Complete List of Authors: | Faccio, Flavia; Università degli Studi di Milano, Department of Oncology and Hemato-Oncology; Istituto Europeo di Oncologia, Applied Division for Cognitive and Psychological Science<br>Renzi, Chiara; European Institute of Oncology, European Institute of Oncology<br>Fioretti, Chiara; Università degli Studi di Firenze, Department of Educational Sciences and Psychology<br>Crico, Chiara; Università degli Studi di Milano, Department of Oncology and Hemato-Oncology; Istituto Europeo di Oncologia, Applied Division for Cognitive and Psychological Science<br>Gandini, Sara; European Institute of Oncology, Division of Epidemiology and Biostatistics<br>Pravettoni, Gabriella; Istituto Europeo di Oncologia, Applied Research Division for Cognitive and Psychological Science; Universita degli Studi di Milano, Department of Oncology and Hemato-oncology |


| | |
|---|---|
| <b>Primary Subject Heading</b>: | Patient-centred medicine |
| Secondary Subject Heading: | General practice / Family practice, Oncology, Research methods, Patient-centred medicine |
| Keywords: | Family Resilience, Stress and coping, Surveys and Questionnaires, Systems theory, ONCOLOGY, Family Adaptation |
| | |


SCHOLARONE™
Manuscripts

# Development and validation of a Family Resilience questionnaire (FaRE): an observational study in Italy

*Flavia Faccio[1,2‡], Chiara Renzi[1], Chiara Fioretti[3], Chiara Crico[1,2], Sara Gandini[4], Gabriella Pravettoni[1,2]

[1] Applied Research Division for Cognitive and Psychological Science, IEO, European Institute of Oncology IRCCS, Milan, Italy

[2] Department of Oncology and Hemato-Oncology, University of Milan, Milan, Italy

[3]Department of Educational Sciences and Psychology, Università degli Studi di Firenze, Florence, Italy

[4]Division of Epidemiology and Biostatistics, IEO, European Institute of Oncology IRCCS, Milan, Italy

Email addresses flavia.faccio.90@gmail.com; chiara.renzi@ieo.it; chiara.fioretti@unifi.it; chiara.crico@ieo.it; sara.gandini@ieo.it; gabriella.pravettoni@ieo.it

Word count: 4601

‡Corresponding author:

Flavia Faccio, flavia.faccio.90@gmail.com, Telephone: +39 029437099, Applied Research Unit for Cognitive and Psychological Science, European Institute of Oncology, Milan, Via Ripamonti 435.



For peer review only

**Abstract**

*Objective:* Develop and validate an instrument to assess family resilience and, more specifically, the family dynamics and resources, estimating the adaptation flexibility to cancer disease. Cohesion, communication, coping style, and relational style were considered as critical functional areas in the construction of the instrument.

*Design*: Two cross-sectional studies. Study 1: Identification of factorial structure of the questionnaire in two samples with different cancer sites. Study 2: Validation of the questionnaire in cancer patients in two different phases of their therapeutic pathway.

*Participants and setting:* A total of 213 patients with a histologically confirmed non-metastatic breast or prostate cancer and 209 caregivers were recruited for the two studies from an oncological hospital in Italy.

*Outcome measures*: The Resilience Scale for Adults and the Family Resilience questionnaire (FaRE) developed by the researchers were administered to all patients and caregivers that gave consent.

*Results:* In Study 1, the 60-item version of the FaRE underwent discriminant and construct validity, internal consistency, and factorial analysis. Comparisons between patient and caregiver populations showed that patients perceived higher levels of family resources ($p$=0.048) and that prostate cancer patients perceive less social support compared to breast cancer ($p$=0.002). Factor analysis demonstrated four domains: Communication and cohesion, Perceived social support, Perceived family coping, and Religiousness and spirituality. In Study 2 the validity and factorial structure of the final scale, composed of 24 items, were confirmed. The Cronbach's alpha of all subscales was above .82. Normative values for breast cancer patients can provide indications of family resilience levels.

*Conclusions:* Preliminary findings showed acceptable psychometric properties for the FaRE to evaluate family resilience in oncological patients and their caregivers. Further research should test



For peer review only - http://bmjopen.bmj.com/site/about/guidelines.xhtml

its sensibility to change to assess its use as a psycho-emotional monitoring too and its validity in other medical contexts.

**Keywords**

Family Resilience, Family Adaptation, Stress and coping, Surveys and Questionnaires, Systems Theory, Oncology

**Article summary**

***Strengths and limitations of this study***

- Questionnaire development and testing was conducted in two phases in order to assess validity and reliability of the final version of the measure.
- This is the first measure of family resilience that adopts a systemic approach, considering both the patient and caregiver, in light of increasing evidence on the vital role the caregiver.
- Results were controlled for the possible confounding sociodemographic variables.
- Only two oncological populations were recruited, making generalisability to all types of tumour patients limited.
- Longitudinal measurements are advised to investigate test-retest reliability and responsiveness to change of the measure.

For peer review only

## Introduction

Resilience is a multifaceted concept that can be described as the ability to mobilize resources and adapt to challenging or adverse situations [1,2]. One of the internal resources of resilience is coping, which consists of a set of modifiable skills that can be learnt to deal with a stressful life event[3]. More and more often, resilience is studied as a relevant aspect when facing a disease such as cancer [4–6]. However, the impact of disrupting events such as a cancer diagnosis, is not limited only to the individual, rather it influences also their family and social network, which can initiate or support positive adaptation as well as be overwhelmed by the demands of the disease and its treatments. According to the family systems theory [7], a change or a perturbation occurring to one member of the system will affect also the other members, which may result in a smooth adaptation to a new homeostasis or in difficulties that prevent the readjustment process [7]. This translates into clinically significant levels of distress, higher risk of developing psychosocial problems, high levels of conflict and low family cohesion[8]. Going beyond the idea that families which encounter difficulties are "damaged" or "pathological" [9], resilience may represent a pivotal concept to analyse and support the family in moving towards a new balance and in constructing meaning of the cancer event [10].

A substantial contribution to research in family resilience comes from two main models: the Resiliency Model of Family Adjustment and Adaptation[11] and the Family Resilience Framework[9], both of which have been applied to oncological settings. Walsh's framework was chosen for various reasons: first, conceptualisation of family resilience in McCubbin's model is quite rigid, it belongs to a first wave of resilience research that believed one has to progress through specific steps in order to achieve resilience. In addition to this, due to the large number of variables involved in the model, it was possible for previous studies to validate only parts of it, never in its entirety[12]. Differently, Walsh [9,13] defines resilience as the family's ability to "withstand and rebound from adversity, strengthened and more resourceful", including the capacity to cope and adapt to the stressor and the concept of post-traumatic growth. She considers three major dimensions as contributing to the system's



For peer review only - http://bmjopen.bmj.com/site/about/guidelines.xhtml

resilience: i) belief systems, ii) organizational patterns and iii) communication processes. Each of these overarching constructs is composed of three subprocesses: Make meaning of adversity, Positive outlook, Transcendence and spirituality, Flexibility, Connectedness, Social and economic resources, Communication/problem-solving, Clarity, Open emotional expression, Collaborative problem-solving. These are considered mutually interactive and synergistic as they facilitate and sustain each other across systems and over time [13]. Walsh also proposed an integrative family resilience model for illness related challenges, including cancer[14]; currently, there is no quantitative evidence regarding the framework's efficacy.

Only three questionnaires have attempted to capture the multiple dimensions of Walsh's[9] framework. Sixbey [15] developed the Family Resilience Assessment Scale (FRAS), a 66-item measure with responses given on a 4-point Likert scale. The Statistical considerations and factor loadings showed that a nine construct model of family resilience was not supported and therefore the number of factors was reduced to six [15]: i) family communication and problem-solving, ii) social and economic resources, iii) maintaining a positive outlook, iv) family connectedness, v) family spirituality and vi) making meaning of adversity. One of the subscales, Communication and Problem-solving, is an overarching construct of Walsh's model, which originally contained three sub-processes The questionnaire was administered to the general American population without considering an essential aspect of resilience, namely whether an adversity had occurred. A second validation study suggested that the FRAS could be a reliable and valid measure in a population which was not experiencing an adversity [16]. This is the major weakness of the measure, which has then been administered to families that have experienced difficult diagnoses, such as autism spectrum disorder [17] and epilepsy [18], where disagreement regarding the number of factors of the scale is highlighted[18]. In addition to this, the measure was never administered to multiple members of a family, therefore the perspective of the questionnaire is individualistic and is not representative of the family unit as a whole. As individual



For peer review only - http://bmjopen.bmj.com/site/about/guidelines.xhtml

perceptions of which resilience resources are activated may vary, it is essential to compare the views of different family members[19].

The other two measures developed according to Walsh's framework are the Family Resilience Assessment (FRA; Lane & Meszaros, 2016) and the Walsh Family Resilience Questionnaire (WFRQ; Walsh, 2013). The former is composed of 29 items on a 5-point scale response and was validated in a sample of women with a history of breast cancer. The developers [20] warned that the items did not always reflect Walsh's indicators, decided to group the items in the three overarching dimensions and suggested that socio-economic resources have little value in breast cancer survivors. Lane implies that further studies should capture the pluralistic view of the family members[20]. To date the FRA has not been used in other oncological or more general health contexts, suggesting the need for further studies in order to consider the instrument a valid measure. In addition to this, the recruited sample was small and different subgroups were present: survivors who had received diagnosis in the past 15 years, others in the past 5 years, another group of women undergoing treatment, of which half has a stage IV breast cancer diagnosis. Therefore, both current and retrospective accounts of resilience were collected. Finally, Walsh [19] developed her own self-report questionnaire, composed of 33 items that operationalise her nine key processes and provide a map of each family. While she states that her questionnaire is being applied in several international projects, data from these studies has not been published yet.

As there is only one measure of family resilience in cancer patients [15] which has an individualistic view of resilience and does not capture the systemic processes involved, a new measure including Sixbey's six factors was developed. These factors were chosen compared to Lane's three factors as, in our opinion, not all salient aspects of family resilience in cancer had been considered and to avoid construct underrepresentation, one of the major threats to construct validity[21]. The aim of this measure is to investigate family resources and strengths during cancer management, taking into account both the patient's and the caregiver's perspective.



For peer review only - http://bmjopen.bmj.com/site/about/guidelines.xhtml

## Material and methods

All study phases were presented in a single protocol to the local Ethics Committee, which was approved in all its parts on the 11th of April, 2016. The aim of the first study was to develop a questionnaire, the Family Resilience Evaluation Questionnaire (FaRE) to assess resilience in families affected by cancer. Following Messick's[21] guidelines for validating a measure, the following stages were conducted: substantive, structural, and external. While substantive and structural steps were undertaken in Study 1, the external stage is present in Study 2.

### 1. Study 1: Item generation phase and development of the questionnaire

For the substantive stage, a first version of the FaRE was constructed by the authors after a thorough revision of scientific literature in the field of resilience and cancer and after conducting informal, clinical appointments with 20 cancer patients about strategies that helped them adjust and cope with the illness. The questionnaire was then developed and assessed by an expert panel composed of psychologists that conduct research in the field of resilience. Some minor adjustments to the questions was made and five items were dropped as they were considered too similar to other statements. Finally, this preliminary version of the FaRE was administered to a pilot group of 10 citizens, 10 cancer patients and 10 caregivers comparable to the target population in order to evaluate item comprehensibility, provide suggestions and indicate which items seemed unclear. Six items were re-worded to improve understanding. The second version of the questionnaire was composed of 60 items referring to six aspects: 1) family communication and problem solving, 2) social and economic resources, 3) maintaining a positive outlook, 4) family connectedness, 5) family spirituality, and 6) ability to make meaning from adversity. Questions were on a seven-point Likert scale (from Strongly Agree to Strongly Disagree).



For peer review only - http://bmjopen.bmj.com/site/about/guidelines.xhtml

## 1.2 Item selection phase

*1.2.1 Participants*

Couples of patients and caregivers that fitted the inclusion criteria were asked to participate in the current study. Participation was proposed to a total of 146 couples, 42 of which refused due to little interest in the study or lack of time. 105 patients (53 breast, 52 prostate cancer patients) and 105 caregivers (i.e., son/daughter, wife/husband/partner, father/mother, sister/brother) were recruited for the study at a comprehensive cancer centre in northern Italy. Patients were recruited from two outpatient clinics, one of the Breast Cancer Division, the second of the Urology Division. In addition to this, patients receiving oncological treatment were recruited from the Radiotherapy Division and Day Hospital. If compatible with the researcher's availability, consecutive patients who were waiting for their appointment were enrolled in the study. Once the study's aims and procedures were illustrated, all participants gave written informed consent.

Inclusion criteria for patients were the following: 1) between 25 and 80 years of age; 2) non-metastatic cancer diagnosis of breast or prostate cancer; 4) no psychiatric, addictive, or cognitive disorders that would prevent compliance with protocol requirements.

The inclusion criteria for caregivers were: 1) over 18 years old; 2) had a relative with a diagnosis of breast or prostate cancer; 3) no psychiatric, addictive, or cognitive disorders that would prevent compliance with protocol requirements.

Due to incomplete answers of one patient his data was not included in the database, therefore the final sample was composed by 104 patients and 105 caregivers.

*1.2.2 Materials*

The revised version of the FaRE, obtained at the end of the pilot study, was composed of 60 items and it was administered to patients and caregivers participating to the study.



For peer review only - http://bmjopen.bmj.com/site/about/guidelines.xhtml

The Italian version of the Resilience Scale for Adults (RSA) [22] was chosen to assess the convergent validity of the FaRE. The RSA is a 33 item self-report scale for adults that measures six resilience factors: Perception of Self, Planned Future, Social Competence, Structured Style, Family Cohesion, and Social Resources. It provides a total mean score, with higher scores indicating higher resilience. Four factors measure the individual's characteristics, one the family environment and the last factor social networks. Cronbach's alphas for the Italian version ranged from 0.75 to 0.90 for all subscales and total score with the exception of Structured Style (0.34), which showed poor reliability. *1.2.3 Patient involvement and procedure*

The patients in this study were participants. They were not involved in the development of the research question or in the recruitment phase. However, patients were asked for feedback on the readability of the questionnaire in the pilot phase and their suggestions were incorporated in the final version of the FaRE.

Participants were approached by the researcher while they were in the waiting hall for the appointment with a healthcare professional (oncologist, surgeon, or nurse). The researcher explained the purposes of the study and asked both the patient and the caregiver if they were interested in participating. If they answered affirmatively, an appointment was made after their medical consultation, during which further details were given, together with an informed consent that was signed by both the researcher and the participants. The questionnaires were administered on an iPad; for those who did not use electronic devices paper-and-pencil versions of the questionnaires were given.

*1.2.4 Statistical analyses*

All statistical analyses were conducted using SAS (Statistical Analysis System) version 9.2. The first step was an exploratory factor analysis (EFA) to reduce the item pool [23]. The EFA was conducted using maximum likelihood factoring with orthomax rotation. Several criteria provided guidelines in selecting the number of factors to extract [24]. Factors with eigenvalues greater than 1.0 were retained



For peer review only - http://bmjopen.bmj.com/site/about/guidelines.xhtml

and an examination of scree plots of eigenvalues and total variance explained by retained factors helped to determine the total number of factors to retain (see Figure 1).More generally, in the early stages and throughout the entire process, the items retained were those with the highest explained variance for each factor and those that belonged together.

A Cronbach coefficient alpha was calculated for the entire scale and for each individual subscale to assure internal consistency and reliability.

To evaluate the goodness of fit between the models and data we used fit indices, including the Bentler comparative fit index (CFI) and the RMSEA that indicates the amount of unexplained variance.

Total and subscale FaRE scores were compared between patients and caregivers, age groups, levels of education, marital status, and number of children by Wilcoxon rank tests. We did not expect differences between sociodemographic categories while we expected that differences could arise between patients and caregivers, as activation of resources can differ depending on the role of the family member in adjusting to the illness.

## *1.2.5 Results*

### *1.2.5.1 Descriptive statistics*

The median age of the population was 60 years old, with an interquartile range (IQR) from 51 to 66.

Sixty percent of caregivers were female. 71% of caregivers were wife/husband, 14% were son/daughter, 11% were sister/brother, and the remaining 4% were mother/father to the patient. The majority of the participants was married or in a relationship (83.7% and 81% for patients and caregivers respectively) and had children (84.6% and 76.2% for patients and caregivers respectively). Most participants had a high school degree (41.3% for patients, 44% for caregivers) or higher (33.7% for participants, 29% for caregivers). No sociodemographic variables were significantly different between groups. Further descriptive data can be found in Table 1.



For peer review only - http://bmjopen.bmj.com/site/about/guidelines.xhtml

Table 1. Sociodemographic characteristics of the sample

| | | Total (N=209) | Patients (N=104) | CG (N=105) | P-values |
|---|---|---|---|---|---|
| Age | <50, n (%) | 43 (20.6) | 22 (21.2) | 21 (20) | 0.84 |
| | ≥50, n (%) | 162 (77.5) | 80 (76.9) | 82 (78.1) | |
| | Missing, n (%) | 4 (1.9) | 2 (1.9) | 2 (1.9) | |
| Gender | Male, n (%) | 93 (44.5) | 51 (49) | 42 (40) | 0.19 |
| | Female, n (%) | 116 (55.5) | 53 (51) | 63 (60) | |
| Education | Elementary-middle, n (%) | 49 (23.4) | 23 (22.1) | 26 (24.8) | 0.76 |
| | High school, n (%) | 90 (43.1) | 43 (41.3) | 47 (44.8) | |
| | University, n (%) | 66 (31.6) | 35 (33.7) | 31 (29.5) | |
| | Missing, n (%) | 4 (1.9) | 3 (2.9) | 1 (1) | |
| Marital status | Single*, n (%) | 35 (16.7) | 17 (16.3) | 18 (17.1) | 0.83 |
| | Married, n (%) | 172 (82.3) | 87 (83.7) | 85 (81) | |
| | Missing, n (%) | 2 (1) | 0 (0) | 2 (1.9) | |
| Children | No, n (%) | 39 (18.7) | 16 (15.4) | 23 (21.9) | 0.36 |
| | 1-2, n (%) | 133 (63.6) | 68 (65.4) | 65 (61.9) | |
| | >2, n (%) | 35 (16.7) | 20 (19.2) | 15 (14.3) | |
| | Missing, n (%) | 2 (1) | 0 (0) | 2 (1.9) | |

CG: Caregiver; *Single: never married or widow or separated

Around 83% of breast and 45% of prostate cancer patients were diagnosed with Stage I-II, the remaining patients had stage III-IV tumours. All breast and most prostate patients had undergone at least one type of oncological therapy before (radiotherapy, chemotherapy and/or surgery). One prostate patient underwent PET scan for tumour stadiation several weeks after completing the questionnaires and the test indicated the presence of metastases. Further clinical data can be found in the Supplementary Material.

*1.2.5.2 Factorial analysis*

Kaiser Meyer Olkin's test for sampling adequacy was conducted on the sample to evaluate the adequacy of data. All values were above 0.8 (overall KMO= 0.92, patient sample KMO= 0.9, caregiver sample KMO=0.84), indicating that the data was suitable for factorial analysis[25].

Explained variance was over 10% for 4 factors, for the remaining two factors the explained variance was under 5%, which is considered too low to be retained and were therefore excluded. Factor 5 had



For peer review only - http://bmjopen.bmj.com/site/about/guidelines.xhtml

only two items, Factor 6 three and in both factors the items were unrelated to each other. EFA indicated 4 factors: one factor (Factor 5) of the initial version of the questionnaire was removed ("Ability to make meaning from adversity") and some items of two factors, namely "Family communication and problem solving" and "Family connectedness" were collapsed into a new subscale, Communication and Cohesion (see Supplementary material).

A sufficient model fit (Bentler CFI) was defined as of 0.90 or greater, the RMSEA of 0.06 or less. The Bentler CFI was 0.94 ($\chi^2$= 2571.16, DF=276, $p$<.001), the RMSEA Estimate 0.05, indicating an adequate fit between the model and the data.

For each factor, the items with the highest weights were selected. The number of items for factors Communication and cohesion and Perceived social support is eight, four items for Perceived family coping, Religiousness and spirituality.

*1.2.5.3 Final version of questionnaire*

The final factors of the family resilience questionnaire are four. Communication and cohesion (items 1, 4, 7, 10, 13, 16, 19, 22) refers to the family's openness in communicating about the illness and its impact on their daily life. It also involves shared problem-solving and decision-making, resolution of conflict, openness regarding the range of feelings they are experiencing. The second factor, Perceived social support (items 2, 5, 8, 11, 14, 17, 20, 23), refers to mutual support, reaching out to extended kin and community for help with practical and emotional tasks. The third factor, Perceived family coping (items 3, 9, 15, 21), includes the ability to activate coping strategies and develop inner strength to deal with illness management. The concept of bouncing back and the ability to rebound from this stressful life event is present in this subscale. The last factor, Religiousness and spirituality (items 6, 12, 18, 24), refers to spiritual values, transcendent beliefs and congregational support.

*1.2.5.4 Internal consistency*



For peer review only - http://bmjopen.bmj.com/site/about/guidelines.xhtml

An alpha of 0.70 is nowadays considered as acceptable for a new instrument [26]. The alpha computed for each of the four subscales exceeded the minimum value for a new tool. Cronbach's alphas for each factor were 0.88, 0.88, 0.82, and 0.86, respectively. To further assess internal reliability, additional indices were computed: Average Variance Extracted (AVE) values were 0.52, 0.48, 0.58, 0.71 and Joreskog rho values were 0.90, 0.88, 0.84, 0.91, respectively. As an AVE value of 0.5 or more indicates good reliability and a rho value of 0.70 indicates acceptable reliability it is possible to assume adequate internal consistency.

*1.2.5.5 Comparison between patients and caregivers*

Intraclass correlations (ICC) were computed to measure interrater reliability. All FaRE factor correlations were between 0.44 and 0.53, indicating an overall weak to moderate interrater reliability[27], suggesting that there is variation between patients and caregivers in rating the family resilience constructs. Perceived family coping was significantly different between patients and caregivers, the former perceiving higher levels of family resources (median score patients=6.3; median score caregivers=6.0; $p$=0.048). A comparison was carried out also within patient-caregiver dyads and the difference was not significant for any factor (see Supplementary Material).

*1.2.5.6 Comparison between cancer patients' populations*

Perceived social support was significantly different between breast and prostate cancer patients. The former reported receiving greater support from family and friends for their disease compared to the latter ($p$=0.002). Table 2 details the results of the non-parametric tests.

Table 2. Non-parametric tests on FaRe factors

| | **Patients (n=104)** | | | | **CG (n=105)** | | | | |
|---|---|---|---|---|---|---|---|---|---|
| **Factors** | **Median** | **Stdev** | **Q1** | **Q3** | **Median** | **Stdev** | **Q1** | **Q3** | **P-values** |
| Communication and cohesion | 6.6 | 0.9 | 5.7 | 7.5 | 6.5 | 0.8 | 5.7 | 7.3 | 0.238 |
| Perceived social support | 5.9 | 1.2 | 4.7 | 7.0 | 5.8 | 1.1 | 4.6 | 6.9 | 0.542 |
| Perceived family coping | 6.3 | 0.8 | 5.4 | 7.1 | 6.0 | 1.0 | 5.0 | 7.0 | **0.048** |
| Religiousness and Spirituality | 4.5 | 1.8 | 2.7 | 6.3 | 4.8 | 1.9 | 2.9 | 6.6 | 0.341 |



For peer review only - http://bmjopen.bmj.com/site/about/guidelines.xhtml

| | Breast (n=53) | | | | Prostate (n=51) | | | | |
|---|---|---|---|---|---|---|---|---|---|
| **Factors** | **Median** | **Stdev** | **Q1** | **Q3** | **Median** | **Stdev** | **Q1** | **Q3** | **P-values** |
| Communication and cohesion | 6.6 | 0.7 | 5.9 | 7.4 | 6.8 | 1.1 | 5.7 | 7.8 | 0.180 |
| Perceived social support | **6.3** | **0.8** | **5.4** | **7.1** | **5.5** | **1.3** | **4.2** | **6.8** | **0.002** |
| Perceived family coping | 6.0 | 0.9 | 5.1 | 6.9 | 6.5 | 0.7 | 5.8 | 7.2 | 0.171 |
| Religiousness and Spirituality | 4.8 | 1.8 | 3.0 | 6.5 | 4.3 | 1.9 | 2.3 | 6.2 | 0.285 |

P-values from Wilcoxon rank tests. Stdev: Standard deviation. Q1 and Q3 are the first and third quartiles.

# Material and Methods

## 2. Study 2: Validation of the FaRE questionnaire

The second study was designed to validate the factorial structure of the questionnaire in a new sample of breast cancer patients in two different phases of their therapeutic pathway: pre-admission phase (either for day-surgery admission or for ordinary surgery admission) and treatment phase (surgery, radiation therapy, or chemotherapy). In addition to this, the external stage of validation was conducted by assessing construct validity with some of the RSA and FaRE subscales. We expected to see positive correlations between: i) RSA and FaRE total scores; ii) RSA Family Cohesion subscale and FaRE Communication and Cohesion subscale; iii) RSA Social Resources and FaRE Perceived social support. While total scores should correlate as some individual resources could reflect in family resilience, the other two correlations were expected as they had common underpinning psychological constructs. Finally, significant correlations were hypothesized between RSA Perception of Self and FaRE Family coping, as the RSA factor incorporates positive outlook, confidence and self-efficacy, aspects which are present in the systemic view of the Family coping subscale.

*2.1 Participants*

A total of 143 couples was approached to explain the study, 34 couples refused due to time constraints between appointments or due to little interest in participating. Only breast cancer patients were recruited at this time due to easier access to patient waiting rooms, higher number of accesses in the hospital and higher compliance.



For peer review only - http://bmjopen.bmj.com/site/about/guidelines.xhtml

109 breast patients from a comprehensive cancer center in northern Italy and 104 family caregivers were recruited for the study. Four caregivers were not able to come to the consultation on the scheduled day and thus could not provide informed consent; their responses therefore were not considered for the data analysis. The procedure of recruitment was the same as for the first study.

*2.2 Materials*

The final version of the FaRE obtained at the end of Study 1, which consisted of 24 items, was administered to patients and caregivers (see Supplementary Material). As in Study 1, the RSA scale [22] was chosen to assess the convergent validity of the FaRE.

*2.3 Statistical analyses*

SAS software version 9.2 was used to conduct all statistical analyses. Confirmative Factorial Analysis (CFA) was conducted on the final version of the questionnaire. Multigroup factor analysis was conducted to test for weak and strong factorial invariance across patients and caregivers. Wilcoxon signed rank tests were conducted to evaluate possible differences in clinical and sociodemographic characteristics between the two samples.

In order to assess construct validity of FaRE, we analyzed whether the subscales of the questionnaire correlated with the RSA using Spearman correlations. We chose a non-parametric test as our data was not normally distributed.

*2.4 Results*

*2.4.1 Descriptive statistics*

The median age of the population was 49 and IQR from 43 to 56. Thirty-three percent of the caregivers were female. Most participants were married or in a relationship (80% and 74% for patients and caregivers respectively) and had children (95% and 92% for patients and caregivers respectively). The majority of the participants had a high school degree or higher (82% for patients, 82% for caregivers). More than half of the patients were not receiving oncological therapy at time of



For peer review only - http://bmjopen.bmj.com/site/about/guidelines.xhtml

For peer review only

recruitment and were diagnosed with Stage I-II breast cancer. Further descriptive information can be found in Table 3.

Table 3. Descriptive statistics of the second sample

| | | Overall (N=213) | Patients (n=109) | Caregivers (n=104) |
|---|---|---|---|---|
| Age | <50, n (%) | 116 (54) | 109 (51) | 104 (49) |
| | ≥50, n (%) | 97 (46) | 60 (55) | 56 (54) |
| Children | 0, n (%) | 47 (22) | 49 (45) | 48 (46) |
| | 1-2, n (%) | 153 (72) | 22 (20) | 25 (24) |
| | >3, n (%) | 13 (6) | 82 (75) | 71 (68) |
| Marital status | Single*, n (%) | 44 (21) | 20 (18) | 24 (23) |
| | Married, n (%) | 164 (77) | 87 (80) | 77 (74) |
| | Missing, n (%) | 5 (2) | 2 (2) | 3 (3) |
| Educational level | Elementary-middle school, n (%) | 38 (18) | 20 (18) | 18 (17) |
| | High school, n (%) | 92 (43) | 41 (38) | 51 (49) |
| | University, n (%) | 82 (38) | 48 (44) | 34 (33) |
| | Missing, n (%) | 1 (0) | 0 (0) | 1 (1) |
| Stage | 0, n (%) | | 11 (10.1) | |
| | I, n (%) | | 52 (47.7) | |
| | II, n (%) | | 25 (22.9) | |
| | III, n (%) | | 20 (18.8) | |
| Previous therapy | Surgery, n (%) | | 42 (38.5) | |
| | Chemotherapy, n (%) | | 19 (17.4) | |
| | Hormone therapy, n (%) | | 2 (1.8) | |
| | No therapy, n (%) | | 46 (42.2) | |
| Ongoing therapy | Chemotherapy, n (%) | | 16 (14.7) | |
| | Hormone therapy, n (%) | | 13 (11.9) | |
| | Radiotherapy, n (%) | | 10 (9.2) | |
| | No therapy, n (%) | | 70 (64.2) | |

*Single: never married or widow or separated

For peer review only

### *2.4.2 Confirmatory factorial analysis*

The Bentler Comparative Fit Index was 0.90, the RMR Estimate 0.07, indicating an adequate model of fit between the model and the data ($\chi^2$=3139.36, DF=276, $p$<.001). Weak factorial invariance suggested acceptable measurement invariance (patients SRMR =0.079, caregivers SRMR= 0.080). By comparing goodness of fit of alternative, more conservative models, difference in RMSEA scores was 0.012, supporting strong factorial invariance [28].



For peer review only - http://bmjopen.bmj.com/site/about/guidelines.xhtml

*2.4.3 Construct validity*

Total scores of FaRE and RSA were positively correlated (ρs= 0.43, *p*<0.0001). Factor Communication and cohesion correlated positively with factor Family Cohesion in RSA (ρs= 0.56, *p*<0.0001). Factor Perceived social support correlated positively with factor Social Resources (ρs= 0.54, *p*<0.0001). Factor Perceived family coping correlated positively with factor Structured Style (ρs= 0.30, *p*<0.0001) and with factor Perception of Self in RSA (ρs= 0.42, *p*<0.0001).

*2.4.5 Concordance between patients' and caregivers' scores*

In this paired analysis, only scores of patients for which a corresponding caregiver's score was available were included. Differential scores were computed for each factor by subtracting the score of the patient from that of the corresponding caregiver. Paired t-tests indicated that the difference for Factor Communication and Cohesion was significantly different from zero (mean difference= -0.27, standard deviation= 0.93, *p*=0.003). No other significant differences were detected (see Supplementary Material).

*2.4.6 Differences between Study 1 and Study 2 samples*

Wilcoxon signed rank tests indicated no differences between the median values of factors in sample in Study 1 and the sample in Study 2, both when considering together the patients and the caregivers, as well as when the patients and the caregivers were compared separately.

*2.4.7 Normative family resilience score*

Due to disparity in number between prostate and breast cancer patients, normative data was calculated on the total breast cancer patients recruited for the two studies (N=317). Mean (5.7) and standard deviation (0.78) for for the total FaRE score was calculated. As with other clinical measures, scores between -/+ 1 SD can be considered in the normal range, scores between -1 and -2 SD could indicate lower levels of family resilience and scores under 2 SD could be considered clinically relevant. For the last two ranges of scores referral to support services would be recommended.



For peer review only - http://bmjopen.bmj.com/site/about/guidelines.xhtml

## 3. Discussion

The FaRE questionnaire is designed to measure family resilience in oncological settings. Adopting a systemic approach, it considers both the patient and the caregiver's strengths and resources in managing the disease. Our initial version of the questionnaire had the same six family resilience constructs addressed by Sixbey[15]; however, a six-factor solution was not supported and it was reduced to four factors. Compared to Sixbey's[15] questionnaire, the FaRE does not include "Making meaning of adversity" and "Positive outlook"; however, the latter is addressed in the Perceived family coping subscale of the FaRE. Sixbey's Connectedness construct is incorporated in Family Communication and Cohesion. While Lane's [29] family resilience measure had highlighted that socio-economic resources had little value to breast cancer survivors; our measure suggests that social resources provide an invaluable support to the family coping with cancer and that economic resources were unrelated to the social ones. The development of the current questionnaire confirms that different resources are activated when families face cancer compared to other adversities and/or the general population.

An important aspect to keep in mind is the low inter-rater reliability between patients and caregivers and this is partly explained by the significant differences found between some FaRE factors. Patients perceived significantly higher levels of family coping and higher levels of social support compared to their caregivers; although the difference in scores was small, it could be explained by the re-direction and allocation of shared coping strategies towards the patient rather than towards the caregiver. The analysis on the patient-caregiver dyads of the second study also showed that caregivers reported significantly lower levels of open communication and emotional sharing compared to the corresponding patient. These results suggest that it is vital to consider the pluralistic view of family members as they are actively involved in cancer management. Further research should try and



For peer review only - http://bmjopen.bmj.com/site/about/guidelines.xhtml

disentangle whether lower support and family coping scores of caregivers are of clinical significance, for instance by assessing the association with mood profiles.

Recruitment of participants from eight different regions of Italy and the combination of cognitive interviews and expert review, which allowed us to determine the relevance of the items, are some of the strengths of the study. Another strength of the developed measure is its systemic view, which allows use in caregiver populations, for whom there are only a few questionnaires available, possibly because attention towards their challenges and needs has increased only in the last few years [30,31].

This said there were some limitations in the development of the current questionnaire. The sample size of the first study is relatively small considering the number of items of the first version of the FaRE; a larger sample could have highlighted missing aspects. Convergent and discriminant validity examining similarities and differences between the FaRE and other family resilience tools would strengthen the rigor of the questionnaire. In addition to this, the cut-off points of the total FaRE scores need validating in future studies in order to use them as screening for referral. In addition to this, it is important to evaluate whether the religiousness-spirituality subscale should be included in the total score of family resilience and whether it plays a key role in promoting resilience levels. To ensure generalisability of the results, a larger sample with diverse tumour diagnosis should be recruited, as our sample was composed of only gender-related tumours with a relatively high life expectancy; in addition to this patients with other chronic and acute illnesses should be sampled to evaluate whether important family resilience aspects in disease management have been dropped. This instrument can be a useful tool to assess patients at baseline and for continued follow-up, for this reason test-retest reliability and responsiveness to change should be conducted.

Preliminary evidence shows that the FaRE is a reliable and valid systemic measure of family resilience in oncological settings. The tool was designed to be implemented in a web platform as a part of the works of an European project, which aimed at promoting cancer patient's empowerment and self-management [32–34]. As increasing attention is being paid to family resilience and its protective



For peer review only - http://bmjopen.bmj.com/site/about/guidelines.xhtml

role in illness trajectories, once further validations are conducted, it can serve as a useful screening tool to identify patients and carers with few resources to deal with cancer related issues. Family resilience screening would allow targeted interventions on development and promotion of specific strategies and resources that can help the family deal with the illness.

**Funding**

This project has received funding from the European Union's Horizon 2020 research and innovation programme under grant agreement No 643529.

**Disclaimer**

This presentation reflects the authors' view. The Commission is not responsible for any use that may be made of the information it contains.

**Declaration of interest**

We have read and understood the BMJ Group policy on declaration of interests and we declare no conflict of interest.

**Author Contributions**

FF has made substantial contributions to article conception and design, was involved in recruitment of patients and in data entry, has given final approval of the version to be published. CR has undertaken the literature review to construct the measure, has developed the items of the questionnaire, has made substantial contributions to article design, has been involved in drafting the manuscript conception the article, has given final approval of the version to be published. CF has undertaken the literature review to construct the measure, has developed the items of the questionnaire, has given final approval of the version to be published. CC was involved in recruitment of patients, data entry, has given final approval of the version to be published. SG has contributed to the analysis and interpretation of data for the work, to drafting the work and provided a critical



For peer review only - http://bmjopen.bmj.com/site/about/guidelines.xhtml

revision for important intellectual content. GP has revised the article critically for important intellectual content, has given final approval of the version to be published.

**Data sharing statement**

Data leading to the results presented in this manuscript are available upon reasonable request from Sara Gandini: sara.gandini@ieo.it

**Acknowledgements**

We thank patients for providing feedback and suggestions for improving the family resilience tool.

**Figure legends**

Figure 1. Eingenvalues and proportion of variance of the factors

For peer review only

## References

1. Rutter M. Implications of resilience concepts for scientific understanding. *Ann N Y Acad Sci*. 2006;1094:1-12. doi:10.1196/annals.1376.002

2. Masten AS. Ordinary magic: Resilience processes in development. *Am Psychol*. 2001;56(3):227-238. doi:10.1037//0003-066X.56.3.227

3. Windle G. What is resilience? A review and concept analysis. *Rev Clin Gerontol*. 2011;21(2):152-169. doi:10.1017/S0959259810000420

4. Strauss B, Brix C, Fischer S, et al. The influence of resilience on fatigue in cancer patients undergoing radiation therapy (RT). *J Cancer Res Clin Oncol*. 2007;133(8):511-518. doi:10.1007/s00432-007-0195-z

5. Wenzel LB, Donnelly JP, Fowler JM, et al. Resilience, reflection, and residual stress in ovarian cancer survivorship: A gynecologic oncology group study. *Psychooncology*. 2002;11(2):142-153. doi:10.1002/pon.567

6. Min JA, Yoon S, Lee CU, et al. Psychological resilience contributes to low emotional distress in cancer patients. *Support Care Cancer*. 2013;21(9):2469-2476. doi:10.1007/s00520-013-1807-6

7. Von Bertalanffy L. General system theory. In: *The Science of Synthesis: Exploring the Social Implications of General Systems Theory*. Boulder, Colorado: University Press of Colorado; 2003:304.

8. Van Schoors M, Caes L, Verhofstadt LL, Goubert L, Alderfer MA. Systematic review: Family resilience after pediatric cancer diagnosis. *J Pediatr Psychol*. 2015;40(9):856-868. doi:10.1093/jpepsy/jsv055

9. Walsh F. Family Resilience: A Framework for Clinical Practice. *Fam Process*. 2003;42(I):1-



For peer review only - http://bmjopen.bmj.com/site/about/guidelines.xhtml

18. doi:10.1111/famp.2003.42.issue-1

10. Faccio F, Renzi C, Giudice A V., Pravettoni G. Family Resilience in the Oncology Setting: Development of an Integrative Framework. *Front Psychol*. 2018;9(666). doi:10.3389/fpsyg.2018.00666

11. McCubbin M, McCubbin HI. Families coping with illness: The resiliency model of family stress, adjustment, and adaptation. *Fam Heal Illn Perspect coping Interv*. 1993:21-63.

12. McCubbin MA, McCubbin HI, Thompson AI. Family hardiness index (FHI). In: *Family Assessment: Resiliency, Coping and adaptation–Inventories for Research and Practice*. ; 1996:239-305.

13. Walsh F. Applying a Family Resilience Framework in Training, Practice, and Research: Mastering the Art of the Possible. *Fam Process*. 2016;55(4):616-632. doi:10.1111/famp.12260

14. Rolland JS, Walsh F. Facilitating family resilience with childhood illness and disability. *Curr Opin Pediatr*. 2006;18(5):527-538. doi:10.1097/01.mop.0000245354.83454.68

15. Sixbey MT. Development of the family resilience assessment scale to identify family resilience constructs. *Dr Diss Univ Florida*. 2005.

16. Isaacs SA, Roman NV, Savahl S, Sui X-C. Adapting and Validating the Family Resilience Assessment Scale in an Afrikaans Rural Community in South Africa. *Community Ment Health J*. 2017;0(0):0. doi:10.1007/s10597-017-0091-1

17. Duca DS. Family resilience and parental stress: the effects on marital relationship in the context of a child diagnosed with an autism spectrum disorder. *Ann AII Cuza Univ Psychol Ser*. 2015;24(1):71-91. http://repository.upenn.edu/edissertations_sp2%0Ahttp://repository.upenn.edu/edissertations



For peer review only

For peer review only - http://bmjopen.bmj.com/site/about/guidelines.xhtml

_sp2/14.

18. Chew J, Haase AM. Psychometric properties of the Family Resilience Assessment Scale: A Singaporean perspective. *Epilepsy Behav*. 2016;61:112-119. doi:10.1016/j.yebeh.2016.05.015

19. Walsh F. *Strengthening Family Resilience*.; 2013. doi:10.1086/514469

20. Lane CD, Meszaros PS. Measuring Walsh ' s Family Resilience Framework : Reliability and Validity of the Family Resilience Assessment ( FRA ) Among Women With a History of Breast Cancer. *Marriage Fam Rev*. 2016:1-16.

21. Messick S. Validity of psychological assessment: validation of inferences from persons' responses and performances as scientific inquiry into score meaning. *ETS Res Rep Ser*. 1994;1994(2):i-28. doi:10.1002/j.2333-8504.1994.tb01618.x

22. Capanna C, Stratta P, Hjemdal O, Collazzoni A, Rossi A. The Italian validation study of the Resilience Scale for Adults (RSA). *Appl Psychol Bull*. 2015;272(63):16-24. http://search.ebscohost.com/login.aspx?direct=true&db=psyh&AN=2015-19177-002&site=ehost-live&scope=site.

23. Fabrigar LR, Wegener DT, Maccallum RC, Strahan EJ. Evaluating the Use of Exploratory Factor Analysis in Psychological Research. *Psychol Methods*. 1999;4(3):272-299.

24. Hatcher RL, Barends A, Hansell J, Gutfreund MJ. Patients' and Therapists' Shared and Unique Views of the Therapeutic Alliance: An Investigation Using Confirmatory Factor Analysis in a Nested Design. *J Consult Clin Psychol*. 1995;63(4):636-643.

25. Cerny BA, Kaiser HF. A Study Of A Measure Of Sampling Adequacy For Factor-Analytic Correlation Matrices. *Multivariate Behav Res*. 1977;12(1):43-47. doi:10.1207/s15327906mbr1201_3



For peer review only - http://bmjopen.bmj.com/site/about/guidelines.xhtml

26. DeVon HA, Block ME, Moyle-Wright P, et al. A Psychometric Toolbox for Testing Validity and Reliability. *J Nurs Scholarsh*. 2007;39(2):155-164. doi:10.1111/j.1547-5069.2007.00161.x

27. Koo TK, Li MY. A Guideline of Selecting and Reporting Intraclass Correlation Coefficients for Reliability Research. *J Chiropr Med*. 2016;15(2):155-163. doi:10.1016/j.jcm.2016.02.012

28. Chen FF. Sensitivity of Goodness of Fit Indexes to Lack of Measurement Invariance. *Struct Equ Model A Multidiscip J*. 2007;14(3):464-504. doi:10.1080/10705510701301834

29. Meszaros PS, Allen KR, Few-demo AL, Piercy FP, Savla JS. Tracing the Pink Ribbon : Development of a Family Resilience Measure Crystal Lynn Duncan Lane Dissertation submitted to the faculty of the Virginia Polytechnic Institute and State University in partial fulfillment of the requirements of the degree of Doct. 2011.

30. Kim Y, Baker F, Spillers RL, Wellisch DK. Psychological adjustment of cancer caregivers with multiple roles. *Psychooncology*. 2006;15(9):795-804. doi:10.1002/pon.1013

31. Sklenarova H, Krümpelmann A, Haun MW, et al. When do we need to care about the caregiver? Supportive care needs, anxiety, and depression among informal caregivers of patients with cancer and cancer survivors. *Cancer*. 2015;121(9):1513-1519. doi:10.1002/cncr.29223

32. Kazantzaki E, Koumakis L, Kondylakis H, et al. Current trends in Electronic Family Resilience Tools: Implementing a tool for the cancer domain. In: ; 2018:29-32. doi:10.1007/978-981-10-5122-7_8

33. Renzi C, Fioretti C, Mazzocco K, et al. Development of psycho-emotional monitoring tools within an eHealth platform to improve patient empowerment and self-management abilities. *Psychooncology*. 2016;25(3):195.



For peer review only - http://bmjopen.bmj.com/site/about/guidelines.xhtml

34. Faccio F, Renzi C, Crico C, et al. Development of an eHealth tool for cancer patients: monitoring psychoemotional aspects with the family resilience (FaRe) questionnaire. *Ecancermedicalscience*. 2018;12. doi:10.3332/ecancer.2018.852

For peer review only



For peer review only - http://bmjopen.bmj.com/site/about/guidelines.xhtml

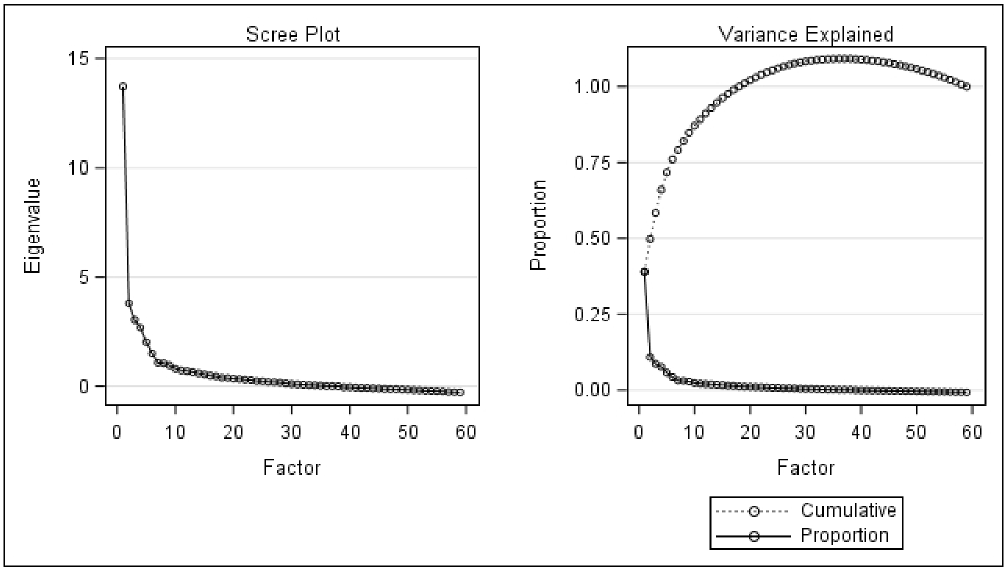


Figure 1. Eigenvalues and proportion of variance of the factors

# Supplementary Material

Appendix A. *Family Resilience Questionnaire*

Dear Sir/Madam,

Please read each of the following statements carefully. For each one, state how well it describes your family. In thinking about your family, you can include all the significant people you think are part of it.

1. We understand each other with regard to the experience of illness we are living

Strongly disagree Moderately disagree Slightly disagree Nor Disagree nor agree Slightly Agree Moderately Agree Strongly agree

2. We ask our closest friends to help and assist us in this battle against the illness

Strongly disagree Moderately disagree Slightly disagree Nor Disagree nor agree Slightly Agree Moderately Agree Strongly agree

3. We believe that we can manage the illness

Strongly disagree Moderately disagree Slightly disagree Nor Disagree nor agree Slightly Agree Moderately Agree Strongly agree

4. In our family we feel that we can talk about how to communicate between us

Strongly disagree Moderately disagree Slightly disagree Nor Disagree nor agree Slightly Agree Moderately Agree Strongly agree

5. We can rely on our close friends to help us deal this illness

Strongly disagree Moderately disagree Slightly disagree Nor Disagree nor agree Slightly Agree Moderately Agree Strongly agree

For peer review only

6. We attend the church/synagogue/mosque/other places of worship

Strongly disagree Moderately disagree Slightly disagree Nor Disagree nor agree Slightly Agree Moderately Agree Strongly agree

7. We can deal this illness as a family

Strongly disagree Moderately disagree Slightly disagree Nor Disagree nor agree Slightly Agree Moderately Agree Strongly agree

8.We feel that the people in our social network would be happy to support us emotionally in dealing the illness

Strongly disagree Moderately disagree Slightly disagree Nor Disagree nor agree Slightly Agree Moderately Agree Strongly agree

9. We can solve important problems in our life such as this illness

Strongly disagree Moderately disagree Slightly disagree Nor Disagree nor agree Slightly Agree Moderately Agree Strongly agree

10. We discuss the illness-related problems until we find a shared solution

Strongly disagree Moderately disagree Slightly disagree Nor Disagree nor agree Slightly Agree Moderately Agree Strongly agree

11. We feels that our closest friends would be happy to support us emotionally in managing the illness

Strongly disagree Moderately disagree Slightly disagree Nor Disagree nor agree Slightly Agree Moderately Agree Strongly agree

12. We believe there is a supreme spiritual being that will help us deal this illness



For peer review only

For peer review only - http://bmjopen.bmj.com/site/about/guidelines.xhtml

Strongly disagree Moderately disagree Slightly disagree Nor Disagree nor agree Slightly Agree Moderately Agree Strongly agree

13. Everyone in the family feels free to express their own opinion regarding the illness

Strongly disagree Moderately disagree Slightly disagree Nor Disagree nor agree Slightly Agree Moderately Agree Strongly agree

14. We know that if we need comfort, our closest friends will be there for us

Strongly disagree Moderately disagree Slightly disagree Nor Disagree nor agree Slightly Agree Moderately Agree Strongly agree

15. We feel we are strong enough to cope with this illness

Strongly disagree Moderately disagree Slightly disagree Nor Disagree nor agree Slightly Agree Moderately Agree Strongly agree

16. We are honest when talking about the illness amongst ourselves

Strongly disagree Moderately disagree Slightly disagree Nor Disagree nor agree Slightly Agree Moderately Agree Strongly agree

17. We know we are important for our friends

Strongly disagree Moderately disagree Slightly disagree Nor Disagree nor agree Slightly Agree Moderately Agree Strongly agree

18. We participate in the activities of our religious community

Strongly disagree Moderately disagree Slightly disagree Nor Disagree nor agree Slightly Agree Moderately Agree Strongly agree



For peer review only - http://bmjopen.bmj.com/site/about/guidelines.xhtml

19. Everyone in the family is open to listening other's opinions regarding the illness

Strongly disagree Moderately disagree Slightly disagree Nor Disagree nor agree Slightly Agree Moderately Agree Strongly agree

20. We receive gifts and favours from our closest friends

Strongly disagree Moderately disagree Slightly disagree Nor Disagree nor agree Slightly Agree Moderately Agree Strongly agree

21. We have the strength to solve our problems

Strongly disagree Moderately disagree Slightly disagree Nor Disagree nor agree Slightly Agree Moderately Agree Strongly agree

22. The things we do for each other in dealing with this illness make us feel part of the family

Strongly disagree Moderately disagree Slightly disagree Nor Disagree nor agree Slightly Agree Moderately Agree Strongly agree

23. Our friends respect our family for how we reacted to the illness

Strongly disagree Moderately disagree Slightly disagree Nor Disagree nor agree Slightly Agree Moderately Agree Strongly agree

24. We ask our religious/spiritual reference figure for advice or words of comfort about the illness

Strongly disagree Moderately disagree Slightly disagree Nor Disagree nor agree Slightly Agree Moderately Agree Strongly agree



For peer review only

For peer review only - http://bmjopen.bmj.com/site/about/guidelines.xhtml

Appendix B. *Tables of biological descriptive statistics of Study 1 and differential scores in patient-caregiver dyads*

Table 1. Tumor characteristics of the first sample

| | | Total (n=104) | Breast (n=53) | prostate (n=51) |
|---|---|---|---|---|
| Lymph nodes | No | 80 (76.9) | 40 (75.5) | 40 (78.4) |
| | Yes | 22 (21.2) | 13 (24.5) | 9 (17.6) |
| | Missing | 2 (1.9) | 0 (0) | 2 (3.9) |
| Metastasis | No | 101 (97.1) | 53 (100) | 48 (94.1) |
| | Yes | 1 (1) | 0 (0) | 1 (2) |
| | Missing | 2 (1.9) | 0 (0) | 2 (3.9) |
| Stage | I | 15 (14.4) | 11 (20.8) | 4 (7.8) |
| | II | 52 (50) | 33 (62.3) | 19 (37.3) |
| | III-IV | 35 (33.7) | 9 (17) | 26 (51) |
| | Missing | 2 (1.9) | 0 (0) | 2 (3.9) |
| Grading | 1-2 | 39 (37.5) | 26 (49.1) | 13 (25.5) |
| | 3-4 | 64 (61.5) | 27 (50.9) | 37 (72.5) |
| | Missing | 1 (1) | 0 (0) | 1 (2) |
| Previous therapy | No | 28 (26.9) | 12 (22.6) | 16 (31.4) |
| | Yes | 75 (72.1) | 41 (77.4) | 34 (66.7) |
| | Missing | 1 (1) | 0 (0) | 1 (2) |

Table 2. Differences in family resilience scores in patient-caregiver dyads in the first study

| **Differences pt-CG** | **Median** | **Low** | **Up** |
|---|---|---|---|
| factor1_com | 0 | -0.38 | 0.5 |
| factor2_supp | 0 | -0.5 | 0.75 |
| factor3_coping | 0.13 | -0.38 | 1 |
| factor4_spirit | -0.25 | -1 | 1 |

Low: lower quartile; Up: upper quartile

Factor1_com = Communication and cohesion; Factor2_supp= Perceived social support; Factor3_coping = Perceived family coping; Factor4_spirit = Religiousness and Spirituality

Table 3. Mean differential scores and paired-t test between patient-caregiver dyads in the second study

| | Mean | SD | p-value |
|---|---|---|---|
| **diff_factor1** | **-0.27** | 0.93 | **0.003** |



For peer review only - http://bmjopen.bmj.com/site/about/guidelines.xhtml
For peer review only

| | | | |
|---|---|---|---|
| diff_factor2 | 0.21 | 1.17 | 0.074 |
| diff_factor3 | -0.14 | 1.05 | 0.179 |
| diff_factor4 | 0.27 | 1.72 | 0.111 |

Factor1 = Communication and cohesion; Factor2= Perceived social support; Factor3= Perceived family coping; Factor4 = Religiousness and Spirituality

Table 4. Eingenvalues and proportion of variance of the factors

| | Eigenvalue | Difference | Proportion | Cumulative |
|---|---|---|---|---|
| **1** | **13.72** | **9.92** | **0.39** | **0.39** |
| **2** | **3.81** | **0.77** | **0.11** | **0.50** |
| **3** | **3.04** | **0.35** | **0.09** | **0.58** |
| **4** | **2.69** | **0.66** | **0.08** | **0.66** |
| 5 | 2.02 | 0.52 | 0.06 | 0.72 |
| 6 | 1.51 | 0.42 | 0.04 | 0.76 |
| 7 | 1.08 | 0.03 | 0.03 | 0.79 |
| 8 | 1.06 | 0.09 | 0.03 | 0.82 |
| 9 | 0.96 | 0.15 | 0.03 | 0.85 |

For peer review only



For peer review only - http://bmjopen.bmj.com/site/about/guidelines.xhtml

BMJ Open

# Development and validation of a Family Resilience questionnaire (FaRE): an observational study in Italy



| | |
|---|---|
| Journal: | *BMJ Open* |
| Manuscript ID | bmjopen-2018-024670.R3 |
| Article Type: | Research |
| Date Submitted by the Author: | 26-Mar-2019 |



| | |
|---|---|
| Complete List of Authors: | Faccio, Flavia; Università degli Studi di Milano, Department of Oncology and Hemato-Oncology; Istituto Europeo di Oncologia, Applied Division for Cognitive and Psychological Science<br>Gandini, Sara; European Institute of Oncology, Division of Epidemiology and Biostatistics<br>Renzi, Chiara; European Institute of Oncology, European Institute of Oncology<br>Fioretti, Chiara; Università degli Studi di Firenze, Department of Educational Sciences and Psychology<br>Crico, Chiara; Università degli Studi di Milano, Department of Oncology and Hemato-Oncology; Istituto Europeo di Oncologia, Applied Division for Cognitive and Psychological Science<br>Pravettoni, Gabriella; Istituto Europeo di Oncologia, Applied Research Division for Cognitive and Psychological Science; Universita degli Studi di Milano, Department of Oncology and Hemato-oncology |



| | |
|---|---|
| <b>Primary Subject Heading</b>: | Patient-centred medicine |
| Secondary Subject Heading: | General practice / Family practice, Oncology, Research methods, Patient-centred medicine |
| Keywords: | Family Resilience, Stress and coping, Surveys and Questionnaires, Systems theory, ONCOLOGY, Family Adaptation |
| | |


SCHOLARONE™
Manuscripts

# Development and validation of a Family Resilience questionnaire (FaRE): an observational study in Italy

*Flavia Faccio[1,2‡], Sara Gandini[3], Chiara Renzi[1], Chiara Fioretti[4], Chiara Crico[1,2], Gabriella Pravettoni[1,2]

[1] Applied Research Division for Cognitive and Psychological Science, IEO, European Institute of Oncology IRCCS, Milan, Italy

[2] Department of Oncology and Hemato-Oncology, University of Milan, Milan, Italy

[3]Division of Epidemiology and Biostatistics, IEO, European Institute of Oncology IRCCS, Milan, Italy

[4]Department of Educational Sciences and Psychology, Università degli Studi di Firenze, Florence, Italy

Email addresses flavia.faccio.90@gmail.com; sara.gandini@ieo.it; chiara.renzi@ieo.it; chiara.fioretti@unifi.it; chiara.crico@ieo.it; vgabriella.pravettoni@ieo.it

Word count: 4601

‡Corresponding author:

Flavia Faccio, flavia.faccio.90@gmail.com, Telephone: +39 029437099, Applied Research Unit for Cognitive and Psychological Science, European Institute of Oncology, Milan, Via Ripamonti 435.



For peer review only - http://bmjopen.bmj.com/site/about/guidelines.xhtml

**Abstract**

*Objective:* Develop and validate an instrument to assess family resilience and, more specifically, the family dynamics and resources, estimating the adaptation flexibility to cancer disease. Cohesion, communication, coping style, and relational style were considered as critical functional areas in the construction of the instrument.

*Design*: Two cross-sectional studies. Study 1: Identification of factorial structure of the questionnaire in two samples with different cancer sites. Study 2: Validation of the questionnaire in cancer patients in two different phases of their therapeutic pathway.

*Participants and setting:* A total of 213 patients with a histologically confirmed non-metastatic breast or prostate cancer and 209 caregivers were recruited for the two studies from an oncological hospital in Italy.

*Outcome measures*: The Resilience Scale for Adults and the Family Resilience questionnaire (FaRE) developed by the researchers were administered to all patients and caregivers that gave consent.

*Results:* In Study 1, the 60-item version of the FaRE underwent discriminant and construct validity, internal consistency, and factorial analysis. Comparisons between patient and caregiver populations showed that patients perceived higher levels of family resources ($p$=0.048) and that prostate cancer patients perceive less social support compared to breast cancer ($p$=0.002). Factor analysis demonstrated four domains: Communication and cohesion, Perceived social support, Perceived family coping, and Religiousness and spirituality. In Study 2 the validity and factorial structure of the final scale, composed of 24 items, were confirmed. The Cronbach's alpha of all subscales was above .82. Normative values for breast cancer patients can provide indications of family resilience levels.

*Conclusions:* Preliminary findings showed acceptable psychometric properties for the FaRE to evaluate family resilience in oncological patients and their caregivers. Further research should test




For peer review only - http://bmjopen.bmj.com/site/about/guidelines.xhtml

its sensibility to change to assess its use as a psycho-emotional monitoring too and its validity in other medical contexts.

**Keywords**

Family Resilience, Family Adaptation, Stress and coping, Surveys and Questionnaires, Systems Theory, Oncology

**Article summary**

***Strengths and limitations of this study***

- Questionnaire development and testing was conducted in two phases in order to assess validity and reliability of the final version of the measure.
- This is the first measure of family resilience that adopts a systemic approach, considering both the patient and caregiver, in light of increasing evidence on the vital role the caregiver.
- Results were controlled for the possible confounding sociodemographic variables.
- Only two oncological populations were recruited, making generalisability to all types of tumour patients limited.
- Longitudinal measurements are advised to investigate test-retest reliability and responsiveness to change of the measure.

For peer review only

## Introduction

Resilience is a multifaceted concept that can be described as the ability to mobilize resources and adapt to challenging or adverse situations [1,2]. One of the internal resources of resilience is coping, which consists of a set of modifiable skills that can be learnt to deal with a stressful life event[3]. More and more often, resilience is studied as a relevant aspect when facing a disease such as cancer [4–6]. However, the impact of disrupting events such as a cancer diagnosis, is not limited only to the individual, rather it influences also their family and social network, which can initiate or support positive adaptation as well as be overwhelmed by the demands of the disease and its treatments. According to the family systems theory [7], a change or a perturbation occurring to one member of the system will affect also the other members, which may result in a smooth adaptation to a new homeostasis or in difficulties that prevent the readjustment process [7]. This translates into clinically significant levels of distress, higher risk of developing psychosocial problems, high levels of conflict and low family cohesion[8]. Going beyond the idea that families which encounter difficulties are "damaged" or "pathological" [9], resilience may represent a pivotal concept to analyse and support the family in moving towards a new balance and in constructing meaning of the cancer event [10].

A substantial contribution to research in family resilience comes from two main models: the Resiliency Model of Family Adjustment and Adaptation[11] and the Family Resilience Framework[9], both of which have been applied to oncological settings. Walsh's framework was chosen for various reasons: first, conceptualisation of family resilience in McCubbin's model is quite rigid, it belongs to a first wave of resilience research that believed one has to progress through specific steps in order to achieve resilience. In addition to this, due to the large number of variables involved in the model, it was possible for previous studies to validate only parts of it, never in its entirety[12]. Differently, Walsh [9,13] defines resilience as the family's ability to "withstand and rebound from adversity, strengthened and more resourceful", including the capacity to cope and adapt to the stressor and the concept of post-traumatic growth. She considers three major dimensions as contributing to the system's



For peer review only - http://bmjopen.bmj.com/site/about/guidelines.xhtml

resilience: i) belief systems, ii) organizational patterns and iii) communication processes. Each of these overarching constructs is composed of three subprocesses: Make meaning of adversity, Positive outlook, Transcendence and spirituality, Flexibility, Connectedness, Social and economic resources, Communication/problem-solving, Clarity, Open emotional expression, Collaborative problem-solving. These are considered mutually interactive and synergistic as they facilitate and sustain each other across systems and over time [13]. Walsh also proposed an integrative family resilience model for illness related challenges, including cancer[14]; currently, there is no quantitative evidence regarding the framework's efficacy.

Only three questionnaires have attempted to capture the multiple dimensions of Walsh's[9] framework. Sixbey [15] developed the Family Resilience Assessment Scale (FRAS), a 66-item measure with responses given on a 4-point Likert scale. The Statistical considerations and factor loadings showed that a nine construct model of family resilience was not supported and therefore the number of factors was reduced to six [15]: i) family communication and problem-solving, ii) social and economic resources, iii) maintaining a positive outlook, iv) family connectedness, v) family spirituality and vi) making meaning of adversity. One of the subscales, Communication and Problem-solving, is an overarching construct of Walsh's model, which originally contained three sub-processes The questionnaire was administered to the general American population without considering an essential aspect of resilience, namely whether an adversity had occurred. A second validation study suggested that the FRAS could be a reliable and valid measure in a population which was not experiencing an adversity [16]. This is the major weakness of the measure, which has then been administered to families that have experienced difficult diagnoses, such as autism spectrum disorder [17] and epilepsy [18], where disagreement regarding the number of factors of the scale is highlighted[18]. In addition to this, the measure was never administered to multiple members of a family, therefore the perspective of the questionnaire is individualistic and is not representative of the family unit as a whole. As individual



For peer review only - http://bmjopen.bmj.com/site/about/guidelines.xhtml

perceptions of which resilience resources are activated may vary, it is essential to compare the views of different family members[19].

The other two measures developed according to Walsh's framework are the Family Resilience Assessment (FRA; Lane & Meszaros, 2016) and the Walsh Family Resilience Questionnaire (WFRQ; Walsh, 2013). The former is composed of 29 items on a 5-point scale response and was validated in a sample of women with a history of breast cancer. The developers [20] warned that the items did not always reflect Walsh's indicators, decided to group the items in the three overarching dimensions and suggested that socio-economic resources have little value in breast cancer survivors. Lane implies that further studies should capture the pluralistic view of the family members[20]. To date the FRA has not been used in other oncological or more general health contexts, suggesting the need for further studies in order to consider the instrument a valid measure. In addition to this, the recruited sample was small and different subgroups were present: survivors who had received diagnosis in the past 15 years, others in the past 5 years, another group of women undergoing treatment, of which half has a stage IV breast cancer diagnosis. Therefore, both current and retrospective accounts of resilience were collected. Finally, Walsh [19] developed her own self-report questionnaire, composed of 33 items that operationalise her nine key processes and provide a map of each family. While she states that her questionnaire is being applied in several international projects, data from these studies has not been published yet.

As there is only one measure of family resilience in cancer patients [15] which has an individualistic view of resilience and does not capture the systemic processes involved, a new measure including Sixbey's six factors was developed. These factors were chosen compared to Lane's three factors as, in our opinion, not all salient aspects of family resilience in cancer had been considered and to avoid construct underrepresentation, one of the major threats to construct validity[21]. The aim of this measure is to investigate family resources and strengths during cancer management, taking into account both the patient's and the caregiver's perspective.



For peer review only - http://bmjopen.bmj.com/site/about/guidelines.xhtml

## Material and methods

All study phases were presented in a single protocol to the local Ethics Committee, which was approved in all its parts on the 11th of April, 2016. The aim of the first study was to develop a questionnaire, the Family Resilience Evaluation Questionnaire (FaRE) to assess resilience in families affected by cancer. Following Messick's[21] guidelines for validating a measure, the following stages were conducted: substantive, structural, and external. While substantive and structural steps were undertaken in Study 1, the external stage is present in Study 2.

### 1. Study 1: Item generation phase and development of the questionnaire

For the substantive stage, a first version of the FaRE was constructed by the authors after a thorough revision of scientific literature in the field of resilience and cancer and after conducting informal, clinical appointments with 20 cancer patients about strategies that helped them adjust and cope with the illness. The questionnaire was then developed and assessed by an expert panel composed of psychologists that conduct research in the field of resilience. Some minor adjustments to the questions was made and five items were dropped as they were considered too similar to other statements. Finally, this preliminary version of the FaRE was administered to a pilot group of 10 citizens, 10 cancer patients and 10 caregivers comparable to the target population in order to evaluate item comprehensibility, provide suggestions and indicate which items seemed unclear. Six items were re-worded to improve understanding. The second version of the questionnaire was composed of 60 items referring to six aspects: 1) family communication and problem solving, 2) social and economic resources, 3) maintaining a positive outlook, 4) family connectedness, 5) family spirituality, and 6) ability to make meaning from adversity. Questions were on a seven-point Likert scale (from Strongly Agree to Strongly Disagree).



For peer review only - http://bmjopen.bmj.com/site/about/guidelines.xhtml

## *1.2 Item selection phase*

### *1.2.1 Participants*

Couples of patients and caregivers that fitted the inclusion criteria were asked to participate in the current study. Participation was proposed to a total of 146 couples, 42 of which refused due to little interest in the study or lack of time. 105 patients (53 breast, 52 prostate cancer patients) and 105 caregivers (i.e., son/daughter, wife/husband/partner, father/mother, sister/brother) were recruited for the study at a comprehensive cancer centre in northern Italy. Patients were recruited from two outpatient clinics, one of the Breast Cancer Division, the second of the Urology Division. In addition to this, patients receiving oncological treatment were recruited from the Radiotherapy Division and Day Hospital. If compatible with the researcher's availability, consecutive patients who were waiting for their appointment were enrolled in the study. Once the study's aims and procedures were illustrated, all participants gave written informed consent.

Inclusion criteria for patients were the following: 1) between 25 and 80 years of age; 2) non-metastatic cancer diagnosis of breast or prostate cancer; 4) no psychiatric, addictive, or cognitive disorders that would prevent compliance with protocol requirements.

The inclusion criteria for caregivers were: 1) over 18 years old; 2) had a relative with a diagnosis of breast or prostate cancer; 3) no psychiatric, addictive, or cognitive disorders that would prevent compliance with protocol requirements.

Due to incomplete answers of one patient his data was not included in the database, therefore the final sample was composed by 104 patients and 105 caregivers.

### *1.2.2 Materials*

The revised version of the FaRE, obtained at the end of the pilot study, was composed of 60 items and it was administered to patients and caregivers participating to the study.



For peer review only - http://bmjopen.bmj.com/site/about/guidelines.xhtml

The Italian version of the Resilience Scale for Adults (RSA) [22] was chosen to assess the convergent validity of the FaRE. The RSA is a 33 item self-report scale for adults that measures six resilience factors: Perception of Self, Planned Future, Social Competence, Structured Style, Family Cohesion, and Social Resources. It provides a total mean score, with higher scores indicating higher resilience. Four factors measure the individual's characteristics, one the family environment and the last factor social networks. Cronbach's alphas for the Italian version ranged from 0.75 to 0.90 for all subscales and total score with the exception of Structured Style (0.34).

*1.2.3 Patient involvement and procedure*

The patients in this study were participants. They were not involved in the development of the research question or in the recruitment phase. However, patients were asked for feedback on the readability of the questionnaire in the pilot phase and their suggestions were incorporated in the final version of the FaRE.

Participants were approached by the researcher while they were in the waiting hall for the appointment with a healthcare professional (oncologist, surgeon, or nurse). The researcher explained the purposes of the study and asked both the patient and the caregiver if they were interested in participating. If they answered affirmatively, an appointment was made after their medical consultation, during which further details were given, together with an informed consent that was signed by both the researcher and the participants. The questionnaires were administered on an iPad; for those who did not use electronic devices paper-and-pencil versions of the questionnaires were given.

*1.2.4 Statistical analyses*

All statistical analyses were conducted using SAS (Statistical Analysis System) version 9.2. The first step was an exploratory factor analysis (EFA) to reduce the item pool [23]. The EFA was conducted using maximum likelihood factoring with orthomax rotation. Several criteria provided guidelines in selecting the number of factors to extract [24]. Factors with eigenvalues greater than 1.0 were retained



For peer review only - http://bmjopen.bmj.com/site/about/guidelines.xhtml

and an examination of scree plots of eigenvalues and total variance explained by retained factors helped to determine the total number of factors to retain (see Figure 1). More generally, in the early stages and throughout the entire process, the items retained were those with the highest explained variance for each factor.

Kaiser Meyer Olkin's test for sampling adequacy was conducted on the sample to evaluate the adequacy of data.[25]

A Cronbach coefficient alpha was calculated for the entire scale and for each individual subscale to assure internal consistency and reliability. To further assess internal reliability, additional indices were computed: Average Variance Extracted (AVE) values and Joreskog rho values[26,27].

To evaluate the goodness of fit between the models and factorial invariance we used fit indices, including the Bentler comparative fit index (CFI), the RMSEA that indicates the amount of unexplained variance and the Standardised Root Mean Square Residuals (SRMR).

Intraclass correlations (ICC) were computed to measure interrater reliability.

Median values, Standard deviation and first and third quartiles are presented for total and subscale FaRE scores. Values are presented also for patients and caregivers separately, by age groups, levels of education, marital status, and number of children and they are compared by Wilcoxon rank tests. We did not expect differences between sociodemographic categories while we expected that differences could arise between patients and caregivers, as activation of resources can differ depending on the role of the family member in adjusting to the illness.

In order to investigate concordance between patients' and caregivers' scores, only scores of patients for which a corresponding caregiver's score was available were included. Differential scores were computed for each factor by subtracting the score of the patient from that of the corresponding caregiver. Paired t-tests were calculated to test the differences.

### *1.2.5 Results*



For peer review only

For peer review only - http://bmjopen.bmj.com/site/about/guidelines.xhtml

*1.2.5.1 Descriptive statistics*

The median age of the population was 60 years old, with an interquartile range (IQR) from 51 to 66.

Sixty percent of caregivers were female. 71% of caregivers were wife/husband/partner, 14% were son/daughter, 11% were sister/brother, and the remaining 4% were mother/father to the patient. The majority of the participants was married or in a relationship (83.7% and 81% for patients and caregivers respectively) and had children (84.6% and 76.2% for patients and caregivers respectively). Most participants had a high school degree (41.3% for patients, 44% for caregivers) or higher (33.7% for participants, 29% for caregivers). No sociodemographic variables were significantly different between groups. Further descriptive data can be found in Table 1.

Table 1. Sociodemographic characteristics of the sample

| | | Total (N=209) | Patients (N=104) | CG (N=105) | P-values |
|---|---|---|---|---|---|
| Age | <50, n (%) | 43 (20.6) | 22 (21.2) | 21 (20) | 0.84 |
| | ≥50, n (%) | 162 (77.5) | 80 (76.9) | 82 (78.1) | |
| | Missing, n (%) | 4 (1.9) | 2 (1.9) | 2 (1.9) | |
| Gender | Male, n (%) | 93 (44.5) | 51 (49) | 42 (40) | 0.19 |
| | Female, n (%) | 116 (55.5) | 53 (51) | 63 (60) | |
| Education | Elementary-middle, n (%) | 49 (23.4) | 23 (22.1) | 26 (24.8) | 0.76 |
| | High school, n (%) | 90 (43.1) | 43 (41.3) | 47 (44.8) | |
| | University, n (%) | 66 (31.6) | 35 (33.7) | 31 (29.5) | |
| | Missing, n (%) | 4 (1.9) | 3 (2.9) | 1 (1) | |
| Marital status | Single*, n (%) | 35 (16.7) | 17 (16.3) | 18 (17.1) | 0.83 |
| | Married, n (%) | 172 (82.3) | 87 (83.7) | 85 (81) | |
| | Missing, n (%) | 2 (1) | 0 (0) | 2 (1.9) | |
| Children | No, n (%) | 39 (18.7) | 16 (15.4) | 23 (21.9) | 0.36 |
| | 1-2, n (%) | 133 (63.6) | 68 (65.4) | 65 (61.9) | |
| | >2, n (%) | 35 (16.7) | 20 (19.2) | 15 (14.3) | |
| | Missing, n (%) | 2 (1) | 0 (0) | 2 (1.9) | |

CG: Caregiver; *Single: never married or widow or separated

Around 83% of breast and 45% of prostate cancer patients were diagnosed with Stage I-II, the remaining patients had stage III-IV tumours. All breast and most prostate patients had undergone at least one type of oncological therapy before (radiotherapy, chemotherapy and/or surgery). One



For peer review only - http://bmjopen.bmj.com/site/about/guidelines.xhtml

prostate patient underwent PET scan for tumour stadiation several weeks after completing the questionnaires and the test indicated the presence of metastases. Further clinical data can be found in the Supplementary Material.

*1.2.5.2 Factorial analysis*

Kaiser Meyer Olkin's test for sampling adequacy were above 0.8 (overall KMO= 0.92, patient sample KMO= 0.9, caregiver sample KMO=0.84), indicating that the data was suitable for factorial analysis[25].

Explained variance was over 10% for 4 factors, for the remaining two factors the explained variance was under 5%, which is considered too low to be retained and were therefore excluded. Factor 5 had only two items, Factor 6 three and in both factors the items were unrelated to each other. EFA indicated 4 factors: one factor (Factor 5) of the initial version of the questionnaire was removed ("Ability to make meaning from adversity") and some items of two factors, namely "Family communication and problem solving" and "Family connectedness" were collapsed into a new subscale, Communication and Cohesion (see Supplementary material). For each factor, the items with the highest weights were selected. The number of items for factors Communication and cohesion and Perceived social support is eight, for Perceived family coping, Religiousness and spirituality four.

A sufficient model fit (Bentler CFI) was defined as of 0.90 or greater, the RMSEA of 0.06 or less[28]. The Bentler CFI was 0.94 ($\chi^2$= 2571.16, DF=276, $p$<.001), the RMSEA Estimate 0.05, indicating an adequate fit between the model and the data.

*1.2.5.3 Final version of questionnaire*

The final factors of the family resilience questionnaire are four (see Supplementary Material). Communication and cohesion (items 1, 4, 7, 10, 13, 16, 19, 22) refers to the family's openness in communicating about the illness and its impact on their daily life. It also involves shared problem-solving and decision-making, resolution of conflict, openness regarding the range of feelings they are



For peer review only - http://bmjopen.bmj.com/site/about/guidelines.xhtml

experiencing. The second factor, Perceived social support (items 2, 5, 8, 11, 14, 17, 20, 23), refers to mutual support, reaching out to extended kin and community for help with practical and emotional tasks. The third factor, Perceived family coping (items 3, 9, 15, 21), includes the ability to activate coping strategies and develop inner strength to deal with illness management. The concept of bouncing back and the ability to rebound from this stressful life event is present in this subscale. The last factor, Religiousness and spirituality (items 6, 12, 18, 24), refers to spiritual values, transcendent beliefs and congregational support.

*1.2.5.4 Internal consistency*

An alpha of 0.70 is nowadays considered as acceptable for a new instrument [29]. The alpha computed for each of the four subscales exceeded the minimum value for a new tool. Cronbach's alphas for each factor were 0.88, 0.88, 0.82, and 0.86, respectively. Average Variance Extracted (AVE) values were 0.52, 0.48, 0.58, 0.71 and Joreskog rho values were 0.90, 0.88, 0.84, 0.91, respectively. As an AVE value of 0.5 or more indicates good reliability and a rho value of 0.70 indicates acceptable reliability it is possible to assume adequate internal consistency[26,27].

*1.2.5.5 Comparison between patients and caregivers*

All FaRE ICC coefficients were between 0.44 and 0.53, indicating an overall weak to moderate interrater reliability[30], suggesting that there is variation between patients and caregivers in rating the family resilience constructs.

Perceived family coping was significantly different between patients and caregivers, the former perceiving higher levels of family resources (median score patients=6.3; median score caregivers=6.0; $p$=0.048). A comparison was carried out also within patient-caregiver dyads and the difference was not significant for any factor (see Supplementary Material).

*1.2.5.6 Comparison between cancer patients' populations*



For peer review only - http://bmjopen.bmj.com/site/about/guidelines.xhtml

Perceived social support was significantly different between breast and prostate cancer patients. The former reported receiving greater support from family and friends for their disease compared to the latter ($p$=0.002). Table 2 details the results of the non-parametric tests.

Table 2. Non-parametric tests on FaRe factors

| | Patients (n=104) | | | | CG (n=105) | | | | |
|---|---|---|---|---|---|---|---|---|---|
| **Factors** | **Median** | **Stdev** | **Q1** | **Q3** | **Median** | **Stdev** | **Q1** | **Q3** | **P-values** |
| Communication and cohesion | 6.6 | 0.9 | 5.7 | 7.5 | 6.5 | 0.8 | 5.7 | 7.3 | 0.238 |
| Perceived social support | 5.9 | 1.2 | 4.7 | 7.0 | 5.8 | 1.1 | 4.6 | 6.9 | 0.542 |
| Perceived family coping | 6.3 | 0.8 | 5.4 | 7.1 | 6.0 | 1.0 | 5.0 | 7.0 | **0.048** |
| Religiousness and Spirituality | 4.5 | 1.8 | 2.7 | 6.3 | 4.8 | 1.9 | 2.9 | 6.6 | 0.341 |

| | Breast (n=53) | | | | Prostate (n=51) | | | | |
|---|---|---|---|---|---|---|---|---|---|
| **Factors** | **Median** | **Stdev** | **Q1** | **Q3** | **Median** | **Stdev** | **Q1** | **Q3** | **P-values** |
| Communication and cohesion | 6.6 | 0.7 | 5.9 | 7.4 | 6.8 | 1.1 | 5.7 | 7.8 | 0.180 |
| Perceived social support | **6.3** | **0.8** | **5.4** | **7.1** | **5.5** | **1.3** | **4.2** | **6.8** | **0.002** |
| Perceived family coping | 6.0 | 0.9 | 5.1 | 6.9 | 6.5 | 0.7 | 5.8 | 7.2 | 0.171 |
| Religiousness and Spirituality | 4.8 | 1.8 | 3.0 | 6.5 | 4.3 | 1.9 | 2.3 | 6.2 | 0.285 |

P-values from Wilcoxon rank tests. Stdev: Standard deviation. Q1 and Q3 are the first and third quartiles.

# Material and Methods

## 2. Study 2: Validation of the FaRE questionnaire

The second study was designed to validate the factorial structure of the questionnaire in a new sample of breast cancer patients in two different phases of their therapeutic pathway: pre-admission phase (either for day-surgery admission or for ordinary surgery admission) and treatment phase (surgery, radiation therapy, or chemotherapy). In addition to this, the external stage of validation was conducted by assessing construct validity with some of the RSA and FaRE subscales. We expected to see positive correlations between: i) RSA and FaRE total scores; ii) RSA Family Cohesion subscale and FaRE Communication and Cohesion subscale; iii) RSA Social Resources and FaRE Perceived social support. While total scores should correlate as some individual resources could reflect in family resilience, the other two correlations were expected as they had common underpinning psychological



For peer review only - http://bmjopen.bmj.com/site/about/guidelines.xhtml

constructs. Finally, significant correlations were hypothesized between RSA Perception of Self and FaRE Family coping, as the RSA factor incorporates positive outlook, confidence and self-efficacy, aspects which are present in the systemic view of the Family coping subscale.

*2.1 Participants*

A total of 143 couples was approached to explain the study, 34 couples refused due to time constraints between appointments or due to little interest in participating. Only breast cancer patients were recruited at this time due to easier access to patient waiting rooms, higher number of accesses in the hospital and higher compliance.

109 breast patients from a comprehensive cancer center in northern Italy and 104 family caregivers were recruited for the study. Four caregivers were not able to come to the consultation on the scheduled day and thus could not provide informed consent; their responses therefore were not considered for the data analysis. The procedure of recruitment was the same as for the first study.

*2.2 Materials*

The final version of the FaRE obtained at the end of Study 1, which consisted of 24 items, was administered to patients and caregivers (see Supplementary Material). As in Study 1, the RSA scale [22] was chosen to assess the convergent validity of the FaRE.

*2.3 Statistical analyses*

SAS software version 9.2 was used to conduct all statistical analyses. Confirmative Factorial Analysis (CFA) was conducted on the final version of the questionnaire. Multigroup factor analysis was conducted to test for factorial invariance across patients and caregivers. Wilcoxon signed rank tests were conducted to evaluate possible differences in clinical and sociodemographic characteristics between the two sample and differences in factors between Study 1 and 2.

In order to assess construct validity of FaRE, we analyzed whether the subscales of the questionnaire correlated with the RSA using Spearman correlations.



For peer review only - http://bmjopen.bmj.com/site/about/guidelines.xhtml

Finally, we tested the model separately in each of the group, caregivers and patients, and tested the goodness of fit of our four-factor model with a more conservative, three-factor model, to compare differences in practical fit indices and evaluate whether strong factorial invariance, which requires a change in RMSEA of less than .015 or a change in CFI of less than .01 [28] is supported.

*2.4 Results*

*2.4.1 Descriptive statistics*

The median age of the population was 49 and IQR from 43 to 56. Thirty-three percent of the caregivers were female. Most participants were married or in a relationship (80% and 74% for patients and caregivers respectively) and had children (95% and 92% for patients and caregivers respectively). The majority of the participants had a high school degree or higher (82% for patients, 82% for caregivers). More than half of the patients were not receiving oncological therapy at time of recruitment and were diagnosed with Stage I-II breast cancer. Further descriptive information can be found in Table 3.

Table 3. Descriptive statistics of the second sample

| | | Overall (N=213) | Patients (n=109) | Caregivers (n=104) |
|---|---|---|---|---|
| Age | <50, n (%) | 116 (54) | 109 (51) | 104 (49) |
| | ≥50, n (%) | 97 (46) | 60 (55) | 56 (54) |
| Children | 0, n (%) | 47 (22) | 49 (45) | 48 (46) |
| | 1-2, n (%) | 153 (72) | 22 (20) | 25 (24) |
| | >3, n (%) | 13 (6) | 82 (75) | 71 (68) |
| Marital status | Single*, n (%) | 44 (21) | 20 (18) | 24 (23) |
| | Married, n (%) | 164 (77) | 87 (80) | 77 (74) |
| | Missing, n (%) | 5 (2) | 2 (2) | 3 (3) |
| Educational level | Elementary-middle school, n (%) | 38 (18) | 20 (18) | 18 (17) |
| | High school, n (%) | 92 (43) | 41 (38) | 51 (49) |
| | University, n (%) | 82 (38) | 48 (44) | 34 (33) |
| | Missing, n (%) | 1 (0) | 0 (0) | 1 (1) |
| Stage | 0, n (%) | | 11 (10.1) | |
| | I, n (%) | | 52 (47.7) | |
| | II, n (%) | | 25 (22.9) | |
| | III, n (%) | | 20 (18.8) | |
| Previous therapy | Surgery, n (%) | | 42 (38.5) | |



For peer review only - http://bmjopen.bmj.com/site/about/guidelines.xhtml

| | | |
|---|---|---|
| | Chemotherapy, n (%) | 19 (17.4) |
| | Hormone therapy, n (%) | 2 (1.8) |
| | No therapy, n (%) | 46 (42.2) |
| Ongoing therapy | Chemotherapy, n (%) | 16 (14.7) |
| | Hormone therapy, n (%) | 13 (11.9) |
| | Radiotherapy, n (%) | 10 (9.2) |
| | No therapy, n (%) | 70 (64.2) |

*Single: never married or widow or separated

*2.4.2 Confirmatory factorial analysis*

Weak factorial invariance suggested acceptable measurement invariance (patients SRMR =0.079, caregivers SRMR= 0.080). The Bentler CFI was 0.90, the RMR Estimate 0.07, indicating an adequate model of fit between the model and the data ($\chi^2$=3139.36, DF=276, *p*<.001). When testing the goodness of fit of our four-factor model with a more conservative, three-factor model, results yielded similar fit indices. The three-factor model had a Bentler CFI equal to 0.951 and RMSEA Estimate equal to 0.0516 ($\chi^2$= 2044.46, DF=190, *p*<.0001), while the four-factor model's Bentler was CFI 0.942 and RMSEA Estimate was 0.0513 ($\chi^2$= 2571.16, DF=276, *p*<.0001).

Difference between the two models in RMSEA scores was 0.00003 and in CFI was 0.009, providing evidence for strong factorial invariance [31].

*2.4.3 Construct validity*

Total scores of FaRE and RSA were positively correlated (ρs= 0.43, *p*<0.0001). Factor Communication and cohesion correlated positively with factor Family Cohesion in RSA (ρs= 0.56, *p*<0.0001). Factor Perceived social support correlated positively with factor Social Resources (ρs= 0.54, *p*<0.0001). Factor Perceived family coping correlated positively with factor Structured Style (ρs= 0.30, *p*<0.0001) and with factor Perception of Self in RSA (ρs= 0.42, *p*<0.0001).

*2.4.5 Differences between Study 1 and Study 2 samples*



For peer review only - http://bmjopen.bmj.com/site/about/guidelines.xhtml

Wilcoxon signed rank tests indicated no differences between the median values of factors in sample in Study 1 and the sample in Study 2, both when considering together the patients and the caregivers, as well as when the patients and the caregivers were compared separately.

*2.4.6 Normative family resilience score*

Due to disparity in number between prostate and breast cancer patients, normative data was calculated on the total breast cancer patients recruited for the two studies (N=317). Mean (5.7) and standard deviation (0.78) for the total FaRE score was calculated. As with other clinical measures, scores between -/+ 1 SD can be considered in the normal range, scores between -1 and -2 SD could indicate lower levels of family resilience and scores under 2 SD could be considered clinically relevant. For the last two ranges of scores referral to support services would be recommended.

## 3. Discussion

The FaRE questionnaire is designed to measure family resilience in oncological settings. Adopting a systemic approach, it considers both the patient and the caregiver's strengths and resources in managing the disease. Our initial version of the questionnaire had the same six family resilience constructs addressed by Sixbey[15]; however, a six-factor solution was not supported and it was reduced to four factors. Compared to Sixbey's[15] questionnaire, the FaRE does not include "Making meaning of adversity" and "Positive outlook"; however, the latter is addressed in the Perceived family coping subscale of the FaRE. Sixbey's Connectedness construct is incorporated in Family Communication and Cohesion. While Lane's [32] family resilience measure had highlighted that socio-economic resources had little value to breast cancer survivors; our measure suggests that social resources provide an invaluable support to the family coping with cancer and that economic resources were unrelated to the social ones. The development of the current questionnaire confirms that different resources are activated when families face cancer compared to other adversities and/or the general population.



For peer review only - http://bmjopen.bmj.com/site/about/guidelines.xhtml

An important aspect to keep in mind is the low inter-rater reliability between patients and caregivers and this is partly explained by the significant differences found between some FaRE factors. Patients perceived significantly higher levels of family coping and higher levels of social support compared to their caregivers; although the difference in scores was small, it could be explained by the re-direction and allocation of shared coping strategies towards the patient rather than towards the caregiver. This suggests that it is vital to consider the pluralistic view of family members as they are actively involved in cancer management. Further research should try and disentangle whether lower support and family coping scores of caregivers are of clinical significance, for instance by assessing the association with mood profiles.

Recruitment of participants from eight different regions of Italy and the combination of cognitive interviews and expert review, which allowed us to determine the relevance of the items, are some of the strengths of the study. Another strength of the developed measure is its systemic view, which allows use in caregiver populations, for whom there are only a few questionnaires available, possibly because attention towards their challenges and needs has increased only in the last few years [33,34].

This said there were some limitations in the development of the current questionnaire. The sample size of the first study is relatively small considering the number of items of the first version of the FaRE; a larger sample could have highlighted missing aspects. Convergent and discriminant validity examining similarities and differences between the FaRE and other family resilience tools would strengthen the rigor of the questionnaire. In addition to this, the cut-off points of the total FaRE scores need validating in future studies in order to use them as screening for referral. In addition to this, it is important to evaluate whether the religiousness-spirituality subscale should be included in the total score of family resilience and whether it plays a key role in promoting resilience levels. To ensure generalisability of the results, a larger sample with diverse tumour diagnosis should be recruited, as our sample was composed of only gender-related tumours with a relatively high life expectancy; in addition to this patients with other chronic and acute illnesses should be sampled to evaluate whether



For peer review only - http://bmjopen.bmj.com/site/about/guidelines.xhtml

important family resilience aspects in disease management have been dropped. This instrument can be a useful tool to assess patients at baseline and for continued follow-up, for this reason test-retest reliability and responsiveness to change should be conducted.

Preliminary evidence shows that the FaRE is a reliable and valid systemic measure of family resilience in oncological settings. The tool was designed to be implemented in a web platform as a part of the works of an European project, which aimed at promoting cancer patient's empowerment and self-management [35–37]. As increasing attention is being paid to family resilience and its protective role in illness trajectories, once further validations are conducted, it can serve as a useful screening tool to identify patients and carers with few resources to deal with cancer related issues. Family resilience screening would allow targeted interventions on development and promotion of specific strategies and resources that can help the family deal with the illness.

**Funding**

This project has received funding from the European Union's Horizon 2020 research and innovation programme under grant agreement No 643529.

**Disclaimer**

This presentation reflects the authors' view. The Commission is not responsible for any use that may be made of the information it contains.

**Declaration of interest**

We have read and understood the BMJ Group policy on declaration of interests and we declare no conflict of interest.

**Author Contributions**

FF has made substantial contributions to article conception and design, was involved in recruitment of patients and in data entry, has given final approval of the version to be published. SG has



For peer review only - http://bmjopen.bmj.com/site/about/guidelines.xhtml

contributed to the analysis and interpretation of data for the work, to drafting the work and provided a critical revision for important intellectual content, has given final approval of the version to be published. CR has undertaken the literature review to construct the measure, has developed the items of the questionnaire, has been involved in drafting the article, has given final approval of the version to be published. CF has undertaken the literature review to construct the measure, has developed the items of the questionnaire, has given final approval of the version to be published. CC was involved in recruitment of patients, data entry, has given final approval of the version to be published. GP has revised the article critically for important intellectual content, has given final approval of the version to be published.

**Data sharing statement**

Data leading to the results presented in this manuscript are available upon reasonable request from Sara Gandini: sara.gandini@ieo.it

**Acknowledgements**

We thank patients for providing feedback and suggestions for improving the family resilience tool.

**Figure legends**

Figure 1. Eingenvalues and proportion of variance of the factors

For peer review only

## References

1. Rutter M. Implications of resilience concepts for scientific understanding. *Ann N Y Acad Sci*. 2006;1094:1-12. doi:10.1196/annals.1376.002

2. Masten AS. Ordinary magic: Resilience processes in development. *Am Psychol*. 2001;56(3):227-238. doi:10.1037//0003-066X.56.3.227

3. Windle G. What is resilience? A review and concept analysis. *Rev Clin Gerontol*. 2011;21(2):152-169. doi:10.1017/S0959259810000420

4. Strauss B, Brix C, Fischer S, et al. The influence of resilience on fatigue in cancer patients undergoing radiation therapy (RT). *J Cancer Res Clin Oncol*. 2007;133(8):511-518. doi:10.1007/s00432-007-0195-z

5. Wenzel LB, Donnelly JP, Fowler JM, et al. Resilience, reflection, and residual stress in ovarian cancer survivorship: A gynecologic oncology group study. *Psychooncology*. 2002;11(2):142-153. doi:10.1002/pon.567

6. Min JA, Yoon S, Lee CU, et al. Psychological resilience contributes to low emotional distress in cancer patients. *Support Care Cancer*. 2013;21(9):2469-2476. doi:10.1007/s00520-013-1807-6

7. Von Bertalanffy L. General system theory. In: *The Science of Synthesis: Exploring the Social*

For peer review only

*Implications of General Systems Theory*. Boulder, Colorado: University Press of Colorado; 2003:304.

8. Van Schoors M, Caes L, Verhofstadt LL, Goubert L, Alderfer MA. Systematic review: Family resilience after pediatric cancer diagnosis. *J Pediatr Psychol*. 2015;40(9):856-868. doi:10.1093/jpepsy/jsv055

9. Walsh F. Family Resilience: A Framework for Clinical Practice. *Fam Process*. 2003;42(I):1-18. doi:10.1111/famp.2003.42.issue-1

10. Faccio F, Renzi C, Giudice A V., Pravettoni G. Family Resilience in the Oncology Setting: Development of an Integrative Framework. *Front Psychol*. 2018;9(666). doi:10.3389/fpsyg.2018.00666

11. McCubbin M, McCubbin HI. Families coping with illness: The resiliency model of family stress, adjustment, and adaptation. *Fam Heal Illn Perspect coping Interv*. 1993:21-63.

12. McCubbin MA, McCubbin HI, Thompson AI. Family hardiness index (FHI). In: *Family Assessment: Resiliency, Coping and adaptation–Inventories for Research and Practice*. ; 1996:239-305.

13. Walsh F. Applying a Family Resilience Framework in Training, Practice, and Research: Mastering the Art of the Possible. *Fam Process*. 2016;55(4):616-632. doi:10.1111/famp.12260

14. Rolland JS, Walsh F. Facilitating family resilience with childhood illness and disability. *Curr Opin Pediatr*. 2006;18(5):527-538. doi:10.1097/01.mop.0000245354.83454.68

15. Sixbey MT. Development of the family resilience assessment scale to identify family resilience constructs. *Dr Diss Univ Florida*. 2005.

16. Isaacs SA, Roman NV, Savahl S, Sui X-C. Adapting and Validating the Family Resilience

For peer review only



For peer review only - http://bmjopen.bmj.com/site/about/guidelines.xhtml

Assessment Scale in an Afrikaans Rural Community in South Africa. *Community Ment Health J*. 2017;0(0):0. doi:10.1007/s10597-017-0091-1

17. Duca DS. Family resilience and parental stress: the effects on marital relationship in the context of a child diagnosed with an autism spectrum disorder. *Ann AII Cuza Univ Psychol Ser*. 2015;24(1):71-91. http://repository.upenn.edu/edissertations_sp2%0Ahttp://repository.upenn.edu/edissertations_sp2/14.

18. Chew J, Haase AM. Psychometric properties of the Family Resilience Assessment Scale: A Singaporean perspective. *Epilepsy Behav*. 2016;61:112-119. doi:10.1016/j.yebeh.2016.05.015

19. Walsh F. *Strengthening Family Resilience*.; 2013. doi:10.1086/514469

20. Lane CD, Meszaros PS. Measuring Walsh ' s Family Resilience Framework : Reliability and Validity of the Family Resilience Assessment ( FRA ) Among Women With a History of Breast Cancer. *Marriage Fam Rev*. 2016:1-16.

21. Messick S. Validity of psychological assessment: validation of inferences from persons' responses and performances as scientific inquiry into score meaning. *ETS Res Rep Ser*. 1994;1994(2):i-28. doi:10.1002/j.2333-8504.1994.tb01618.x

22. Capanna C, Stratta P, Hjemdal O, Collazzoni A, Rossi A. The Italian validation study of the Resilience Scale for Adults (RSA). *Appl Psychol Bull*. 2015;272(63):16-24. http://search.ebscohost.com/login.aspx?direct=true&db=psyh&AN=2015-19177-002&site=ehost-live&scope=site.

23. Fabrigar LR, Wegener DT, Maccallum RC, Strahan EJ. Evaluating the Use of Exploratory Factor Analysis in Psychological Research. *Psychol Methods*. 1999;4(3):272-299.

For peer review only



For peer review only - http://bmjopen.bmj.com/site/about/guidelines.xhtml

24. Hatcher RL, Barends A, Hansell J, Gutfreund MJ. Patients' and Therapists' Shared and Unique Views of the Therapeutic Alliance: An Investigation Using Confirmatory Factor Analysis in a Nested Design. *J Consult Clin Psychol*. 1995;63(4):636-643.

25. Cerny BA, Kaiser HF. A Study Of A Measure Of Sampling Adequacy For Factor-Analytic Correlation Matrices. *Multivariate Behav Res*. 1977;12(1):43-47. doi:10.1207/s15327906mbr1201_3

26. Fornell C, Larcker DF. Evaluating Structural Equation Models with Unobservable Variables and Measurement Error. *J Mark Res*. 1981;18(1):39-50. doi:10.1177/002224378101800104

27. Raykov T. Estimation of congeneric scale reliability using covariance structure analysis with nonlinear constraints. *Br J Math Stat Psychol*. 2001;54(2):315-323. doi:10.1348/000711001159582

28. Hu L, Bentler PM. Cutoff criteria for fit indexes in covariance structure analysis: Conventional criteria versus new alternatives. *Struct Equ Model A Multidiscip J*. 1999;6(1):1-55. doi:10.1080/10705519909540118

29. DeVon HA, Block ME, Moyle-Wright P, et al. A Psychometric Toolbox for Testing Validity and Reliability. *J Nurs Scholarsh*. 2007;39(2):155-164. doi:10.1111/j.1547-5069.2007.00161.x

30. Koo TK, Li MY. A Guideline of Selecting and Reporting Intraclass Correlation Coefficients for Reliability Research. *J Chiropr Med*. 2016;15(2):155-163. doi:10.1016/j.jcm.2016.02.012

31. Chen FF. Sensitivity of Goodness of Fit Indexes to Lack of Measurement Invariance. *Struct Equ Model A Multidiscip J*. 2007;14(3):464-504. doi:10.1080/10705510701301834

32. Meszaros PS, Allen KR, Few-demo AL, Piercy FP, Savla JS. Tracing the Pink Ribbon : Development of a Family Resilience Measure Crystal Lynn Duncan Lane Dissertation




For peer review only - http://bmjopen.bmj.com/site/about/guidelines.xhtml

submitted to the faculty of the Virginia Polytechnic Institute and State University in partial fulfillment of the requirements of the degree of Doct. 2011.

33. Kim Y, Baker F, Spillers RL, Wellisch DK. Psychological adjustment of cancer caregivers with multiple roles. *Psychooncology*. 2006;15(9):795-804. doi:10.1002/pon.1013

34. Sklenarova H, Krümpelmann A, Haun MW, et al. When do we need to care about the caregiver? Supportive care needs, anxiety, and depression among informal caregivers of patients with cancer and cancer survivors. *Cancer*. 2015;121(9):1513-1519. doi:10.1002/cncr.29223

35. Kazantzaki E, Koumakis L, Kondylakis H, et al. Current trends in Electronic Family Resilience Tools: Implementing a tool for the cancer domain. In: ; 2018:29-32. doi:10.1007/978-981-10-5122-7_8

36. Renzi C, Fioretti C, Mazzocco K, et al. Development of psycho-emotional monitoring tools within an eHealth platform to improve patient empowerment and self-management abilities. *Psychooncology*. 2016;25(3):195.

37. Faccio F, Renzi C, Crico C, et al. Development of an eHealth tool for cancer patients: monitoring psychoemotional aspects with the family resilience (FaRe) questionnaire. *Ecancermedicalscience*. 2018;12. doi:10.3332/ecancer.2018.852

For peer review only

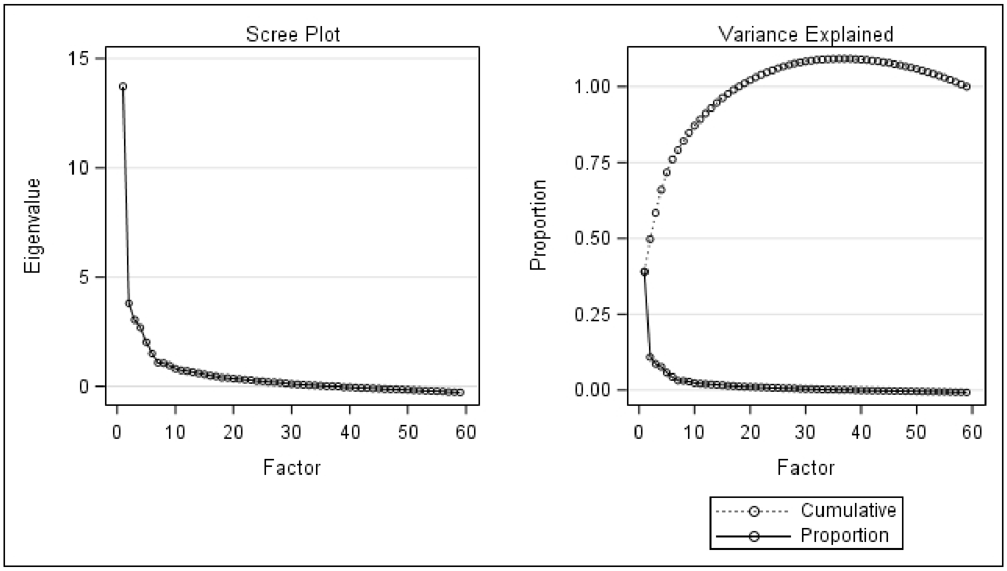


Figure 1. Eigenvalues and proportion of variance of the factors

# Supplementary Material

Appendix A. *Family Resilience Questionnaire*

Dear Sir/Madam,

Please read each of the following statements carefully. For each one, state how well it describes your family. In thinking about your family, you can include all the significant people you think are part of it.

1. We understand each other with regard to the experience of illness we are living

Strongly disagree Moderately disagree Slightly disagree Nor Disagree nor agree Slightly Agree Moderately Agree Strongly agree

2. We ask our closest friends to help and assist us in this battle against the illness

Strongly disagree Moderately disagree Slightly disagree Nor Disagree nor agree Slightly Agree Moderately Agree Strongly agree

3. We believe that we can manage the illness

Strongly disagree Moderately disagree Slightly disagree Nor Disagree nor agree Slightly Agree Moderately Agree Strongly agree

4. In our family we feel that we can talk about how to communicate between us

Strongly disagree Moderately disagree Slightly disagree Nor Disagree nor agree Slightly Agree Moderately Agree Strongly agree

5. We can rely on our close friends to help us deal this illness

Strongly disagree Moderately disagree Slightly disagree Nor Disagree nor agree Slightly Agree Moderately Agree Strongly agree



For peer review only - http://bmjopen.bmj.com/site/about/guidelines.xhtml

6. We attend the church/synagogue/mosque/other places of worship

Strongly disagree Moderately disagree Slightly disagree Nor Disagree nor agree Slightly Agree Moderately Agree Strongly agree

7. We can deal this illness as a family

Strongly disagree Moderately disagree Slightly disagree Nor Disagree nor agree Slightly Agree Moderately Agree Strongly agree

8.We feel that the people in our social network would be happy to support us emotionally in dealing the illness

Strongly disagree Moderately disagree Slightly disagree Nor Disagree nor agree Slightly Agree Moderately Agree Strongly agree

9. We can solve important problems in our life such as this illness

Strongly disagree Moderately disagree Slightly disagree Nor Disagree nor agree Slightly Agree Moderately Agree Strongly agree

10. We discuss the illness-related problems until we find a shared solution

Strongly disagree Moderately disagree Slightly disagree Nor Disagree nor agree Slightly Agree Moderately Agree Strongly agree

11. We feels that our closest friends would be happy to support us emotionally in managing the illness

Strongly disagree Moderately disagree Slightly disagree Nor Disagree nor agree Slightly Agree Moderately Agree Strongly agree

12. We believe there is a supreme spiritual being that will help us deal this illness



For peer review only

Strongly disagree Moderately disagree Slightly disagree Nor Disagree nor agree Slightly Agree Moderately Agree Strongly agree

13. Everyone in the family feels free to express their own opinion regarding the illness

Strongly disagree Moderately disagree Slightly disagree Nor Disagree nor agree Slightly Agree Moderately Agree Strongly agree

14. We know that if we need comfort, our closest friends will be there for us

Strongly disagree Moderately disagree Slightly disagree Nor Disagree nor agree Slightly Agree Moderately Agree Strongly agree

15. We feel we are strong enough to cope with this illness

Strongly disagree Moderately disagree Slightly disagree Nor Disagree nor agree Slightly Agree Moderately Agree Strongly agree

16. We are honest when talking about the illness amongst ourselves

Strongly disagree Moderately disagree Slightly disagree Nor Disagree nor agree Slightly Agree Moderately Agree Strongly agree

17. We know we are important for our friends

Strongly disagree Moderately disagree Slightly disagree Nor Disagree nor agree Slightly Agree Moderately Agree Strongly agree

18. We participate in the activities of our religious community

Strongly disagree Moderately disagree Slightly disagree Nor Disagree nor agree Slightly Agree Moderately Agree Strongly agree



For peer review only - http://bmjopen.bmj.com/site/about/guidelines.xhtml

For peer review only

19. Everyone in the family is open to listening other's opinions regarding the illness

Strongly disagree Moderately disagree Slightly disagree Nor Disagree nor agree Slightly Agree Moderately Agree Strongly agree

20. We receive gifts and favours from our closest friends

Strongly disagree Moderately disagree Slightly disagree Nor Disagree nor agree Slightly Agree Moderately Agree Strongly agree

21. We have the strength to solve our problems

Strongly disagree Moderately disagree Slightly disagree Nor Disagree nor agree Slightly Agree Moderately Agree Strongly agree

22. The things we do for each other in dealing with this illness make us feel part of the family

Strongly disagree Moderately disagree Slightly disagree Nor Disagree nor agree Slightly Agree Moderately Agree Strongly agree

23. Our friends respect our family for how we reacted to the illness

Strongly disagree Moderately disagree Slightly disagree Nor Disagree nor agree Slightly Agree Moderately Agree Strongly agree

24. We ask our religious/spiritual reference figure for advice or words of comfort about the illness

Strongly disagree Moderately disagree Slightly disagree Nor Disagree nor agree Slightly Agree Moderately Agree Strongly agree



For peer review only - http://bmjopen.bmj.com/site/about/guidelines.xhtml

Appendix B. *Tables of biological descriptive statistics of Study 1 and differential scores in patient-caregiver dyads*

Table 1. Tumor characteristics of the first sample

| | | Total (n=104) | Breast (n=53) | prostate (n=51) |
|---|---|---|---|---|
| Lymph nodes | No | 80 (76.9) | 40 (75.5) | 40 (78.4) |
| | Yes | 22 (21.2) | 13 (24.5) | 9 (17.6) |
| | Missing | 2 (1.9) | 0 (0) | 2 (3.9) |
| Metastasis | No | 101 (97.1) | 53 (100) | 48 (94.1) |
| | Yes | 1 (1) | 0 (0) | 1 (2) |
| | Missing | 2 (1.9) | 0 (0) | 2 (3.9) |
| Stage | I | 15 (14.4) | 11 (20.8) | 4 (7.8) |
| | II | 52 (50) | 33 (62.3) | 19 (37.3) |
| | III-IV | 35 (33.7) | 9 (17) | 26 (51) |
| | Missing | 2 (1.9) | 0 (0) | 2 (3.9) |
| Grading | 1-2 | 39 (37.5) | 26 (49.1) | 13 (25.5) |
| | 3-4 | 64 (61.5) | 27 (50.9) | 37 (72.5) |
| | Missing | 1 (1) | 0 (0) | 1 (2) |
| Previous therapy | No | 28 (26.9) | 12 (22.6) | 16 (31.4) |
| | Yes | 75 (72.1) | 41 (77.4) | 34 (66.7) |
| | Missing | 1 (1) | 0 (0) | 1 (2) |

Table 2. Differences in family resilience scores in patient-caregiver dyads in the first study

| **Differences pt-CG** | **Median** | **Low** | **Up** |
|---|---|---|---|
| factor1_com | 0 | -0.38 | 0.5 |
| factor2_supp | 0 | -0.5 | 0.75 |
| factor3_coping | 0.13 | -0.38 | 1 |
| factor4_spirit | -0.25 | -1 | 1 |

Low: lower quartile; Up: upper quartile

Factor1_com = Communication and cohesion; Factor2_supp= Perceived social support; Factor3_coping = Perceived family coping; Factor4_spirit = Religiousness and Spirituality

Table 3. Mean differential scores and paired-t test between patient-caregiver dyads in the second study

| | Mean | SD | p-value |
|---|---|---|---|
| **diff_factor1** | **-0.27** | 0.93 | **0.003** |



For peer review only - http://bmjopen.bmj.com/site/about/guidelines.xhtml

| | | | |
|---|---|---|---|
| diff_factor2 | 0.21 | 1.17 | 0.074 |
| diff_factor3 | -0.14 | 1.05 | 0.179 |
| diff_factor4 | 0.27 | 1.72 | 0.111 |

Factor1 = Communication and cohesion; Factor2= Perceived social support; Factor3= Perceived family coping; Factor4 = Religiousness and Spirituality

Table 4. Eingenvalues and proportion of variance of the factors

| | Eigenvalue | Difference | Proportion | Cumulative |
|---|---|---|---|---|
| **1** | **13.72** | **9.92** | **0.39** | **0.39** |
| **2** | **3.81** | **0.77** | **0.11** | **0.50** |
| **3** | **3.04** | **0.35** | **0.09** | **0.58** |
| **4** | **2.69** | **0.66** | **0.08** | **0.66** |
| 5 | 2.02 | 0.52 | 0.06 | 0.72 |
| 6 | 1.51 | 0.42 | 0.04 | 0.76 |
| 7 | 1.08 | 0.03 | 0.03 | 0.79 |
| 8 | 1.06 | 0.09 | 0.03 | 0.82 |
| 9 | 0.96 | 0.15 | 0.03 | 0.85 |

For peer review only
